# Transfer of Electrophilic NH Using Convenient Sources of Ammonia: Direct Synthesis of NH Sulfoximines from Sulfoxides

**DOI:** 10.1002/anie.201602320

**Published:** 2016-04-29

**Authors:** Marina Zenzola, Robert Doran, Leonardo Degennaro, Renzo Luisi, James A. Bull

**Affiliations:** ^1^ Department of ChemistryImperial College London, South KensingtonLondonSW7 2AZUK; ^2^ Department of Pharmacy, Drug SciencesUniversity of Bari “A. Moro”Via E. Orabona 4Bari70125Italy

**Keywords:** hypervalent compounds, nitrogen, reactive intermediates, sulfur, synthetic methods

## Abstract

A new system for NH transfer is developed for the preparation of sulfoximines, which are emerging as valuable motifs for drug discovery. The protocol employs readily available sources of nitrogen without the requirement for either preactivation or for metal catalysts. Mixing ammonium salts with diacetoxyiodobenzene directly converts sulfoxides into sulfoximines. This report describes the first example of using of ammonia sources with diacetoxyiodobenzene to generate an electrophilic nitrogen center. Control and mechanistic studies suggest a short‐lived electrophilic intermediate, which is likely to be PhINH or PhIN^+^.

The transfer of nitrogen atoms is extremely valuable in the preparation of important biologically relevant functional groups. Electrophilic nitrogen‐atom sources including nitrenoids, metal–nitrene equivalents, or oxaziridines are therefore highly valuable synthetic tools.^1^ These reagents commonly require the nitrogen atom to be activated to enhance electrophilicity, and is usually achieved by either adding an electron‐withdrawing N‐protecting group or through preactivation with a leaving group. Consequently, there are very few examples of unprotected electrophilic NH sources, which would be more desirable as it avoids the requirement for additional steps and improves atom economy. Activated reagents such as *O*‐mesitylenesulfonylhydroxylamine (MSH) or *O*‐(2,4‐dinitrophenyl)‐hydroxylamine (DPH) have been employed but these suffer from instability and the required additional steps for their preparation.^2^, ^3^ In an important recent example of NH transfer, Falck, Kurti, and co‐workers reported a direct synthesis of unprotected (i.e. NH) aziridines using DPH with [Rh_2_(esp)_2_] as a catalyst in trifluoroethanol.^4^

An increasingly important use of electrophilic formal nitrene sources is in the synthesis of sulfoximines. Sulfoximines have been the subject of intense interest and recently emerged as exciting motifs for drug discovery programs.^5^ Bayer first examined the sulfoximine group, which was considered an oddity in medicinal chemistry at the time, during the development of BAY 1000394, a pan‐CDK inhibitor currently undergoing clinical trials for cancer in patients with advanced solid tumors (Figure 1).^6^ In comparison to sulfones, sulfoximines have increased polarity, can present improved solubility, and provide an additional point of diversity and chirality to increase molecular complexity.^7^ Their expanding application in drug design is exemplified in compound AZD6738 from AstraZeneca.^8^, ^9^ Moreover, since their discovery in the form of MSO,^10^ sulfoximines have been developed as ligands and auxiliaries for asymmetric synthesis^11^, ^12^ and directing groups in C−H functionalization,^13^, ^14^ as well as used in agrochemical agents such as sulfoxaflor.^15^

**Figure 1:**
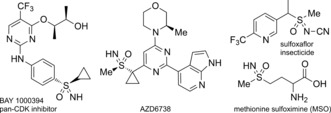
Biologically important sulfoximine‐containing compounds.

Sulfoximines are most often prepared by the transfer of a protected nitrogen group to sulfoxides,^16^ including the transfer of sulfonamide,^17^, ^18^, ^19^ trifluoroacetamide,^17^ carbamate,^20^ and amide groups^21^ using transition‐metal catalysis (Scheme 1 a). These sulfoximines have been deprotected to yield the free NH derivatives, and further functionalized to generate N‐aryl, N‐acyl, and N‐alkyl, as well as cyclic derivatives, offering varying properties.^22^ The direct synthesis of NH sulfoximines has largely involved undesirable reaction conditions, including harsh and explosive reagents.^23^ Recently, a scalable synthesis of NH sulfoximines in continuous flow was reported by Kappe and co‐workers using trimethylsilyl azide and fuming sulfuric acid to transfer an NH group directly to a sulfoxide intermediate of AZD6738, but racemization of the sulfur center occurred.^24^ Richards and co‐workers have demonstrated the use of DPH with rhodium catalysis for the direct preparation of NH sulfoximines under mild reaction conditions (Scheme 1 b).^25^ To date there are no direct methods for the transfer of NH to sulfoxides which use convenient, inexpensive, and safe nitrogen sources. Improved methods for this transfer, methods which avoid harsh reagents, could be widely applied. Herein we report a new process based on commercially available and inexpensive reagents for the stereospecific preparation of NH sulfoximines from sulfoxides using ammonium salts, as the source of NH, with diacetoxyiodobenzene [PhI(OAc)_2_], without the requirement for a precious metal catalyst or base (Scheme 1 c).

**Scheme 1:**
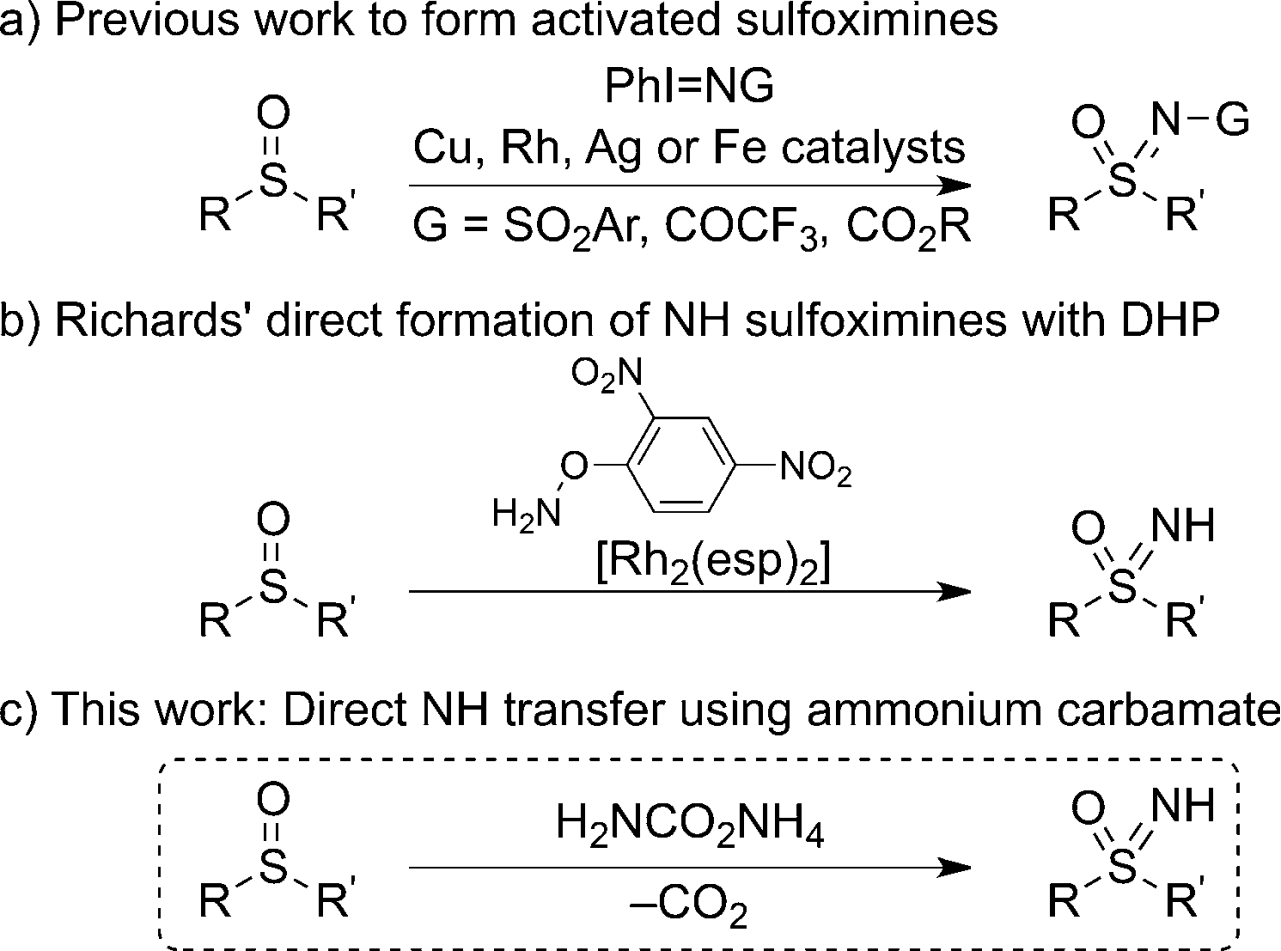
Synthesis of sulfoximines from sulfoxides.

Given our previous success in the transfer of alkyl carbamates to sulfoxides using rhodium catalysis,^20^ we considered that the use of a carbamate salt, which is structurally comparable, might undergo a similar N transfer. Loss of CO_2_ would achieve an overall NH transfer, and as such these reagents would provide a formal nitrene equivalent. Ammonium carbamate was chosen as an inexpensive easily handled solid. This reagent has not previously been used in the formation of electrophilic nitrogen sources, though it was very recently used by Nicewicz and co‐workers as an ammonia source in arene C−H amination under photoredox catalysis.^26^ We initially considered metal‐catalyzed processes for this transformation (using Rh and Fe species) and found high yields of the sulfoximine resulted when starting from methyl *p*‐tolylsulfoxide. However, we were delighted to find that the reaction proceeded equally efficiently in the absence of a metal catalyst. Indeed, after optimization complete conversion to sulfoximine **1 a** was achieved when the reaction was run for 16 h at 35 °C in toluene, by mixing ammonium carbamate with diacetoxyiodobenzene and magnesium oxide (reaction conditions i; Scheme 2 a; see the Supporting Information for details of the optimization).

**Scheme 2:**
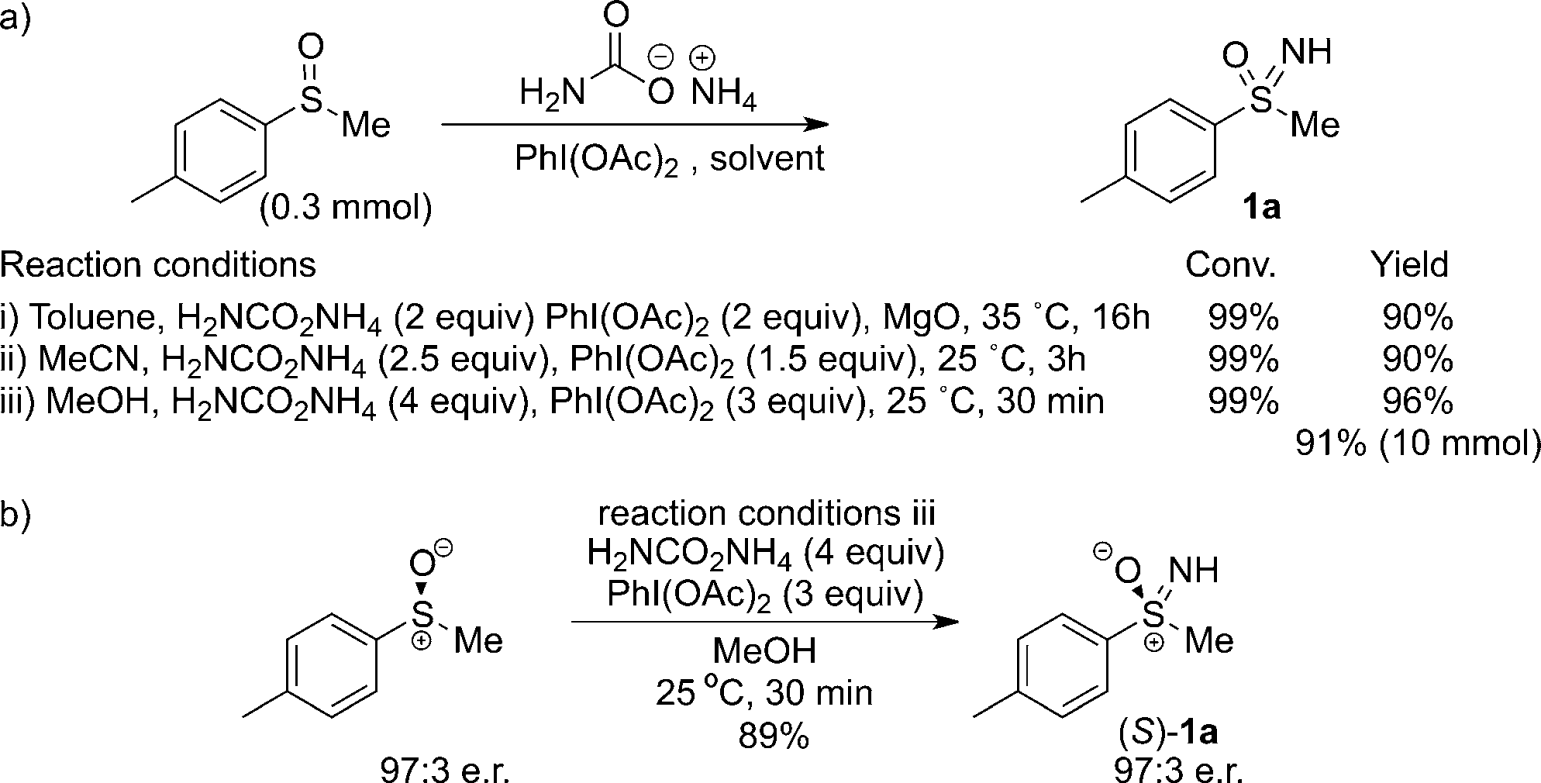
Synthesis of sulfoximines using ammonium carbamate.

With the heterogeneous nature of the reaction in toluene, the rate appeared controlled by the rate of dissolution of ammonium carbamate. Therefore, more polar solvents were investigated to reduce the reaction time. Acetonitrile and methanol were both successful in achieving full conversion by varying the equivalents of ammonium carbamate, without additional base (Scheme 2). In acetonitrile the reaction gave full conversion with 1.5 equivalents of PhI(OAc)_2_ at 25 °C (reaction conditions ii), though different results were obtained when running the reaction in sealed vessels compared to those in open flasks; those run in an open flask gave a slight drop in yield. The most convenient reaction conditions with the shortest reaction time were with MeOH, and full conversion was achieved at 25 °C within 30 minutes when using 3 equivalents of PhI(OAc)_2_ and 4 equivalents of ammonium carbamate (reaction conditions iii). Under these reaction conditions the reaction was performed in an open flask, with decarboxylation evident, and scalability was demonstrated by performing the reaction on a 10 mmol scale (**1 a**, 91 %). The stereochemistry of the process was evaluated using the readily accessible enantioenriched methyl *p*‐tolyl sulfoxide (e.r.: 97:3). The reaction occurred stereospecifically with retention of configuration, thus providing the corresponding enantioenriched sulfoximine (*S*)‐**1 a** (Scheme 2 b).

The reaction scope was examined by using reaction conditions iii (MeOH; Table 1). For certain substrates either the MeCN or toluene reaction conditions were used depending on solubility and to provide a comparison of the different reaction conditions. Aryl‐substituted (R^1^) sulfoxides proved to be generally excellent substrates for this transformation. Phenyl, tolyl, *p*‐chlorophenyl, and *p*‐bromophenyl examples were all obtained in excellent yields. The scope with respect to R^2^ was also good, and included isopropyl, ethyl, ethylphenyl, benzyl, and haloalkyl groups. A yield of 69 % was observed for the phenyl fluoromethyl sulfoximine **1 c** using the methanol reaction conditions. The slightly lower yield resulted from the electron‐withdrawing nature of the fluoromethyl substituent, but it was improved (78 %) by using the acetonitrile reaction conditions. Performing the reaction on enantioenriched substrates gave the enantioenriched sulfoximines **1 b**,**h** with complete retention of configuration [(*R*)‐**1 b**, e.r.: 98:2, (*R*)‐**1 h**, e.r. >99:1]. For a secondary alkyl chloride substrate, the d.r. of the sulfoxide (9:1) was retained in the sulfoximine **1 i**. The acetophenone derivative **1 j** showed the tolerance of the reaction towards aromatic ketones (64 % yield). Phenylvinylsulfoxide was a viable substrate, as was diphenyl sulfoxide, thus leading to the corresponding sulfoximines **1 l** and **1 m**.

**Table 1:** Substrate scope for the NH transfer from the corresponding sulfoxides.^[a]^ 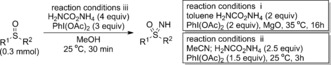

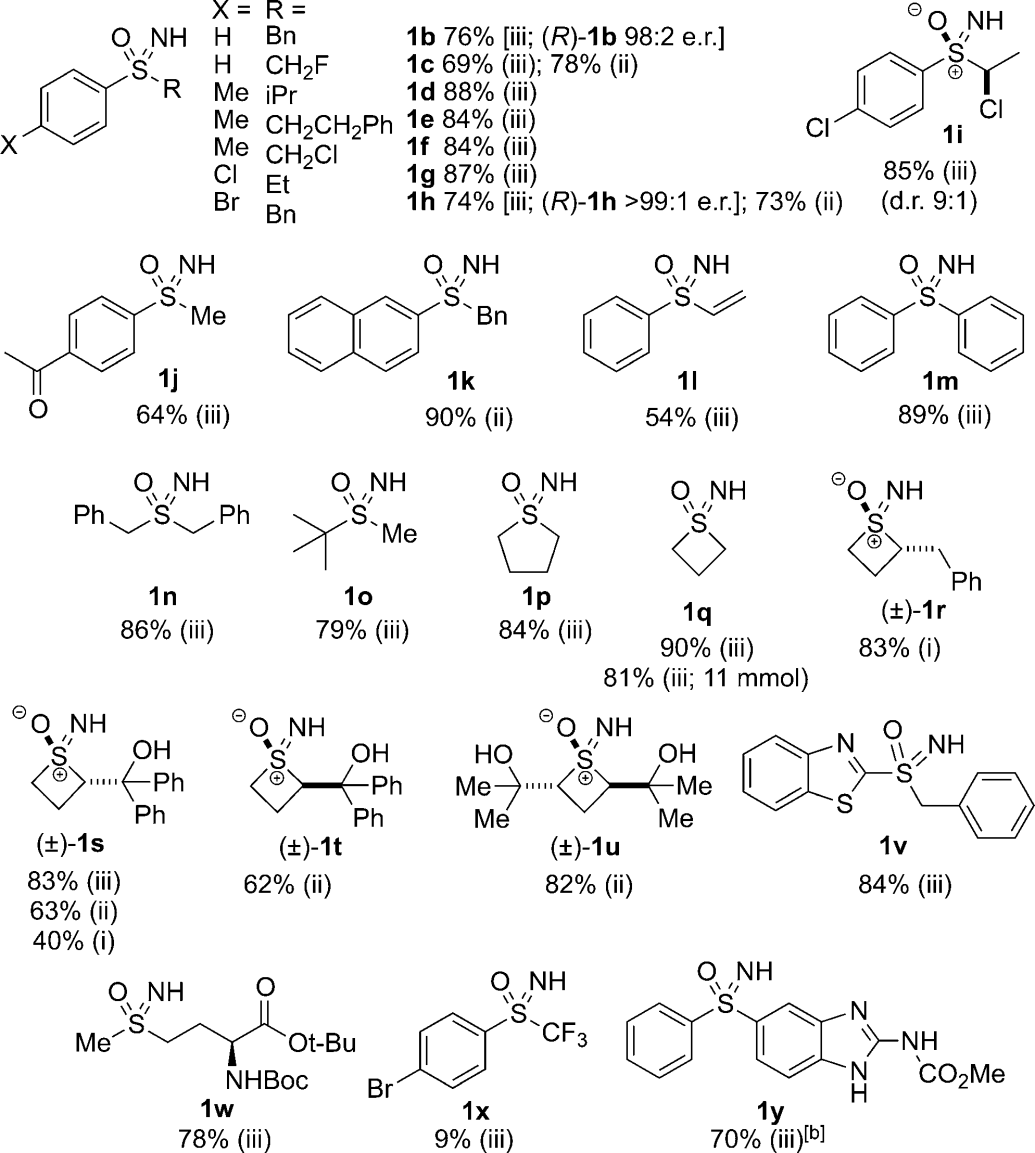

[a] Three sets of reaction conditions (i–iii) were examined. Yield is that of the isolated product. [b] 8 equiv H_2_NCO_2_NH_4_, 4 equiv of PhI(OAc)_2_, 24 h. Boc=*tert*‐butoxycarbonyl.

Upon extending the scope with to dialkyl sulfoxides, high yields were also obtained, as demonstrated by the dibenzylsulfoximine **1 n**, the sterically bulky *tert*‐butylmethyl sulfoximine **1 o**, and the cyclic thiophene and thietanesulfoximines **1 p** and **1 q**, respectively (Table 1). The thietanesulfoximine **1 q** was prepared on an 11 mmol scale in similarly good yield (81 %). Several unusual substituted thietane oxides were also suitable substrates.^27^, ^28^ The benzyl‐substituted example **1 r** was obtained from the corresponding sulfoxide in very good yields by using reaction conditions i. Diastereomerically pure tertiary‐alcohol‐containing thietane oxides led stereospecifically to the sulfoximines **1 s**–**u**. A very good yield was obtained for the benzothiazole‐substituted sulfoximine **1 v**. Furthermore, protected methionine sulfoxide gave the derivative **1 w** in a formal synthesis of MSO (see Figure 1).^29^ A sulfoxide bearing the electron‐withdrawing trifluoromethyl group appeared to be at the limit of reactivity for this methodology, thus affording a 9 % yield of the *p*‐bromophenyl trifluoromethyl sulfoximine **1 x**. Oxfendazole, a benzimidazole‐containing anthelmintic, gave the corresponding sulfoximine **1 y** in 70 % under a modified set of reaction conditions, which were required because of the low solubility of the sulfoxide.

Under these reaction conditions, the reaction was highly selective for the formation of sulfoximines, rather than reaction with other functionalities. To demonstrate this selectivity, we performed a reaction robustness screen, based on the protocol described by Glorius and co‐workers,^30^ as it was designed to assess the tolerance of a reaction to added functional groups and their stability to the reaction conditions. We screened the range of heterocycles suggested by Glorius and we found the reaction to be highly tolerant of the vast majority of these heterocycles (see Table S4 in the Supporting Information). Only two out of the 15 heterocycles tested, *N*‐benzylpyrrole and *N*‐methylindole, gave less than 85 % yield with the majority yielding greater than 95 %. Importantly, heterocycles with basic nitrogen groups did not interfere with the reaction. Excellent results in the presence of pyridine, pyrimidine, quinolone imidazole, thiazole, benzoxazole, and benzothiazole groups were achieved. Electron‐rich aromatic groups were less well tolerated. In many cases the reaction proceeded well, but with poor recovery of the added indole, furan, and thiophene groups. An N‐benzyl‐protected pyrrole was detrimental to the reaction, yet the N‐Boc example was much better tolerated. Gratifyingly, the reaction proved remarkably tolerant of the nine functional‐group additives tested. Alkene, alkyne, alkyl amine, phenol, ester, aldehyde, and nitrile additives all gave greater than 96 % yields with only aniline giving a more moderate yield of 65 %. The recovery of the additive was also good with the exception of benzaldehyde. These results demonstrate a high degree of compatibility of the reaction conditions for a wide range of pharmaceutically relevant functionality.

We proposed that the role of the ammonium carbamate was simply to provide a convenient source of ammonia (Scheme 3 a; see the Supporting Information). The reaction would be faster in methanol not only because of the increased solubility of the ammonium carbamate, but also because PhI(OAc)_2_ undergoes a fast ligand exchange reaction with the solvent releasing acetic acid which accelerates the decomposition of the ammonium carbamate.^31^ Consistent with this hypothesis we were pleased to observe successful reaction through the use of a solution of ammonia in methanol. Complete conversion was obtained using 4 equivalents of NH_3_ (Scheme 3 b). Interestingly, the ammonium acetate, which is also likely to be formed in the reaction with ammonium carbamate, also provided a suitable source of ammonia, thus giving almost complete conversion with 8 or 4 equivalents (Scheme 3 c, reaction conditions iv). By using these reaction conditions with ammonium acetate, also a conveniently handled solid, additional sulfoxides were examined and afforded the sulfoximines (*S*)‐**1 a**, **1 p**, and **1 w** in good yields (Scheme 3 c). Ammonium chloride was also tested but the expected sulfoximine was not observed, whereas the reaction with aqueous ammonia resulted in complete recovery of the starting sulfoxide.

**Scheme 3:**
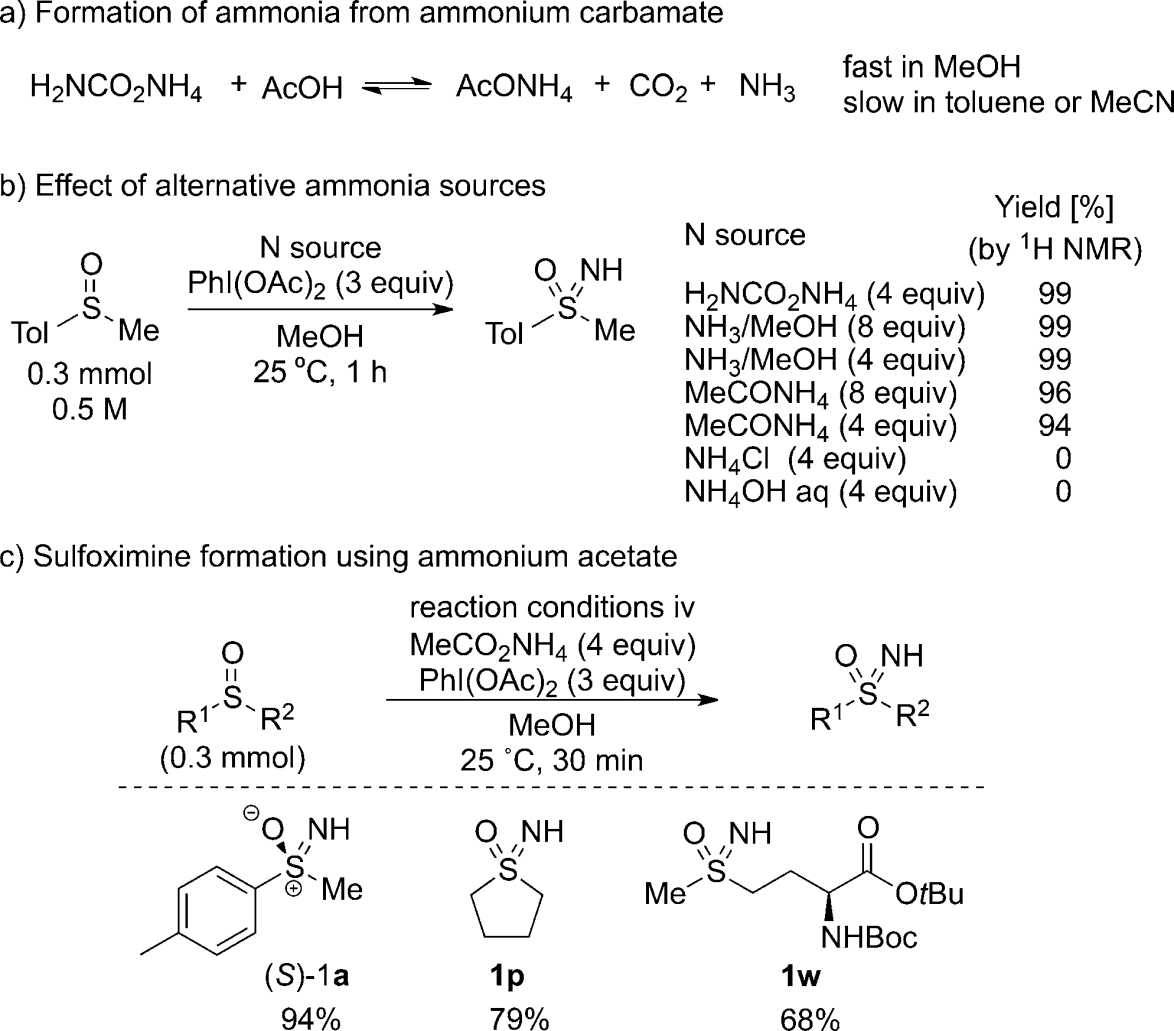
Alternative ammonia sources led to successful reaction.

Initially we considered it likely that the reaction proceeded via the unprecedented iminoiodinane PhI=NH, which may be better represented as a charge‐separated ylide with an I→N dative bond.^32^ Seeking insight into the mechanism and evidence of the iminoiodinane species, we performed an extensive NMR investigation to monitor the reaction in deuterated toluene, acetonitrile, and methanol (see the Supporting Information). The reaction progressed to completion in each case, and was clearly faster in [D_4_]MeOH and [D_3_]MeCN than in toluene. In all cases, the only detectable species derived from the sulfoxide under the full reaction conditions was an iodonium salt (**2**) which collapsed to the sulfoximine over time (Scheme 4).^33^ Independently mixing the isolated sulfoximine with PhI(OAc)_2_ generated the same **2**, containing an I−N single bond, which we have characterized by NMR spectroscopy and mass spectrometry (see the Supporting Information). We proposed that while the reaction was still progressing the sulfoximine product reacted very rapidly with excess PhI(OAc)_2_ to give **2** (Scheme 4). Using a longer reaction time or working up the reaction either directly by evaporation (from the reaction in MeOH) or through a simple aqueous base extraction caused a complete breakdown of this species to give only the desired sulfoximine.

**Scheme 4:**
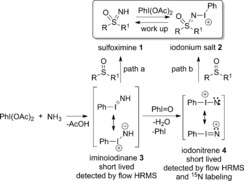
Proposed reaction mechanism involving a reactive iminoiodinane or iodonitrene.

Throughout these NMR studies there was no evidence of any reactive intermediate.^34^ Therefore, to further probe the mechanism, we performed mass spectrometry analysis under flow conditions.^35^ On mixing PhI(OAc)_2_ and ammonium carbamate, signals consistent with the iminoiodinane **3** (PhI=NH; Scheme 4) were observed, thus providing the first evidence for this species, as well as the unprecedented iodonitrene **4** (PhI=N^+^; see the Supporting Information), and support for their involvement as reaction intermediates. However, after addition of the sulfoxide, again only **2** was detected. In another experiment, with the aim to probe the identity of **4**, ^15^N‐labeled ammonia was employed. By using ^15^N‐labeled ammonium acetate, the expected isotopic shift for **4** was observed (see the Supporting Information). Finally, a radical pathway for this reaction was shown to be unlikely as the reaction proceeded successfully in the presence of radical inhibitors (see the Supporting Information).

As a result of these studies, we propose that the ammonium carbamate or acetate provides a sufficient concentration of ammonia to react with PhI(OAc)_2_ to form either **3** or **4**, which are short lived and sufficiently electrophilic to react directly with the sulfoxide and afford the sulfoximine. It is worth mentioning, that in all the MS experiments large amounts of iodosylbenzene (PhI=O) were also detected. It was assumed that **4** could derive from **3** by iodosylbenzene‐promoted oxidation. In Scheme 4, two possible pathways are proposed and involve the intermediates **3** and **4**. With the involvement of **3**, the formation of the sulfoximine may occur by either direct attack of the sulfoxide at the nitrogen atom, with displacement of iodobenzene,^33^, ^36^ or attack at the iodine center, and a subsequent reductive elimination sequence leading to the sulfoximine.^37^ In the case of the **4**, a direct nucleophilic attack of the sulfur atom at the electrophilic nitrogen atom would lead directly to **2**, according to NMR and MS data. In both pathways, the nucleophilicity of the sulfoxide is therefore important, thus explaining the reduced reactivity with very electron‐poor sulfoxides.

In summary, we have developed an extremely facile method for the preparation of NH sulfoximines using readily available reagents, without the requirement for precious metal catalysts, with ammonium salts as the nitrogen source. Ammonium carbamate or ammonium acetate both provide convenient sources of ammonia, which are less expensive than pre‐prepared solutions of ammonia in methanol, and easily handled as solid reagents. The reaction works efficiently for a wide substrate scope and tolerates a large number of heterocycles and other functionalities. We expect this protocol will be of use to medicinal chemists for the preparation of this valuable functional group and to be readily scalable. A reactive iminoiodinane (or iodonitrene) is generated in situ, thus removing the requirement for stoichiometric activated amine source. This reaction constitutes the first example of electrophilic NH transfer mediated by hypervalent iodine.

## Supporting information

As a service to our authors and readers, this journal provides supporting information supplied by the authors. Such materials are peer reviewed and may be re‐organized for online delivery, but are not copy‐edited or typeset. Technical support issues arising from supporting information (other than missing files) should be addressed to the authors.

Supplementary
