# Supplementary material for: Transfer of Electrophilic NH Using Convenient Sources of Ammonia: Direct Synthesis of NH Sulfoximines from Sulfoxides
Source: Angew Chem Int Ed Engl. 2016 Apr 29;55(25):7203–7. doi: 10.1002/anie.201602320 (PMC5074267; doi:10.1002/anie.201602320)
Supplement: Supplementary file 1 — Supplementary [file ANIE-55-7203-s001.pdf]

Angewandte Chemie
Eine Zeitschrift der Gesellschaft Deutscher Chemiker

Supporting Information

Transfer of Electrophilic NH Using Convenient Sources of Ammonia: Direct Synthesis of NH Sulfoximines from Sulfoxides

Marina Zenzola[+], Robert Doran[+], Leonardo Degennaro, Renzo Luisi, and James A. Bull**

anie_201602320_sm_miscellaneous_information.pdf

SUPPLEMENTARY INFORMATION

Table of Contents

Full citation for reference 18 .... S1
Optimization of the Reaction Conditions S2
Table S4: Robustness screen S6
Experimental S7
HPLC Analysis S16
Investigation of the Mechanism of the NH Transfer S19
^{1}H and ^{13}C NMR spectra for selected compounds S32
References S55

For a video exemplifying the protocol see: **https://youtu.be/4KpDQnGHi28**

Reference 18:
Y. Zhu, M. R. Loso, G. B. Watson, T. C. Sparks, R. B. Rogers, J. X. Huang, B. C. Gerwick, J. M. Babcock, D. Kelley, V. B. Hegde, B. M. Nugent, J M. Renga, I. Denholm, K. Gorman, G. J. DeBoer, J. Hasler, T. Meade, J. D. Thomas, *J. Agric. Food Chem.* **2011**, 59, 2950–2957.

Optimization of the Reaction Conditions

The reaction was initially optimized in toluene as the solvent (Table 1) before different conditions were developed using MeOH or MeCN as the solvent. The best conditions are reported in bold. The protocol in toluene and MeCN requires a basic aqueous work up in order to transform the iodonium salt, if formed, into the corresponding sulfoximine.

Key

AC = ammonium carbamate = NH_4OCONH_2

DIB = $PhI(OAc)_2$

SM = starting material = sulfoxide

NMR Yield = yields determined by ^{1}H NMR, calculated using 1,3,5-trimethoxybenzene or mesitylene as an internal standard.

Conversion = conversion from starting material based on ^{1}H NMR analysis.

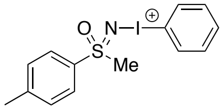

iodonium salt

Supplementary Table 1. Optimization in Toluene. Summary of the early development of the reaction, initially using a Rh catalyst before optimization led to full conversion in the absence of any transition metal catalyst.

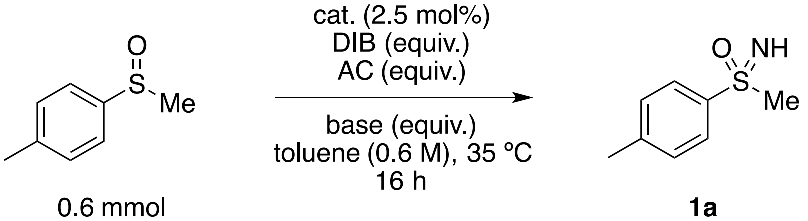

Catalyst	Base (equiv.)	DIB (equiv.)	AC (equiv.)	Conversion (%)	Note
$Rh_2(OAc)_4$	MgO (4)	1.5	1.5	80	40 ºC
$Rh_2(OAc)_4$	MgO (4)	2	2	92	40 ºC
$Rh_2(OAc)_4$	MgO (4)	2	2	95	
$Rh_2(TFA)_4$	MgO (4)	2	2	99	
$Fe(acac)_3$	MgO (4)	2	2	95	
-	MgO (4)	2	2	99	
-	Et_3N (4)	2	2	0	
-	NaOH (4)	2	2	86	
**-**	**MgO (2)**	**2**	**2**	**99**	
-	MgO (1)	2	2	97	
-	0	2	2	92	No base
-	MgO (4)	1.5	1.5	84	
-	MgO (2)	1	2	82	
-	MgO (4)	0	2	0	No $PhI(OAc)_2$
-	MgO (2)	2	0	0	No nitrogen sources
-	MgO (4)	0.15	2	9	Catalytic $PhI(OAc)_2$

Supplementary Table 2.1 Optimization in MeOH. Summary of the optimization of reaction conditions using MeOH as the solvent showing the effect of concentration and reagent equivalents.

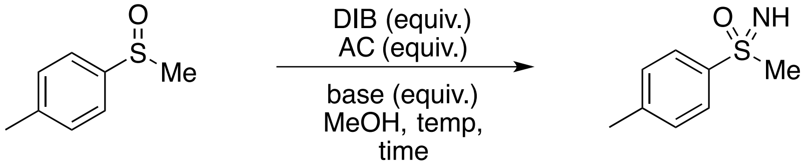

0.1 mmol **1a**

Conc. (M)	Base (equiv.)	DIB (equiv.)	AC (equiv.)	Temp (°C)	time (h)	Conversion/ NMR Yield (%)
0.08	0	1	1	25	24	21
0.08	0	2	2	25	24	57
0.08	0	1.5	4	25	0.5	58*
0.08	0	3	4	25	0.5	77*
0.08	NH_3 in MeOH (2)	3	4	25	0.5	96*
**0.5**	**0**	**3**	**4**	**25**	**0.5**	**98***
0.5	0	2.5	1.5	25	0.5	92*

MeOH added to reaction flask containing DIB, AC and the sulfoxide. * ^{1}H NMR yield.

Supplementary Table 2.2 Effect of Reaction Concentration. This table compares the same reaction conditions at different reaction concentrations to highlight the significant effect it has on the reaction outcome.

Conc. (M)	Base (equiv.)	DIB (equiv.)	AC (equiv.)	Temp (°C)	time (h)	NMR Yield (%)
0.1	Et_3N (2)	3	4	25	0.5	10
0.3	Et_3N (2)	3	4	25	0.5	52
0.5	Et_3N (2)	3	4	25	0.5	60

Et_3N added to reaction mixture before MeOH.

Supplementary Table 2.3 Effect of Temperature. This table shows the effect of the temperature on the reaction outcome, in particular employing reduced reagent equivalents at lower temperatures.

Conc. (M)	Base (equiv.)	DIB (equiv.)	AC (equiv.)	Temp (°C)	time (h)	Conversion/ NMR Yield (%)
0.5	0	3	4	25	0.5	98*
0.5	0	3	4	0	1	99
0.5	0	1.5	2	0 ºC for 3 h, then 25 ºC for 2 h		94*
0.5	0	1.5	2.5	0 ºC for 3 h, then 25 ºC for 2 h		97*
0.5	0	1.5	2.5	0 ºC for 3 h, then 25 ºC for 2 h		97*
		1.5	2.5	0 ºC warmed to 10 °C over 12 h		96*
0.5	0	3	4	-17	3	93*
0.5	0	1.5	2.5	−17 °C for MeOH add$^{\underline{n}}$ then rt, 1 h		95*
0.5	0	1.5	2.5	0 °C for MeOH add$^{\underline{n}}$ then rt, 1 h		92*

All reactions diluted with MeOH, transferred to a RBF and concentrated *in vacuo* at 35 °C. * ^{1}H NMR yield.

Supplementary Table 2.4 Effect of Order of Addition. This table shows how the different order of addition has a noticeable effect on the yields obtained

Conc. (M)	Base (equiv.)	DIB (equiv.)	AC (equiv.)	Temp (°C)	time (h)	NMR Yield (%)	Order of addition
0.5	Et_3N (2)	3	4	25	0.5	60	MeOH added last
0.5	Et_3N (2)	3	4	25	0.5	81	Et_3N added last, 30 sec after MeOH
0.5	0	1.5	2.5	25	0.5	77	DIB added to soln. of SM/AC
0.5	0	1.5	2.5	25	0.5	89	AC added to soln. of SM/DIB
0.5	0	1.5	2.5	25	0.5	92	MeOH added last
0.5	0	3	4	25	0.5	36	SM added after 10 min
0.5	0	3	4	25	0.5	98	MeOH added last

Supplementary Table 2.5 Effect of Other ROH Solvents. A number of other alcohols were screened and compared to MeOH using reduced equivalents to observe if this would effect the stability of the reaction intermediates. No improvement on MeOH was found.

Conc. (M)	Solvent	DIB (equiv.)	AC (equiv.)	Temp (°C)	time (h)	NMR Yield (%)
0.5	MeOH	1.5	2.5	25	3	92
0.5	EtOH	1.5	2.5	25	3	85
0.5	*i*-PrOH	1.5	2.5	25	3	32

Supplementary Table 3. Optimization in MeCN. Summary of the optimization of reaction conditions using MeCN as the solvent showing the effect of concentration and reagent equivalents.

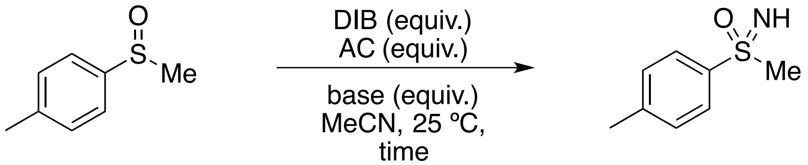

0.3 mmol **1a**

Conc. (M)	DIB (equiv.)	AC (equiv.)	Time (h)	Conversion[a]
0.05	3	4	0.5	10%
0.5	1.5	2.5	0.5	26%
0.5	1.5	2.5	1.5	93%
**0.5**	**1.5**	**2.5**	**3h**	**>99%**
0.5	1.1	1.1	3h	70%
0.5	1.1	2.1	3h	90%

After basic work-up.

Supplementary Table 3.1 Effect of Open vs. Closed Reaction Flask. This table shows a comparison of the reaction carried out in a closed reaction flask and a reaction flask open to the atmosphere

Conc. (M)	DIB (equiv.)	AC (equiv.)	Temp (°C)	time (h)	NMR Yield (%)	Flask
0.5	1.5	2.5	25	3	68	Open flask
0.5	1.5	2.5	25	3	87	Closed μwave vial, ~11 mL total empty volume.
0.5	1.5	2.5	25	3	92	Closed 5 mL RBF, ~7.5 mL total empty volume

Supplementary Table 4. Robustness screen to assess the effect of added functional groups on the reaction with tolylmethylsulfoxide to form sulfoximine 1a.[a]

Additive	Yield of **xx** (%)	Additive remaining (%)	SM remaining (%)	Additive	Yield of **xx** (%)	Additive remaining (%)	SM remaining (%)
None	99 ✓	-	0				
**Heterocycles**							
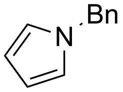	13 ✗	53 −	82	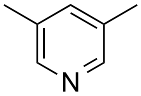	97 ✓	55 −	0
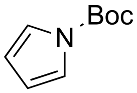	87 ✓	47 −	0	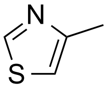	99 ✓	54 −	0
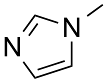	98 ✓	87 ✓	0	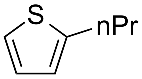	97 ✓	10 ✗	0
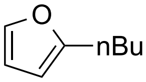	89 ✓	0 ✗	0	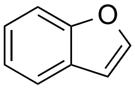	95 ✓	45 −	0
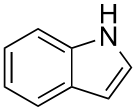	85 ✓	0 ✗	11	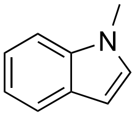	32 ✗	8 ✗	68
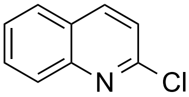	99 ✓	98 ✓	0	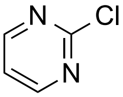	99 ✓	73 ✓	0
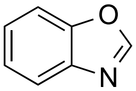	99 ✓	83 ✓	0	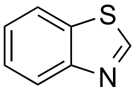	99 ✓	99 ✓	0
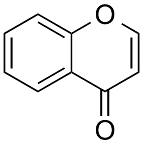	94 ✓	0 ✗	0	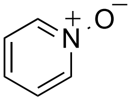	99 ✓	86 ✓	0
**Functional Groups**							
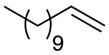	96 ✓	53 −	0	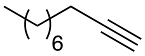	98 ✓	71 ✓	0
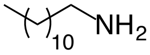	99 ✓	53 −	0	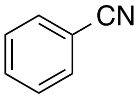	98 ✓	94 ✓	0
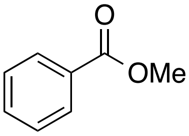	98 ✓	99 ✓	0	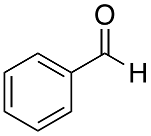	99 ✓	0 ✗	0
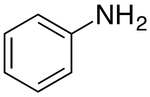	65 −	35 −	35	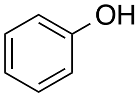	98 ✓	94 ✓	10
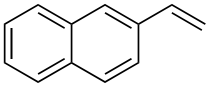	89 ✓	54 −	8				

✓ > 66% − 34-66% ✗ < 34%

EXPERIMENTAL

General Experimental Considerations

Reagent grade solvents were used without further purification or drying. Flash column chromatography was performed using 230-400 mesh silica with the indicated solvent system according to standard techniques. Analytical thin-layer chromatography (TLC) was performed on pre-coated, aluminium-backed silica gel plates. Visualization of the developed chromatogram was performed by UV absorbance (254 nm), aqueous potassium permanganate stain or iodine impregnated silica (generally selective for NH sulfoximines). Infrared spectra (ν_{max}, FTIR ATR) were recorded in reciprocal centimeters (cm^{-1}). Nuclear magnetic resonance spectra were recorded on 400 and 500 MHz spectrometers. Chemical shifts for ^1H NMR spectra are recorded in parts per million from tetramethylsilane with the solvent resonance as the internal standard (chloroform, δ = 7.26 ppm). Data is reported as follows: chemical shift [multiplicity (s = singlet, d = doublet, t = triplet, m = multiplet, app = apparent and br = broad), coupling constant in Hz, integration]. ^{13}C NMR spectra were recorded with complete proton decoupling. Chemical shifts are reported in parts per million from tetramethylsilane with the solvent resonance as the internal standard ($^{13}CDCl_3$: 77.0 ppm).

Reagents: Commercial reagents were used as supplied or purified by standard techniques where necessary.

General Procedure A for the preparation of NH Sulfoximines (MeOH)

The sulfoxide (0.30 mmol, 1.0 equiv.), $PhI(OAc)_2$ (290 mg, 0.90 mmol, 3.0 equiv.) and ammonium carbamate (94 mg, 1.20 mmol, 4.0 equiv.) were added to a 10 mL round bottom flask containing a stirrer bar. MeOH (0.6 mL, [0.5 M]) was added and the reaction was stirred for 30 min at 25 ºC in an open flask. The solvent was removed *in vacuo* and the crude residue was purified by flash column chromatography (SiO_2, dry loaded).

General Procedure B for the preparation of NH Sulfoximines (MeCN)

Sulfoxide (0.30 mmol, 1.0 equiv.) was dissolved in MeCN (0.6 mL, [0.5 M]) in a 10 mL round bottom flask containing a stirrer bar. Ammonium carbamate (59 mg, 0.75 mmol, 2.5 equiv.) was added followed by $PhI(OAc)_2$ (145 mg, 0.45 mmol, 3.0 equiv.) and the reaction was stirred for 3 h at 25 ºC in an open flask. CH_2Cl_2 (5 mL) and saturated aqueous $NaHCO_3$ solution (5 mL) were added and the mixture stirred for 10 min at rt. The reaction mixture was transferred to a separating funnel and the organic layer was separated. The aqueous layer was extracted with CH_2Cl_2 (2 × 5 mL), the organic layers were combined, dried over anhydrous Na_2SO_4, filtered and the solvent removed *in vacuo*. The crude residue was purified by flash column chromatography (SiO_2).

General Procedure C for the preparation of NH Sulfoximines (Toluene)

The sulfoxide (0.30 mmol, 1.0 equiv.), $PhI(OAc)_2$ (193 mg, 0.60 mmol, 2.0 equiv.), ammonium carbamate (47 mg, 0.60 mmol, 2.0 equiv.) and MgO (48 mg, 1.20 mmol, 4.0 equiv.) were added to a 10 mL round bottom flask containing a stirrer bar. Toluene (0.5 mL, [0.6 M]) was added and the reaction was stirred for 16 h at 35 ºC in an open flask. The reaction mixture was filtered through a plug of Celite and washed with CH_2Cl_2 (2 × 5 mL). The solvent was removed *in vacuo* and the crude residue was purified by flash column chromatography (SiO_2).

Imino(methyl)(*p*-tolyl)-λ[6]-sulfanone (1a)

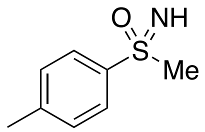

0.3 mmol scale
Prepared according to **General Procedure A** using *p*-tolylmethyl sulfoxide (47 mg, 0.3 mmol) and purified by flash column chromatography (SiO_2, dry loaded, EtOAc) to afford the title sulfoximine as an off-white solid, 49 mg (96%).
Prepared according to **General Procedure B** using *p*-tolylmethyl sulfoxide (47 mg, 0.3 mmol) and purified by flash column chromatography (SiO_2, EtOAc) to afford the title sulfoximine as an off-white solid,50 mg (98%). Also prepared according to **General Procedure C** using *p*-tolylmethyl sulfoxide (47 mg, 98%, 0.3 mmol) and purified by flash column chromatography (SiO_2, EtOAc) to afford the title sulfoximine as an off-white solid, 46 mg (90%).

10 mmol scale
Prepared according to **General Procedure A** using *p*-tolylmethyl sulfoxide (1.57 g, 98%, 10.0 mmol), $PhI(OAc)_2$ (9.66 g, 30.0 mmol, 3.0 equiv.) and ammonium carbamate (3.12 g, 40.0 mmol, 4.0 equiv.) in MeOH (20 mL, [0.5 M]). The crude residue was purified by flash column chromatography (SiO_2, dry loaded, EtOAc) to afford the title sulfoximine as an off-white solid, 1.53 g (91%).
R_f = 0.18 (EtOAc); m.p. = 69-70 ºC; IR (film) ν_{max} (cm^{-1}): 3273, 2924, 1597, 1408, 1216, 1093 cm^{-1}; ^1H NMR (400 MHz, $CDCl_3$, δ): 7.94 – 7.80 (m, 2H), 7.33 (d, *J* = 8.0 Hz, 2H), 3.08 (s, 3H), 2.43 (s, 4H, C*H*$_3$, N*H*); ^{13}C NMR (101 MHz, $CDCl_3$, δ): 144.0, 140.6, 130.0, 127.8, 46.4, 21.6; HRMS: (ESI-TOF) *m/z*: calcd for $C_8H_{12}NOS$ $[M+H]^+$ 170.0640, found 170.0640. Analytical data in agreement with those reported in the literature.[1]

Imino(methyl)(*p*-tolyl)-λ[6]-sulfanone ((*S*)-1a)
Chiralcel OD column, 85:15 (hexane/*i*-PrOH), 0.5 ml/min., er 97:3, (*S*)-isomer (major): 31.8 min, (*R*)-isomer (minor): 25.6 min.

(Benzyl)(imino)(phenyl)-λ[6]-sulfanone (1b)

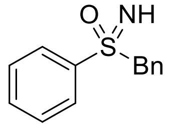

Prepared according to **General Procedure A** using phenyl benzyl sulfoxide (65 mg, 0.30 mmol) and purified by flash column chromatography (SiO_2, 30% Hexane/EtOAc) to afford the title sulfoximine as a white solid, 53 mg (76%).
R_f = 0.5 (30% hexane/EtOAc); mp= 107-109 ºC; IR ν_{max} (cm^{-1}): 3445, 1637, 1445, 1217, 1110, 977, 756, 700, 534; ^1H NMR (500 MHz, $CDCl_3$, δ): 7.76 (d, *J* = 7.8 Hz, 2H), 7.58 (t, *J* = 7.4 Hz, 1H), 7.45 (t, *J* = 7.8 Hz, 2H), 7.35-7.24 (m, 3H), 7.11 (d, *J* = 7.5 Hz, 2H), 4.41 and 4.33 (2 × d, AB system, *J* = 13.4 Hz, 2H); ^{13}C {^1H} NMR (126 MHz, $CDCl_3$, δ): 140.3 (qC), 133.3, 131.2 (2C), 129.0 (3C), 128.9 (2C), 128.6_3 (2C), 128.6_0, 64.7; HRMS (ESI-TOF) *m/z*: calcd for $C_{13}H_{13}NNaOS^+$ $[M+Na]^+$ 254.0610, found 254.0617.

(*R*)-(Benzyl)(imino)(phenyl)-λ[6]-sulfanone ((*R*)-1b)
$[\alpha]^{20}_D$ = + 28 (*c* 1.0, $CHCl_3$); Chiral HPLC: LUX1 column, 90:10 (hexane/*i*-PrOH), 0.5ml/min, er 98.5:1.5, (*R*)-isomer (major):50.2 min, (*S*)-isomer (minor):52.2 min.

(Fluoromethyl)(imino)(phenyl)-λ[6]-sulfanone (1c)

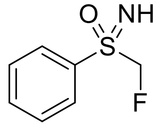

Prepared according to **General Procedure A** using phenyl-fluoromethyl sulfoxide (47 mg, 0.30 mmol) and purified by flash column chromatography (SiO_2, 40% EtOAc/hexane) to afford the title sulfoximine as a yellow oil, 36 mg (69%).
Prepared according to **General Procedure B** using phenyl-fluoromethyl sulfoxide (47 mg, 0.3 mmol) and purified by flash column chromatography (SiO2, 40% EtOAc/hexane) to afford the title sulfoximine as yellow oil, 41 mg (78%).
R_f = 0.4 (40% EtOAc/hexane); IR ν_{max} (cm^{-1}): 3274, 1447, 1246, 1057, 992, 747, 687, 539; ^1H NMR (500 MHz, $CDCl_3$, δ): 8.03 (d, *J* = 7.5 Hz, 2H), 7.69 (t, *J* = 7.5 Hz, 1H), 7.59 (t, *J* = 7.7 Hz, 2H), 5.16 (dd, *J* = 13.0, 9.7 Hz, 1H), 5.07 (dd, *J* = 13.0, 9.7 Hz 1H), 2.75 (br s, exchange with D_2O, N*H*); ^{13}C {^1H} NMR (126 MHz, $CDCl_3$, δ): 137.8 (qC), 134.3, 129.5 (2C), 129.3 (2C), 92.9 (d, *J* = 222.9, C*H*$_2$F); ^{19}F NMR (470 MHz, $CDCl_3$, δ): -27.19 (t, *J* = 47,0 Hz). HRMS (ESI-TOF) *m/z*: calcd for $C_7H_8FNNaOS^+$ $[M+Na]^+$ 196.0203, found 196.0193.

Imino(isopropyl)(*p*-tolyl)-λ^6-sulfanone (1d)

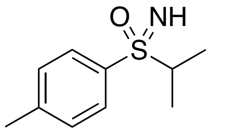

Prepared according to **General Procedure A** using tolyl-isopropyl sulfoxide (57 mg, 0.30 mmol) and purified by flash column chromatography (SiO_2, dry loaded, 60% EtOAc/pentane) to afford the title sulfoximine as a white solid, 52 mg (88%). R_f = 0.25 (60% EtOAc/pentane); mp = 60-62 ºC; IR (film) ν_{max}: 3262, 2969, 2927, 1592, 1464, 1448, 1387, 1369, 1263, 1208, 1103, 1045, 1017, 983, 813, 678 cm^{-1}; ^{1}H NMR (400 MHz, $CDCl_3$, δ): 7.81–7.75 (m, 2H), 7.31 (d, *J* = 8.1 Hz, 2H), 3.19 (hept, *J* = 6.8 Hz, 1H), 2.55 (br s, 1H), 2.41 (s, 3H), 1.28 (d, *J* = 6.8 Hz, 3H), 1.24 (d, *J* = 6.8 Hz, 3H); ^{13}C {^{1}H} NMR (101 MHz, $CDCl_3$, δ): 143.9 (qC), 136.8 (qC), 129.7 (2C), 127.5 (2C), 56.6, 21.6, 16.5, 16.1; HRMS (ESI-TOF) *m/z*: $[M+H]^+$ calcd for $C_{10}H_{16}NOS$ 198.0953, found 198.0959.

Imino(phenethyl)(*p*-tolyl)-λ^6-sulfanone (1e)

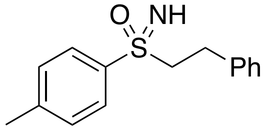

Prepared according to **General Procedure A** using tolyl-ethylphenyl sulfoxide (73 mg, 0.30 mmol) and purified by flash column chromatography (SiO_2, dry loaded, 50% EtOAc/pentane) to afford the title sulfoximine as a colourless oil, 65 mg (84%). R_f = 0.38 (50% EtOAc/pentane); IR (film) ν_{max}: 3267, 3030, 1597, 1496, 1455, 1404, 1217, 1114, 1090, 1024, 991, 816, 764, 736, 699 cm^{-1}; ^{1}H NMR (400 MHz, $CDCl_3$, δ): 7.89–7.83 (m, 2H), 7.34 (d, *J* = 8.0 Hz, 2H), 7.26–7.21 (m, 2H), 7.21–7.15 (m, 1H), 3.47–3.29 (m, 2H), 3.13–2.92 (m, 2H), 2.74 (br s, 1H), 2.44 (s, 3H); ^{13}C {^{1}H} NMR (101 MHz, $CDCl_3$, δ): 144.1 (qC), 138.8 (qC), 137.8 (qC), 130.0 (2C), 128.8 (2C), 128.5 (2C), 128.4 (2C), 126.8, 58.8, 29.4, 21.6; HRMS (ESI-TOF) *m/z*: $[M+H]^+$ calcd for $C_{15}H_{18}NOS$ 260.1109, found 260.1113.

(Chloromethyl)(imino)(*p*-tolyl)-λ^6-sulfanone (1f)

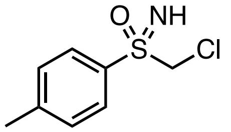

Prepared according to **General Procedure A** using cholormethyl-tolyl sulfoxide (57 mg, 0.30 mmol) and purified by flash column chromatography (SiO_2, dry loaded, 50% EtOAc/pentane) to afford the title sulfoximine as a white solid, 51 mg (84%). R_f = 0.41 (50% EtOAc/pentane); mp = 92-93 ºC; IR (film) ν_{max}: 3284, 3000, 2937, 1595, 1489, 1447, 1398, 1381, 1243, 1215, 1148, 1114, 1041, 979, 865, 812, 769, 713 cm^{-1}; ^{1}H NMR (400 MHz, $CDCl_3$, δ): 7.92–7.88 (m, 2H), 7.39–7.32 (m, 2H), 4.55 and 4.53 (2 × d, *AB* system, *J* = 12.5 Hz, 2H), 3.13 (s, 1H), 2.44 (br s, 1H); ^{13}C {^{1}H} NMR (101 MHz, $CDCl_3$, δ): 145.2 (qC), 134.6 (qC), 130.0 (2C), 129.5 (2C), 61.4, 21.7; HRMS (ESI-TOF) *m/z*: $[M+H]^+$ calcd for $C_8H_{11}NOSCl$ 204.0250, found 204.0244.

(4-Chlorophenyl)(ethyl)(imino)-λ^6-sulfanone (1g)

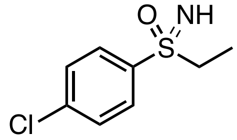

Prepared according to **General Procedure A** using *p*-chlorophenyl-ethyl sulfoxide (57 mg, 0.30 mmol) and purified by flash column chromatography (SiO_2, dry loaded, 70 % EtOAc/pentane) to afford the title sulfoximine as a colourless oil, 53 mg (87%). R_f = 0.12 (70% EtOAc/pentane); IR (film) ν_{max}: 3270, 3087, 2981, 2938, 1643, 1580, 1472, 1393, 1278, 1211, 1084, 1053, 972, 829, 777, 749, 712, 686 cm^{-1}; ^{1}H NMR (400 MHz, $CDCl_3$, δ): 7.91–7.83 (m, 2H), 7.52–7.45 (m, 2H), 3.22–3.04 (m, 2H), 2.67 (s, 1H), 1.22 (t, *J* = 7.4 Hz, 3H); ^{13}C {^{1}H} NMR (101 MHz, $CDCl_3$, δ): 140.0 (qC), 139.8, 130.1 (2C), 129.5 (2C), 52.0, 7.9; HRMS (ESI-TOF) *m/z*: $[M+H]^+$ calcd for $C_8H_{11}NOSCl$ 204.0250, found 204.0255.

(Benzyl)(4-bromophenyl)(imino)-λ^6-sulfanone (1h)

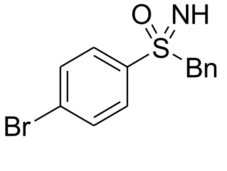

Prepared according to **General Procedure A** using (*p*-bromophenyl)benzyl sulfoxide (82 mg, 0.30 mmol) and purified by flash column chromatography (SiO_2, 50% EtOAc/hexane) to afford the title sulfoximine as a white solid, 69 mg (74%).

Prepared according to **General Procedure B** using (*p*-bromophenyl)benzyl sulfoxide (82 mg, 0.3 mmol) and purified by flash column chromatography (SiO2, 50% EtOAc/hexane) to afford the title sulfoximine as a white solid, 68 mg (73%).

R_f = 0.5 (50% EtOAc/hexane); mp= 130-131 ºC; IR ν_{max} (cm^{-1}): 3436, 1570, 1492, 1453, 1385, 1227, 1123, 1066, 991, 813, 770, 736, 698; ^1H NMR (500 MHz, $CDCl_3$, δ): 7.62-7.55 (m, 4H), 7.38-7.26 (m, 3H), 7.11 (d, *J* = 7.2 Hz, 2H), 4.38 and 4.30 (2 × d, AB system, *J* = 13.5 Hz, 2H); ^{13}C {^1H} NMR (126 MHz, $CDCl_3$, δ): 139.5 (qC), 132.2 (2C), 131.2 (2C), 130.6 (2C), 129.1, 128.7 (2C), 128.6, 128.4 (qC), 64.7; HRMS (ESI-TOF) *m/z*: calcd for $C_{13}H_{12}BrNNaOS^+$ $[M+Na]^+$ 331.9715, found 331.9706.

(*R*)- (Benzyl)(4-bromophenyl)(imino)-λ^6-sulfanone

$[\alpha]^{20}_D$ = + 28 (*c* 1.0, $CHCl_3$); Chiral HPLC: LUX1 column, 90:10 (Hexane:*i*-PrOH), 1ml/min, er 99.9:0.1%, (*R*)-isomer (major):29.114 min, (*S*)-isomer (minor):33.906 min.

(1-Chloroethyl)(4-chlorophenyl)(imino)-λ^6-sulfanone (1i)

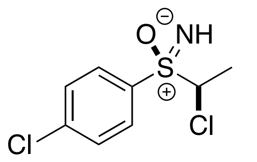

Prepared according to **General Procedure A** using *p*-chlorophenyl-chloroethyl sulfoxide[2] (67 mg, 0.30 mmol, as a mixture of diastereomers, 9:1) and purified by flash column chromatography (SiO_2, dry loaded, 30% EtOAc/pentane) to afford the title sulfoximine as a colourless oil, 63 mg (85%, mixture of diastereomers, 9:1). R_f = 0.29 (minor) and 0.24 (major, 30% EtOAc/pentane); IR (film) ν_{max}: 3267, 3090, 2936, 1576, 1472, 1444, 1394, 1239, 1130, 1089, 967, 829, 755, 703, 663 cm^{-1}; ^1H NMR (400 MHz, $CDCl_3$, δ, major diastereomer): 7.99–7.93 (m, 2H), 7.57–7.52 (m, 2H), 4.85 (q, *J* = 6.7 Hz, 1H), 3.06 (br s, 1H), 1.78 (d, *J* = 6.7 Hz, 3H); ^{13}C {^1H} NMR (101 MHz, $CDCl_3$, δ, major diastereomer): 140.8 (qC), 135.2 (qC), 131.6 (2C), 129.4 (2C), 72.6, 19.0; HRMS (ESI-TOF) *m/z*: $[M+H]^+$ calcd for $C_8H_{10}NOS$ 237.9860, found 237.9865.

(4-Acetylphenyl)(imino)(methyl)-λ^6-sulfanone (1j)

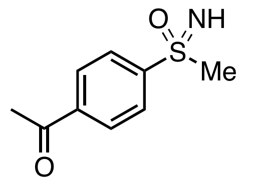

Prepared according to **General Procedure A** using *p*-acetophenyl-methyl sulfoxide (55 mg, 0.30 mmol) and purified by flash column chromatography (SiO_2, dry loaded, EtOAc) to afford the title sulfoximine as an off-white solid, 38 mg (64%). R_f = 0.22 (EtOAc); mp = 92-94 ºC; IR (film) ν_{max}: 3278, 2996, 2919, 1747, 1678, 1574, 1592, 1392, 1354, 1327, 1264, 1218, 1132, 1097, 1018, 949, 841, 823, 773, 743 cm^{-1}; ^1H NMR (400 MHz, $CDCl_3$, δ): 8.08 (s, 4H), 3.10 (s, 3H), 2.81 (br s, 1H), 2.63 (s, 3H); ^{13}C {^1H} NMR (101 MHz, $CDCl_3$, δ): 196.9 (qC), 147.5 (qC), 140.5 (qC), 129.2 (2C), 128.2 (2C), 46.1, 27.1; HRMS (ESI-TOF) *m/z*: $[M+H]^+$ calcd for $C_9H_{12}NO_2S$ 198.0589, found 198.0591.

(4-Bromophenyl)(imino)(napht-2-yl)-λ^6-sulfanone (1k)

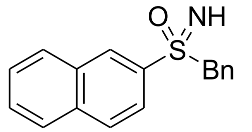

Prepared according to **General Procedure B** using (2-napthyl)benzyl sulfoxide (80mg, 0.30 mmol) and purified by flash column chromatography (SiO_2, 50% EtOAc/hexane) to afford the title sulfoximine as a white solid, 76 mg (90%). R_f = 0.3 (50% EtOAc/hexane); mp= 179-181 ºC; IR ν_{max} (cm^{-1}): 3244, 2912, 1230, 1141, 1099, 1067, 1033, 827, 766, 747, 696; ^1H NMR (500 MHz, $CDCl_3$, δ): 8.35 (s, 1H), 7.91 (t, *J* = 7.5 Hz, 3H), 7.76 (dd, J = 8.7, 1.9 Hz, 1H), 7.70-7.65 (m, 1H), 7.64-7.59 (m, 1H), 7.33 (t, *J* = 7.4 Hz, 1H), 7.26-722 (m, 2H), 7.14 (d, *J* = 7.2 Hz, 2H), 4.63 and 4.55 (2 × d, AB system, *J* = 13.6 Hz, 2H); ^{13}C {^1H} NMR (126 MHz, $CDCl_3$, δ): 136.9 (qC), 135.2, 132.3 (qC), 131.3 (2C), 130.8 (qC), 129.5, 129.2 (2C), 129.0, 128.7 (2C), 128.4 (qC), 128.0, 127.6, 123.8, 64.6; HRMS (ESI-TOF) *m/z*: calcd for $C_{17}H_{15}NNaOS^+$ $[M+Na]^+$ 304.0767, found 304.0774.

Imino(phenyl)(vinyl)-λ^6-sulfanone (1l)

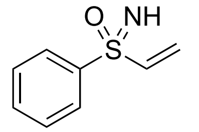

Prepared according to **General Procedure A** using phenyl-vinyl sulfoxide (46 mg, 0.30 mmol) and purified by flash column chromatography (SiO_2, dry loaded, 50 % EtOAc/pentane) to afford the title sulfoximine as a pale yellow oil, 27 mg (54%). R_f = 0.13 (50% EtOAc/pentane); IR (film) ν_{max}: 3265, 3062, 1639, 1583, 1476, 1446, 1255, 1218, 1128, 1095, 1070, 972, 760, 731, 691 cm^{-1}; ^1H NMR (400 MHz, $CDCl_3$, δ): 8.01–7.92 (m, 2H), 7.64–7.54 (m, 1H), 7.56–7.46 (m, 2H), 6.73 (dd, *J* = 16.4, 9.5 Hz, 1H), 6.40 (d, *J* = 16.4 Hz, 1H), 5.97 (d, *J* = 9.5 Hz, 1H), 2.88 (br s,

1H); ^{13}C {^{1}H} NMR (101 MHz, $CDCl_3$, δ): 141.9 (qC), 140.6, 133.1, 129.3 (2C), 128.2 (2C), 126.7; HRMS (ESI-TOF) *m/z*: $[M+H]^+$ calcd for $C_8H_{10}NOS$ 168.0483, found 168.0480.

Iminodiphenyl-λ^6-sulfanone (1m)

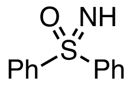

Prepared according to **General Procedure A** using diphenyl sulfoxide (61 mg, 0.30 mmol) and purified by flash column chromatography (SiO_2, dry loaded, EtOAc) to afford the title sulfoximine as an off-white solid, 58 mg (89%).

R_f = 0.44 (60% EtOAc/pentane); mp = 103-104 ºC; IR (film) ν_{max}: 3267, 3062, 1581, 1313, 1475, 1446, 1313, 1222, 1127, 1094, 1065, 956 cm^{-1}; ^{1}H NMR (400 MHz, $CDCl_3$, δ): 8.09–7.96 (m, 4H), 7.54–7.40 (m, 6H), 3.08 (br s, 1H); ^{13}C {^{1}H} NMR (101 MHz, $CDCl_3$, δ): 143.4 (2 × qC), 132.65 (2C), 129.21 (4C), 127.98 (4C); HRMS (ESI-TOF) *m/z*: $[M+H]^+$ calcd for $C_{12}H_{12}NOS$ 218.0640, found 218.0639.

Dibenzyl(imino)-λ^6-sulfanone (1n)

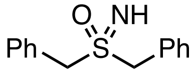

Prepared according to **General Procedure A** using dibenzyl sulfoxide (69 mg, 0.30 mmol) and purified by flash column chromatography (SiO_2, dry loaded, 50% EtOAc/pentane) to afford the title sulfoximine as a white solid, 63 mg (86%). R_f = 0.24 (50% EtOAc/pentane); mp = 173-174 ºC; IR (film) ν_{max}: 3246, 3064, 3030, 2978, 2919, 1490, 1454, 1418, 1257, 1247, 1156, 1150, 1072, 1026, 759, 696 cm^{-1}; ^{1}H NMR (400 MHz, $CDCl_3$, δ): 7.41 (s, 10H), 4.27 (d, *J* = 13.1 Hz, 2H), 4.17 (d, *J* = 13.1 Hz, 2H), 2.53 (br s, 1H); ^{13}C {^{1}H} NMR (101 MHz, $CDCl_3$, δ): 131.2 (4C), 129.1 (2C), 129.0 (4C), 127.9 (2 × qC), 60.6 (2C); HRMS (ESI-TOF) *m/z*: $[M+H]^+$ calcd for $C_{14}H_{16}NOS$ 246.0953, found 246.0958.

***tert*-Butyl(imino)(methyl)-λ^6-sulfanone (1o)**

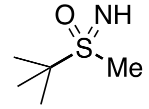

Prepared according to **General Procedure A** using *tert*-butyl-methyl sulfoxide (33 mg, 0.30 mmol) and purified by flash column chromatography (SiO_2, dry loaded, 30% acetone/EtOAc) to afford the title sulfoximine as a colourless, 29 mg (79%). R_f = 0.15 (30% acetone/EtOAc); IR (film) ν_{max}: 3296, 2988, 2929, 2878, 1481, 1465, 1414, 1372, 1323, 1189, 1082, 1026, 990, 932, 808, 749 cm^{-1}; ^{1}H NMR (400 MHz, $CDCl_3$, δ): 2.87 (s, 3H), 1.95 (s, 1H), 1.44 (s, 9H); ^{13}C {^{1}H} NMR (101 MHz, $CDCl_3$, δ): 60.3 (qC), 36.0, 23.9 (3C); HRMS (CI) *m/z*: $[M+H]^+$ calcd for $C_2H_{10}NOS$ 120.0483, found 120.0475.

1-Iminotetrahydro-1*H*-1λ^6-thiophene 1-oxide (1p)

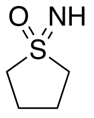

Prepared according to **General Procedure A** using tetrahydrothiophene 1-oxide (31 mg, 0.30 mmol) and purified by flash column chromatography (SiO_2, dry loaded, 10% MeOH/CH_2Cl_2) to afford the title sulfoximine as a pale yellow oil, 30 mg (84%). R_f = 0.36 (10% MeOH/CH_2Cl_2); IR (film) ν_{max}: 3401, 3259, 2953, 1650, 1449, 1416, 1275, 1192, 1140, 1077, 992, 895, 718, 664 cm^{-1}; ^{1}H NMR (400 MHz, $CDCl_3$, δ): 3.13–3.02 (m, 4H), 2.55 (s, 1H), 2.27–2.13 (m, 4H); ^{13}C {^{1}H} NMR (101 MHz, $CDCl_3$, δ): 55.4 (2C), 25.0 (2C); HRMS (ESI-TOF) *m/z*: $[M+H]^+$ calcd for $C_4H_{10}NOS$ 120.0483, found 120.0476.

1-imino-1λ^6-thietane 1-oxide (1q)

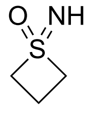

Prepared according to **General Procedure A** using thietane sulfoxide[3] (23 mg, 0.25 mmol) and purified by flash column chromatography (SiO_2, 5% MeOH/CH_2Cl_2) to afford the title sulfoximine as a colourless oil, 24 mg (90%).

Prepared according to **General Procedure B** using the thietane sulfoxide (1.0 g, 11 mmol) and purified by flash column chromatography (SiO_2, 5% MeOH/CH_2Cl_2) to afford the title sulfoximine as a colourless oil, 937 mg (81%). R_f = 0.4 (5% MeOH/CH_2Cl_2); IR (film) ν_{max}: 3257, 1652, 1401, 1231, 1205, 1167, 1016; ^{1}H NMR (400 MHz, $CDCl_3$, δ): 4.12-3.96 (m, 4H), 3.10 (br s, *N*H), 2.26-2.12 (m, 2H); ^{13}C {^{1}H} NMR (101 MHz, $CDCl_3$, δ): 66.6 (2C), 7.6; HRMS (ESI-TOF) *m/z*: $[M+H]^+$ calcd for C_3H_7NOS 105.0248, found 105.0254.

(1*R,2*R**)-2-Benzyl-1-iminothietane 1-oxide ((±)1r)**

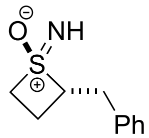

Prepared according to **General Procedure C** using the corresponding sulfoxide[3] (54 mg, 0.30 mmol) and purified by flash column chromatography (SiO_2, EtOAc) to afford the title sulfoximine as a yellow oil, 49 mg (83%). R_f = 0.3 (EtOAc); IR ν_{max} (cm^{-1}) 3247, 2923, 1720, 1496, 1454, 1239, 1029, 751, 701; ^{1}H NMR (500 MHz, $CDCl_3$, δ): 7.32 (t, *J* = 7.3 Hz, 2H), 7.26-7.21 (m, 3H), 4.63-4.54 (m, 1H), 3.96-3.84 (m, 2H), 3.36 (dd, *J* = 14.7, 7.7 Hz, 1H), 3.05 (dd, *J* = 14.7, 7.7 Hz, 1H), 2.32-2.24 (m, 1H), 1.96-1.87 (m, 1H); ^{13}C {^{1}H} NMR (126 MHz, $CDCl_3$, δ): 136.6 (qC), 129.0 (2C), 128.7 (2C), 127.1, 78.5, 62.0, 35.9, 15.9; HRMS (ESI-TOF) *m/z*: calcd for $C_{10}H_{14}NOS^+$ $[M+H]^+$ 196.0791, found 196.0790.

(1*R,2*S**)-2-(Hydroxydiphenylmethyl)-1-iminothietane 1-oxide ((±)1s)**

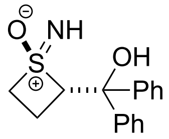

Prepared according to **General Procedure A** using the corresponding sulfoxide[3] (82 mg, 0.30 mmol) and purified by flash column chromatography (SiO_2, 40% EtOAc/hexane) to afford the title sulfoximine as a white solid, 71 mg (83%).
Prepared according to **General Procedure B** using the corresponding sulfoxide[4] (82 mg, 0.3 mmol) and purified by flash column chromatography (SiO2, 40% EtOAc/hexane) to afford the title sulfoximine as a white solid, 54 mg (63%).
Also prepared according to **General Procedure C** using sulfoxide[4] (82 mg, 0.3 mmol) and purified by flash column chromatography (SiO2, 40% EtOAc/hexane) to afford the title sulfoximine as a white solid, 26 mg (30%). R_f = 0.5 (40% EtOAc/hexane); mp= 152-154 ºC; IR ν_{max} (cm^{-1}): 3304, 1449, 1246, 1191, 1066, 700; ^{1}H NMR (500 MHz, $CDCl_3$, δ): 8.11 (s, exchange with D_2O 1H), 7.61–7.58 (m, 2H), 7.41–7.33 (m, 4H), 7.31–7.27 (m, 3H), 7.23–7.18 (m, 1H), 5.32 (app t, *J* = 9.2 Hz, 1H), 4.69 (s, exchange with D_2O, 1H), 4.05–3.89 (m, 2H), 2.57 (app tt, *J* = 11.6, 8.6 Hz, 1H), 2.04-1.90 (m, 1H); ^{13}C {^{1}H} NMR (126 MHz, $CDCl_3$, δ): 145.6 (qC), 143.7 (qC), 128.8 (2C), 128.5 (2C), 128.0, 127.4, 126.2 (2C), 125.6 (2C), 83.1 , 77.3 (qC), 63.4, 10.5; HRMS (ESI-TOF) *m/z*: calcd for $C_{16}H_{17}NNaO_2S^+$ $[M+Na]^+$ 310.0872; found 310.0871.

(1*R,2*R**)-2-(Hydroxydiphenylmethyl)-1-iminothietane 1-oxide ((±)1t)**

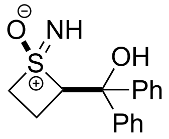

Prepared according to **General Procedure B** using the corresponding sulfoxide[4] (82 mg, 0.30 mmol) and purified by flash column chromatography (SiO_2, 50% EtOAc/hexane) to afford the title sulfoximine as a white solid, 53 mg (62%). R_f = 0.5 (50% EtOAc/hexane); mp= 130-132 ºC; IR ν_{max} (cm^{-1}): 3473, 3274, 2921, 1722, 1447, 1261, 1224, 1188, 1096, 1030, 777, 701; ^{1}H NMR (500 MHz, $CDCl_3$, δ): 7.60 (d, *J* = 7.8 Hz, 2H), 7.40-7.33 (m, 4H), 7.32-7.26 (m, 3H), 7.20 (t, *J* = 7.2 Hz, 1H), 5.33 (*like* t, *J* = 9.3 Hz, 1H), 4.7 (s, exchange with D2O, 1H), 4.07 (dt, *J* = 13.1, 9.6 Hz, 1H), 3.93-3.85 (m, 1H), 2.57 (m, 1H), 2.07-1.97 (m, 1H). ^{13}C {^{1}H} NMR (126 MHz, $CDCl_3$, δ): 145.4 (qC), 143.4 (qC), 128.9 (2C), 128.6 (2C), 128.1, 127.4, 126.0 (2C), 125.5 (2C), 86.0, 77.9 (qC), 62.0, 10.8; HRMS (ESI-TOF) *m/z*: calcd for $C_{16}H_{17}NNaO_2S^+$ $[M+Na]^+$ 310.0872; found 310.0871.

(2*R,4*R**)-2,4-bis(2-Hydroxypropan-2-yl)-1-iminothietane 1-oxide ((±)1u)**

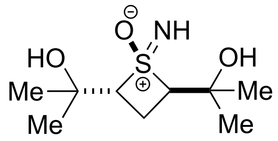

Prepared according to **General Procedure B** using the corresponding sulfoxide sulfoxide**[4]** (62 mg, 0.30 mmol) and purified by flash column chromatography (SiO_2, EtOAc) to afford the title sulfoximine as a white solid, 54 mg (82%). R_f = 0.5 (EtOAc); mp= 92-94 ºC; IR ν_{max} (cm^{-1}): 3430, 2974, 1644, 1384, 1224, 1200; ^{1}H NMR (500 MHz, $CDCl_3$, δ): 4.09 (ddd, *J* = 10.9, 8.0, 1.0 Hz, 1H), 4.04 (ddd, *J* = 10.7, 5.7, 0.9 Hz, 1H), 2.44 (td, *J* = 11.3, 8.0 Hz, 1H), 2.32 (td, *J* = 11.4, 5.7 Hz, 1H), 1.52 (s, 6H), 1.19 (s, 3H), 1.14 (s, 3H); ^{13}C {^{1}H} NMR (126 MHz, $CDCl_3$, δ): 86.3, 84.1, 70.6 (qC), 69.7 (qC), 29.3, 29.2, 27.3, 27.0, 14.0; HRMS (ESI-TOF) *m/z*: calcd for $C_9H_{19}NNaO_3S^+$ $[M+Na]^+$ 244.0978, 244.0979.

(Benzo[d]thiazol-2-yl)(benzyl)(imino)-λ^6-sulfanone (1v)

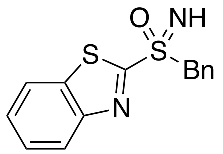

Prepared according to **General Procedure A** using 2-(benzylsulfinyl)benzo[d]thiazole[4] (82 mg, 0.30 mmol) and purified by flash column chromatography (SiO_2, 30% EtOAc/hexane) to afford the title sulfoximine as a white solid, 78 mg (90%). R_f = 0.5 (30% EtOAc/hexane); m.p. = 138-140; IR ν_{max} (cm^{-1}): 3272, 2917, 1719, 1469,1455, 1240, 1106,1021, 764, 730, 696 ^{1}H NMR (500 MHz, $CDCl_3$ δ): 8.23 (d, *J* = 8.3 Hz, 1H), 7.92 (d, *J* = 8.1 Hz, 1H), 7.62 (t, *J* = 7.7 Hz, 1H), 7.55 (t, *J* = 7.7 Hz, 1H), 7.36-7.25 (m, 5H), 4.84 (d, *J* = 13.6 Hz, 1H), 4.67 (d, *J* = 13.5 Hz, 1H); ^{13}C {^{1}H} NMR (126 MHz, $CDCl_3$, δ): 153.0 (qC), 137.9 (qC) 131.4, 129.3, 128.9, 129.9 (qC), 127.7, 127.5, 127.1 (qC), 125.4, 122.3, 62.8; HRMS (ESI-TOF) *m/z*: calcd for $C_{14}H_{13}N_2OS_2^+$ $[M+H]^+$ 289.0464; found 289.0463.

***tert*-Butyl (2*S*)-2-((*tert*-butoxycarbonyl)amino)-4-(*S*-methylsulfonimidoyl)butanoate (1w)**

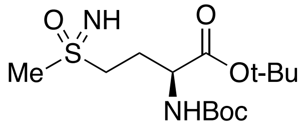

Prepared according to **General Procedure A** using L-methionine sulfoxide (96 mg, 0.30 mmol) and purified by flash column chromatography (SiO_2, dry loaded, EtOAc → 20% acetone/EtOAc) to afford the title sulfoximine as a white solid, 79 mg (78%). ^{1}H NMR (400 MHz, $CDCl_3$, δ): 5.38–4.24 (m, 1H), 3.26–3.13 (m, 1H), 3.14–3.03 (m, 1H), 2.97 (s, 3H), 2.79–2.60 (m, 1H), 2.43–2.29 (m, 1H), 2.19–2.02 (m, 1H), 1.45 (s, 9H), 1.42 (s, 9H); ^{13}C {^{1}H} NMR (101 MHz, $CDCl_3$, δ): 170.5, 155.5, 83.0, 80.3, 53.6, 52.7 43.2, 28.4, 28.1, 26.8. All other analytical data in agreement with those reported in the literature.[5]

(4-Bromophenyl)(imino)(trifluoromethyl)-λ^6-sulfanone (1x)

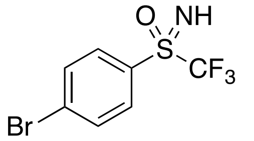

Prepared according to **General Procedure A** using *p*-bromophenyl-trifluoromethyl sulfoxide (82 mg, 0.30 mmol) and purified by flash column chromatography (SiO_2, dry loaded, 10% Et_2O/pentane) to afford the title sulfoximine as a white solid, 8 mg (9%). R_f = 0.29 (10% Et_2O/pentane); mp = 92-93 ºC; IR (film) ν_{max}: 3271, 1574, 1392, 1271, 1195, 1175,1069, 1101, 948, 819, 753, 737 cm^{-1}; ^{1}H NMR (400 MHz, $CDCl_3$, δ): 8.04-7.96 (m, 2H), 7.82-7.75 (m, 2H), 3.63 (br s, 1H); ^{13}C {^{1}H} NMR (101 MHz, $CDCl_3$, δ): 133.1 (2C), 132.1 (2C), 131.7 (qC), 130.7 (qC), 120.9 (q, *J* = 332 Hz); ^{19}F NMR (377 MHz, $CDCl_3$, δ): –78.7; HRMS (APCI) *m/z*: $[M+H]^+$ calcd for $C_7H_6NOSBrF_3$ 287.9300, found 287.9299.

Oxfendazole sulfoximine (1y)

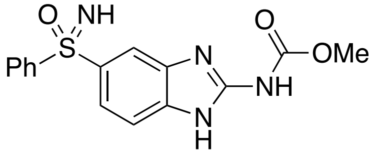

Prepared according to **General Procedure A*** using oxfendazole (31 mg, 0.1 mmol) and purified by flash column chromatography (SiO_2, EtOAc) to afford the title sulfoximine as a white solid, 23 mg (70%). R_f = 0.3 (EtOAc); m.p. = 272 °C dec.; IR ν_{max} (cm^{-1}): 3415, 2923, 1645, 1446, 1265, 1098. ^{1}H NMR (500 MHz, $CDCl_3$ δ): 3.85 (s, 3H), 7.50–7.60 (m, 4H), 7.81 (dd, *J* = 8.3, 2.2 Hz, 1H), 8.03 (m, 2H), 8.13 (br s, 1H); ^{13}C {^{1}H} NMR (126 MHz, $CDCl_3$, δ): 155.3 (qC), 143.7 (qC), 132.3, 130.9, 129.1, 129.9, 128.9 (2C), 127.3 (2C), 124.3, 52.1; HRMS (ESI-TOF) *m/z*: calcd for $C_{15}H_{15}N_4O_3S^+$ $[M+H]^+$ 331.0859; found 331.0859.

* Because of the very low solubility of the oxfendazole, a modification of General Procedure A was needed. In this instance the reaction was run on 0.1 mmol scale using MeOH as the solvent (2 mL), 8 equivalents of ammonium carbamate, 4 equivalents of $PhI(OAc)_2$ and the reaction was run for 24 h.

General Procedure D: Preparation of sulfoxides.

meta-Chloroperbenzoic acid (1.1 mmol, 1.1 equiv.) was addition portion-wise to a stirred solution of the sulfide (1.0 mmol, 1 equiv.) in CH_2Cl_2 (3 mL, 0.3 M) at 0 ºC. The reaction was stirred at 0 ºC for 1 h, quenched with saturated aqueous $NaHCO_3$ solution (3 mL) and extracted with CH_2Cl_2 (2 × 5 mL). The combined organic layers were dried over anhydrous Na_2SO_4, filtered and the solvent removed *in vacuo*. The crude residue was purified by flash column chromatography (SiO_2).

2-Methyl-2-(methylsulfinyl)propane

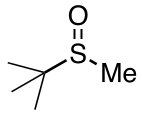

Prepared according to **General Procedure D** using *tert*-butyl-methyl sulfide (104 mg, 1.0 mmol) and purified by flash column chromatography (SiO_2, 5% MeOH/CH_2Cl_2) to afford the title sulfoxide as a colourless oil, 85 mg (71%). R_f = 0.21 (5% MeOH/CH_2Cl_2); ^{1}H NMR (400 MHz, $CDCl_3$, δ): 2.36 (s, 3H), 1.23 (s, 9H). All other analytical data in agreement with those reported in the literature.[6]

1-Bromo-4-((trifluoromethyl)sulfinyl)benzene

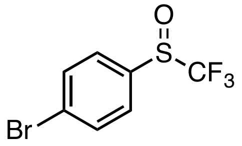

Prepared according to **General Procedure D** using *p*-bromophenyl-trifluoromethyl sulfide (257 mg, 1.0 mmol) and purified by flash column chromatography (SiO_2, 5 % Et_2O/pentane) to afford the title sulfoxide as colourless oil, 141 mg (52%). R_f = 0.37 (5% Et_2O/pentane); IR (film) ν_{max}: 3082, 1571, 1471, 1389, 1173, 1131, 1081, 1059, 1009, 950, 815, 745, 72, 698 cm^{-1}; ^{1}H NMR (400 MHz, $CDCl_3$, δ): 7.93–7.87 (m, 2H), 7.86–7.81 (m, 2H); ^{13}C {^{1}H} NMR (101 MHz, $CDCl_3$, δ): 134.7, 133.0, 128.7, 127.4, 124.4 (q, *J* = 335.4 Hz); ^{19}F NMR (377 MHz, $CDCl_3$, δ): –74.4; HRMS (APCI) *m/z*: $[M + H]^+$ calcd for $C_7H_5OSBrF_3$ 272.9191, found 272.9187.

1-(4-(Methylsulfinyl)phenyl)ethan-1-one

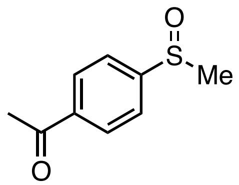

Prepared according to **General Procedure D** using *p*-acetophenyl-methyl sulfide (166 mg, 1.0 mmol) and purified by flash column chromatography (SiO_2, 60% EtOAc/pentane) to afford the title sulfoxide as a white solid, 168 mg (92%). R_f = 0.15 (60% EtOAc/pentane); ^{1}H NMR (400 MHz, $CDCl_3$, δ): 8.13–8.08 (m, 2H), 7.77–7.72 (m, 2H), 2.76 (s, 3H), 2.65 (s, 3H). All other analytical data in agreement with those reported in the literature.[7]

HPLC Analysis

The stereospecificity of this reaction was examined by using the enantioenriched sulfoxides (S)-*p*-tolylmethyl sulfoxide, (*R*)-*p*-bromophenyl-benzyl sulfoxide[8] and (*R*)-phenyl-benzyl sulfoxide.[9] In all cases the er of the starting sulfoxide was retained in the product sulfoximine

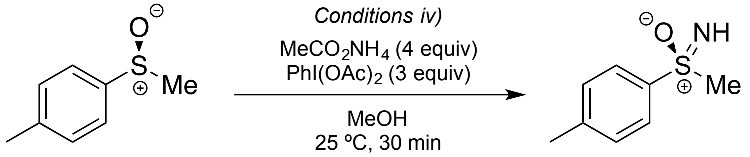

97:3 er

(*S*)-1a
97:3 er

Chiralcel OD column, 93:7 (Hexane:*i*-PrOH), 1.0 ml/min.

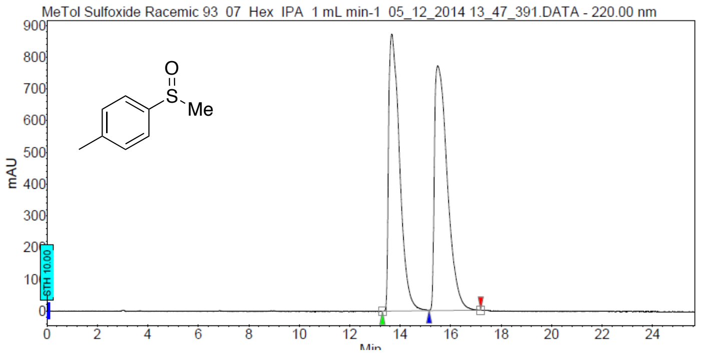

Peak results :

Index	Name	Time [Min]	Quantity [% Area]	Height [mAU]	Area [mAU.Min]	Area % [%]
1	UNKNOWN	13.666	48.79	872.1	436.5	48.793
2	UNKNOWN	15.479	51.21	772.6	458.1	51.207
Total			100.00	1644.6	894.6	100.000

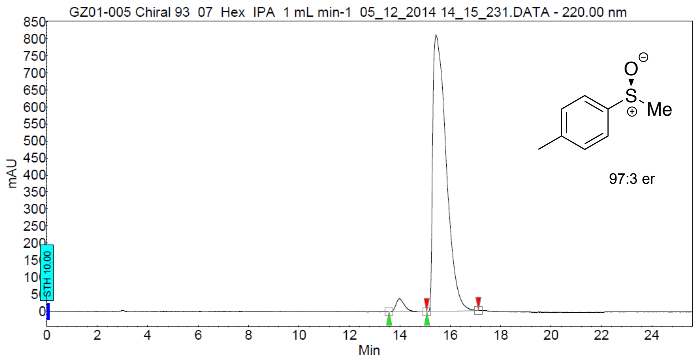

Peak results :

Index	Name	Time [Min]	Quantity [% Area]	Height [mAU]	Area [mAU.Min]	Area % [%]
1	UNKNOWN	13.986	2.95	37.8	15.2	2.947
2	UNKNOWN	15.426	97.05	814.0	500.4	97.053
Total			100.00	851.7	515.5	100.000

Chiralcel OD column, 85:15 (Hexane:*i*-PrOH), 0.5 ml/min.

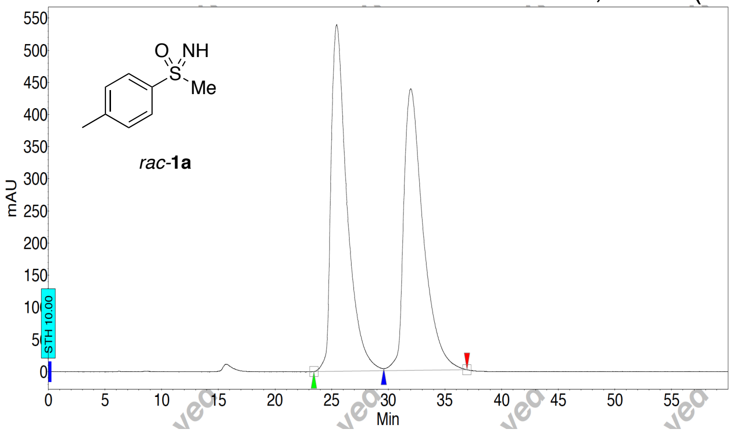

Peak results :

Index	Name	Time [Min]	Quantity [% Area]	Height [mAU]	Area [mAU.Min]	Area % [%]
1	UNKNOWN	25.451	50.30	539.8	885.9	50.303
2	UNKNOWN	31.984	49.70	438.1	875.2	49.697
Total			100.00	977.9	1761.2	100.000

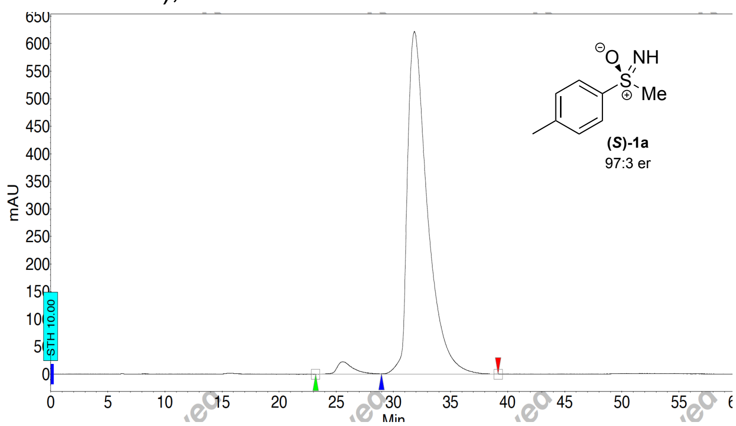

Peak results :

Index	Name	Time [Min]	Quantity [% Area]	Height [mAU]	Area [mAU.Min]	Area % [%]
1	UNKNOWN	25.571	2.84	22.7	36.9	2.843
2	UNKNOWN	31.824	97.16	622.8	1261.5	97.157
Total			100.00	645.5	1298.4	100.000

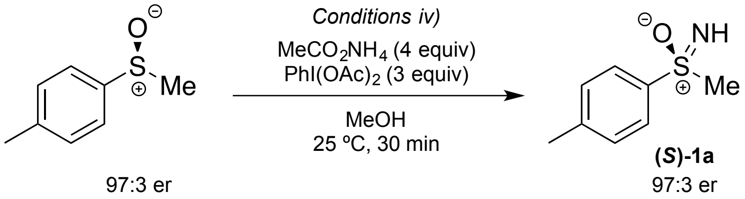

Chiralcel OD column, 85:15 (Hexane:*i*-PrOH), 0.5 ml/min.

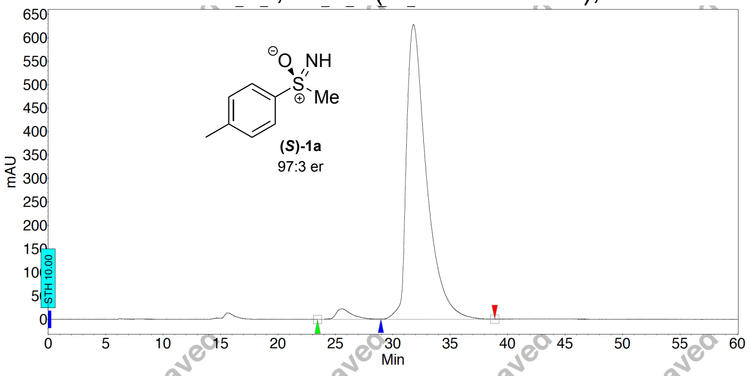

Peak results :

Index	Name	Time [Min]	Quantity [% Area]	Height [mAU]	Area [mAU.Min]	Area % [%]
1	UNKNOWN	25.571	2.79	22.6	36.4	2.786
2	UNKNOWN	31.824	97.21	628.8	1270.8	97.214
Total			100.00	651.4	1307.2	100.000

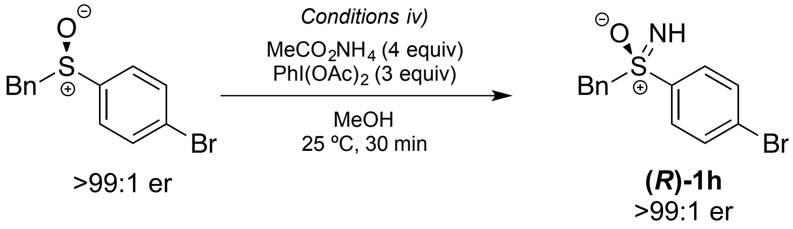

Lux1, Hex:iPrOH (90:10), 1 ml/min

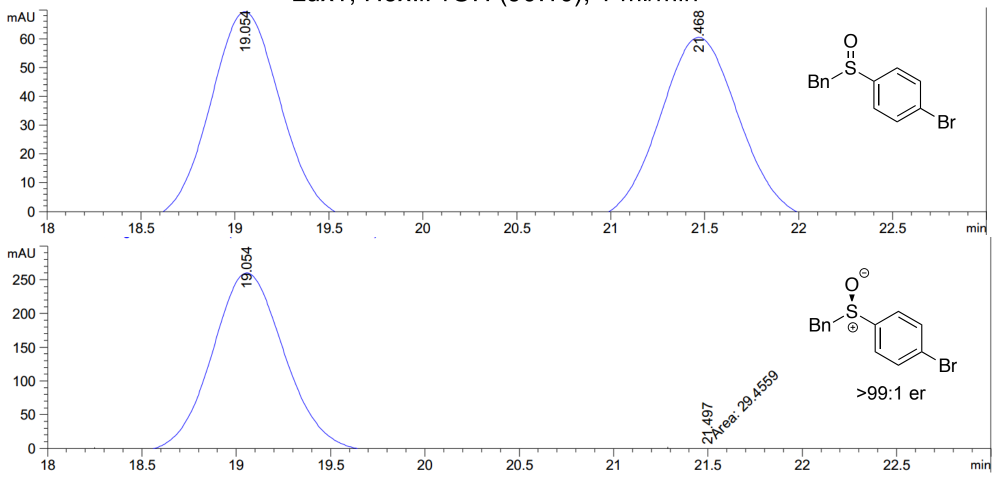

Peak #	RetTime [min]	Type	Width [min]	Area [mAU*s]	Height [mAU]	Area %
1	19.054	BB	0.4205	7277.77002	266.57724	99.5969
2	21.497	MM	0.1871	29.45590	2.62416	0.4031
Totals :				7307.22592	269.20140	

Lux1, Hex:iPrOH (90:10), 1 ml/min

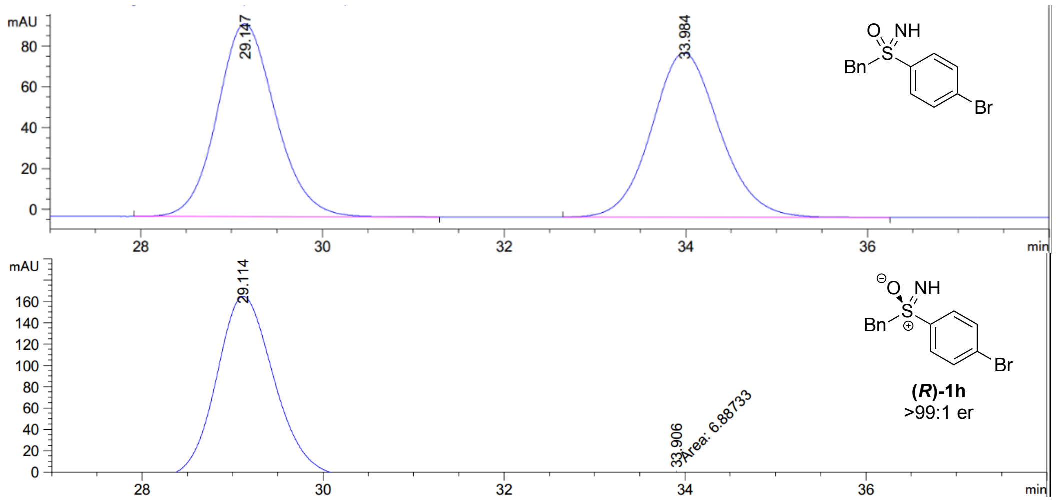

Peak #	RetTime [min]	Type	Width [min]	Area [mAU*s]	Height [mAU]	Area %
1	29.114	BB	0.6895	7656.42920	169.98912	99.9101
2	33.906	MP	0.1451	6.88733	7.52020e-1	0.0899

Totals : 7663.31653 170.74114

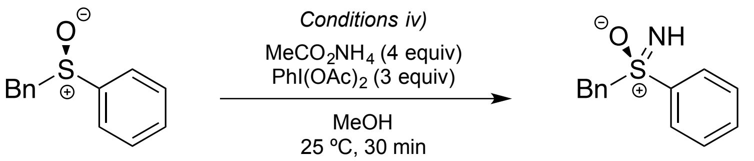

98.5:1.5 er

(*R*)-1b
98.5:1.5 er

Lux1, Hex:iPrOH (90:10) 0.5 ml/min

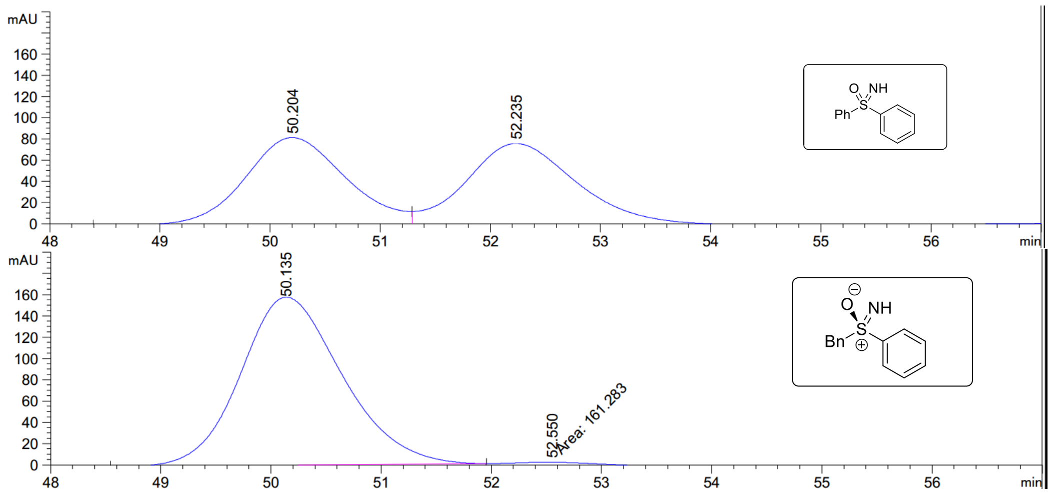

Peak #	RetTime [min]	Type	Width [min]	Area [mAU*s]	Height [mAU]	Area %
1	50.135	BB	0.9509	1.01329e4	157.86456	98.4333
2	52.550	MM T	0.8908	161.28304	3.01747	1.5667

Totals : 1.02942e4 160.88203

Investigation of the mechanism of the NH transfer

In order to get more insights into the mechanism of the reaction a NMR and HRMS investigation was performed in all the solvents.

Spectra obtained monitoring the progress of the reaction in d_8-toluene are reported in Figures 1-6.

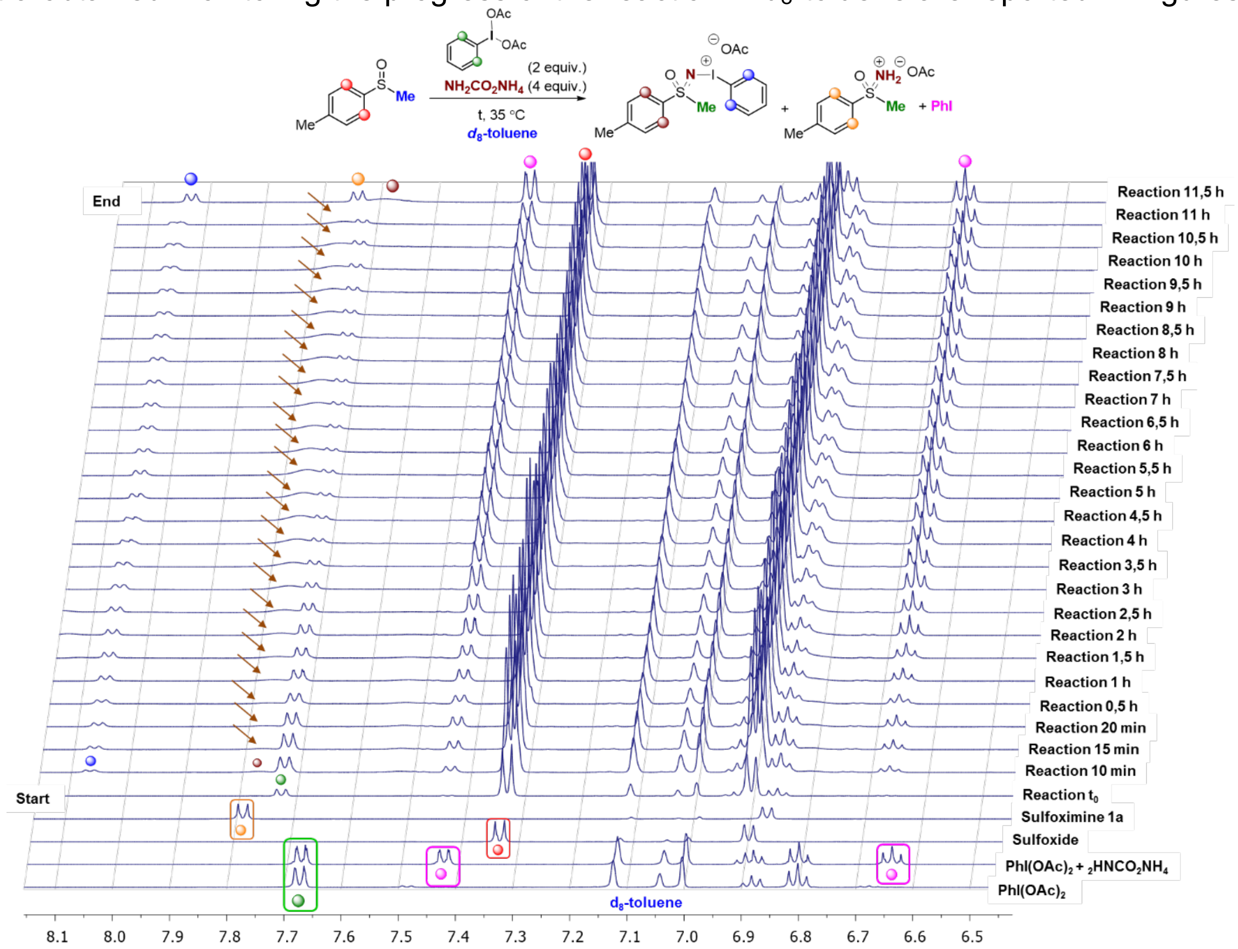

Supplementary Figure 1. Progress of the reaction of NH transfer monitored over 12 hours by 1H NMR (500 MHz, d_8-toluene, 35 °C). Reference spectrum reported at the bottom (aromatic region shown).

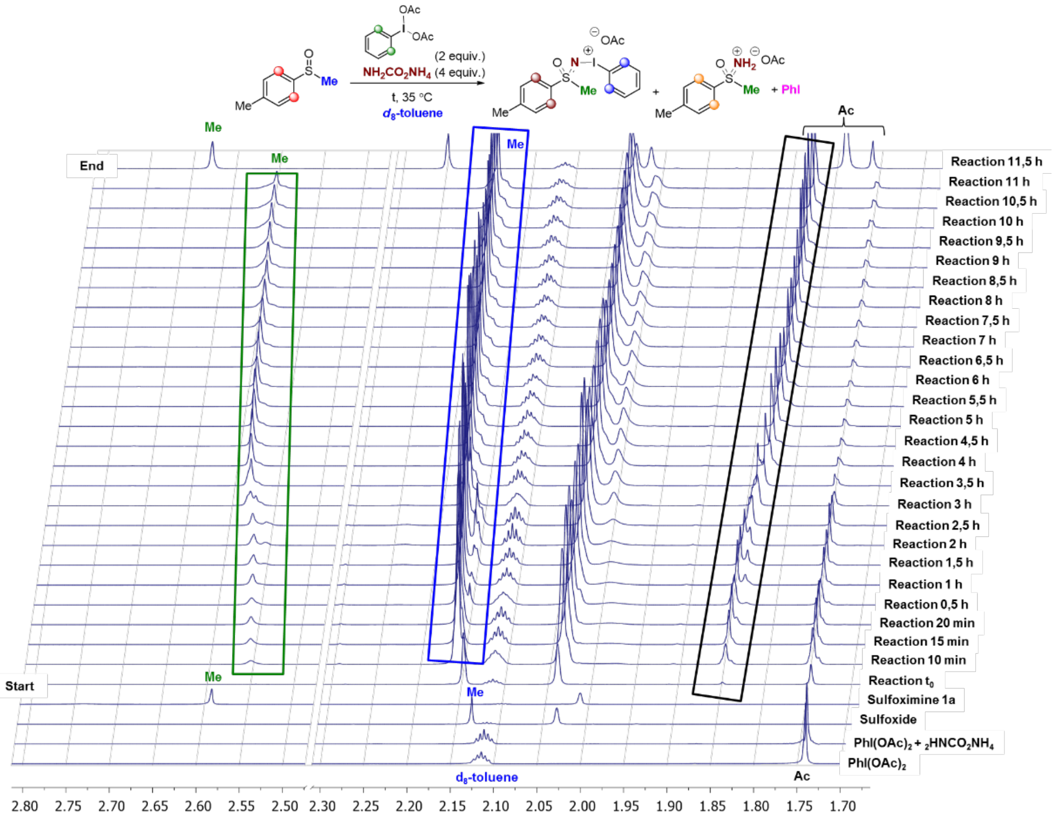

Supplementary Figure 2. Progress of the reaction of NH transfer monitored over 12 hours by 1H NMR (500 MHz, d_8-toluene, 35 °C). Reference spectrum reported at the bottom.

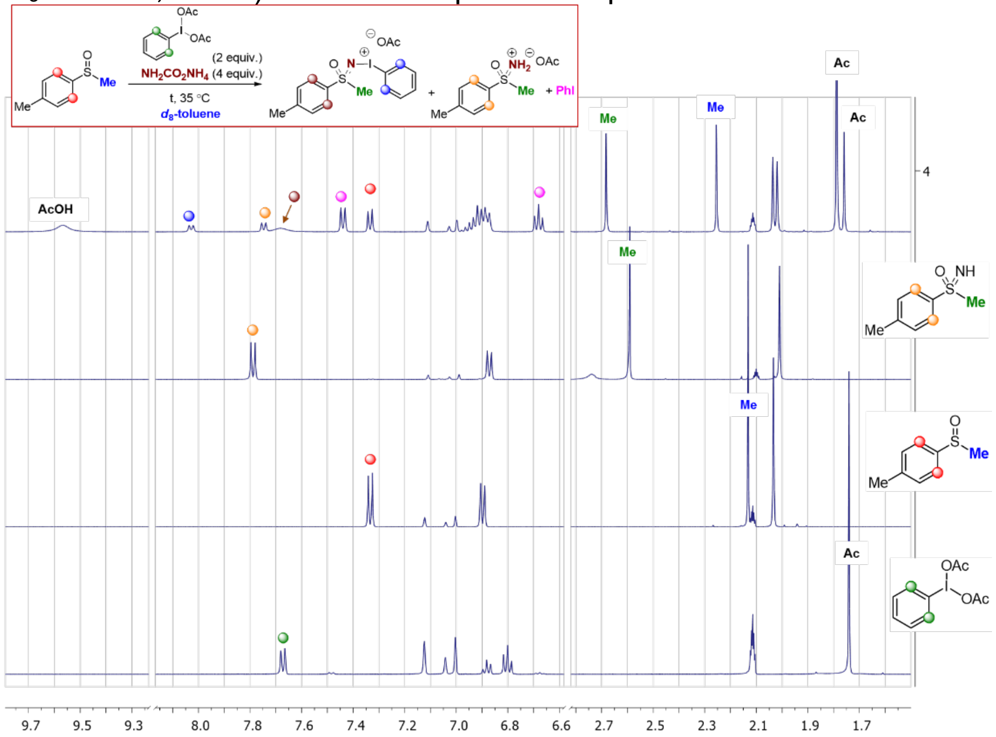

Supplementary Figure 3. Comparison of ^{1}H NMR spectra (500 MHz in d_8-toluene) of reference compounds and the reaction mixture observed after 12 hours.

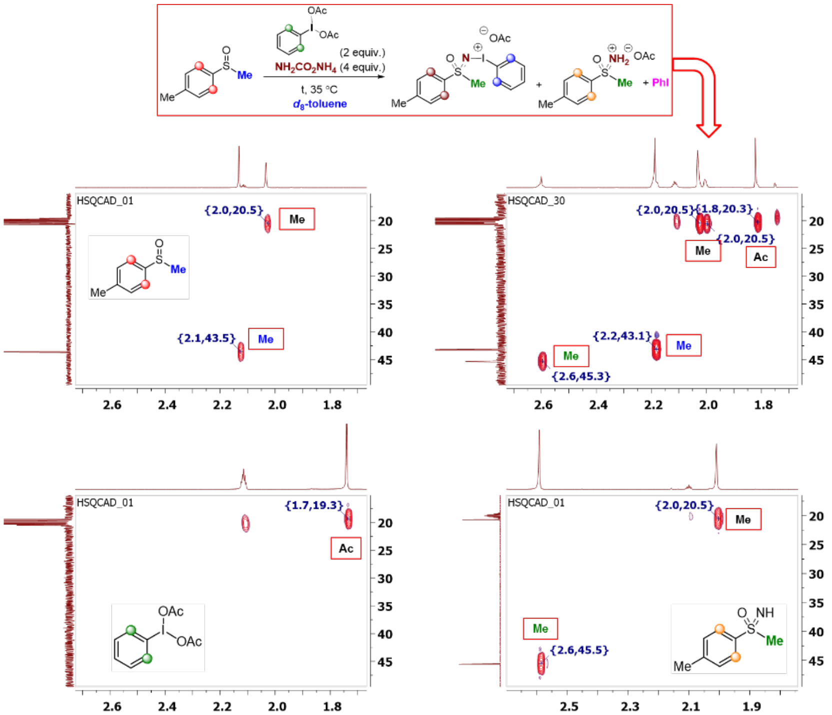

Supplementary Figure 4. Comparison of HSQC-DEPT spectra (500 MHz in d_8-toluene) of reference compounds and the reaction mixture observed after 12 hours.

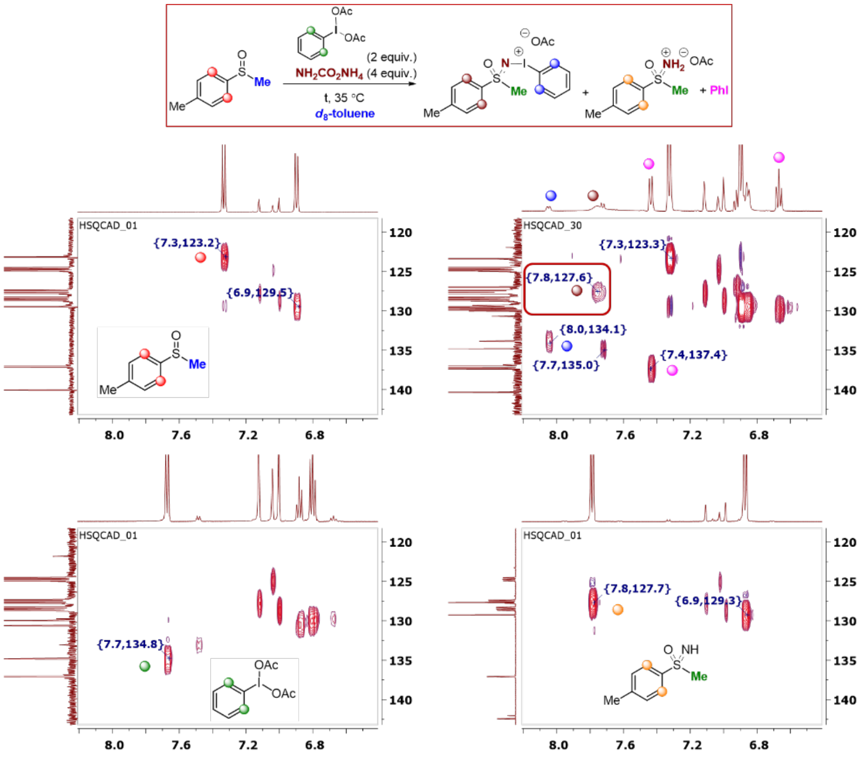

Supplementary Figure 5. Comparison of HSQC-DEPT spectra (500 MHz in d_8-toluene) of reference compounds and the reaction mixture observed after 12 hours (aromatic region shown).

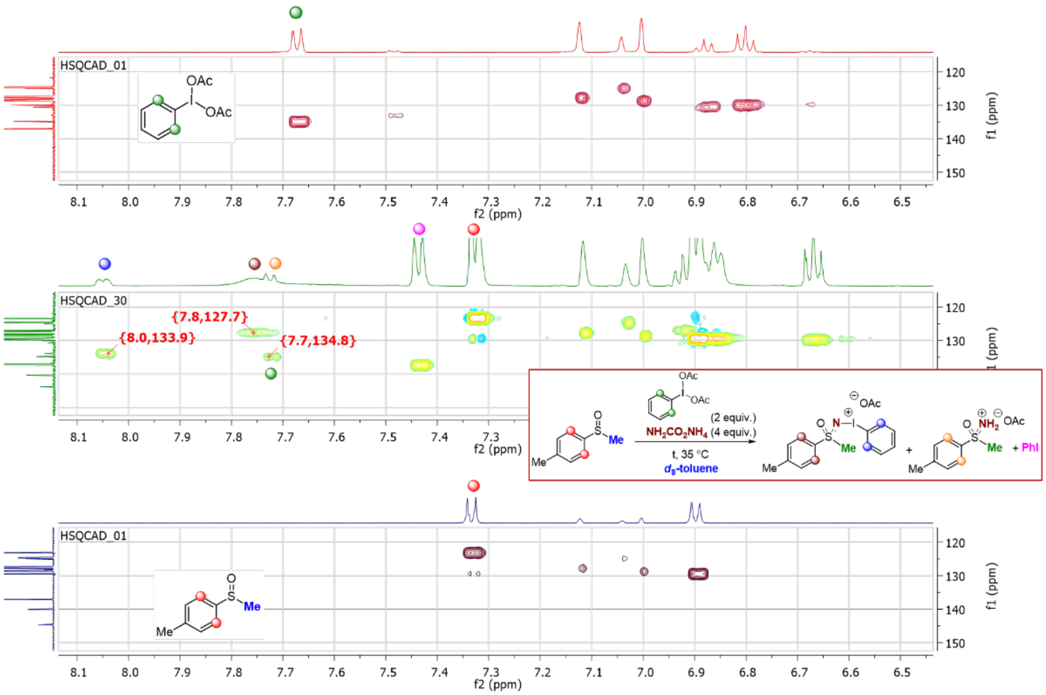

Supplementary Figure 6. Comparison of HSQC-DEPT spectra (500 MHz in d_8-toluene) of reference compounds and the reaction mixture observed after 12 hours (expansion for the aromatic region shown).

Other experiments were performed in CD_3OD (Supplementary Fig. 7 and 8) and in CD_3CN (Supplementary Fig. 9 and 10) as reaction solvents. The reaction run in those solvents are faster and go to completion (disappearance of the sulfoxide in minutes).

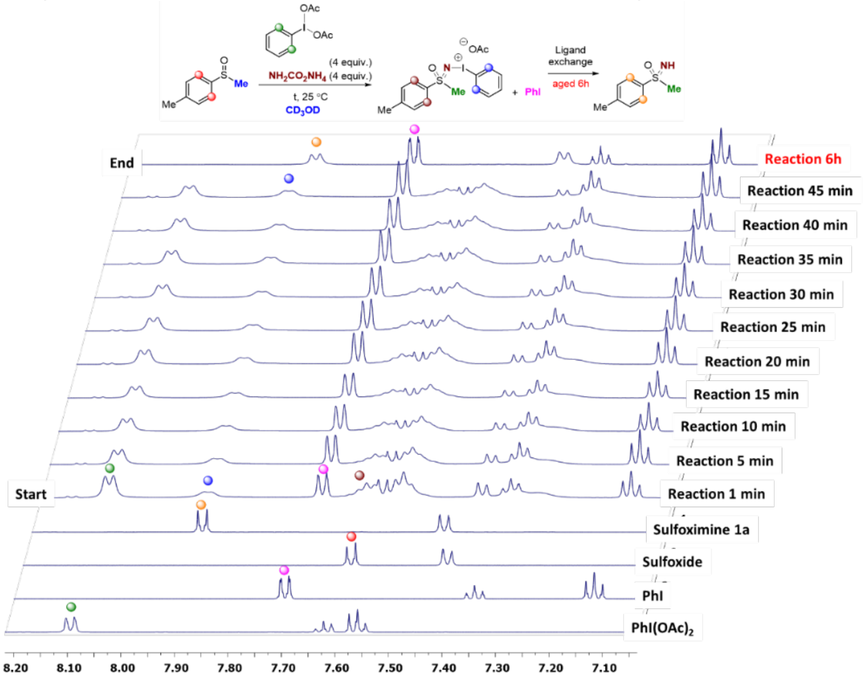

Supplementary Figure 7. Progress of the reaction of NH transfer monitored over 6 hours by ^1H NMR (500 MHz, CD_3OD, 25 °C). Reference spectrum reported at the bottom (aromatic region shown).

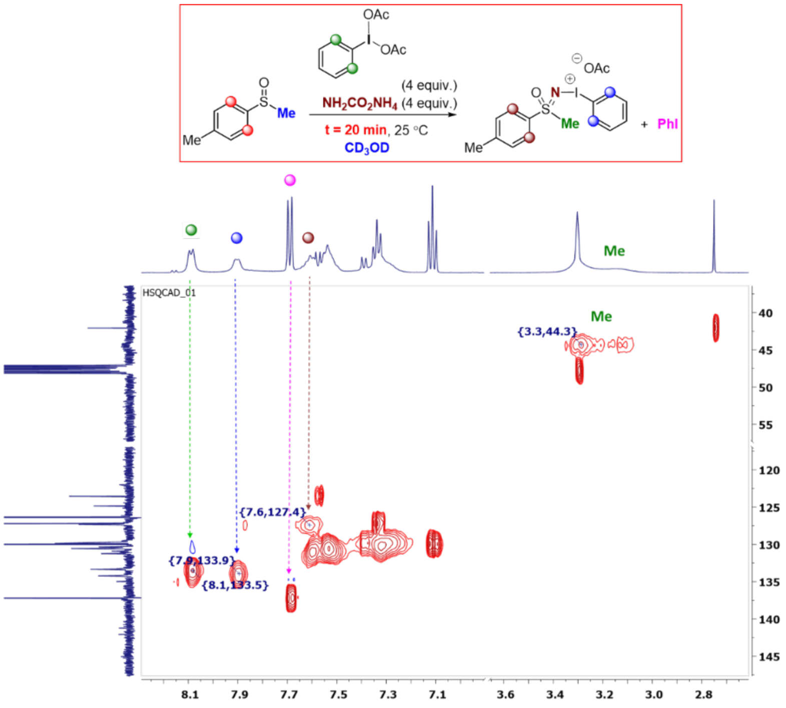

Supplementary Figure 8. HSQC-DEPT spectra (500 MHz in CD_3OD, 25 °C) of the NH transfer reaction observed after 20 min.

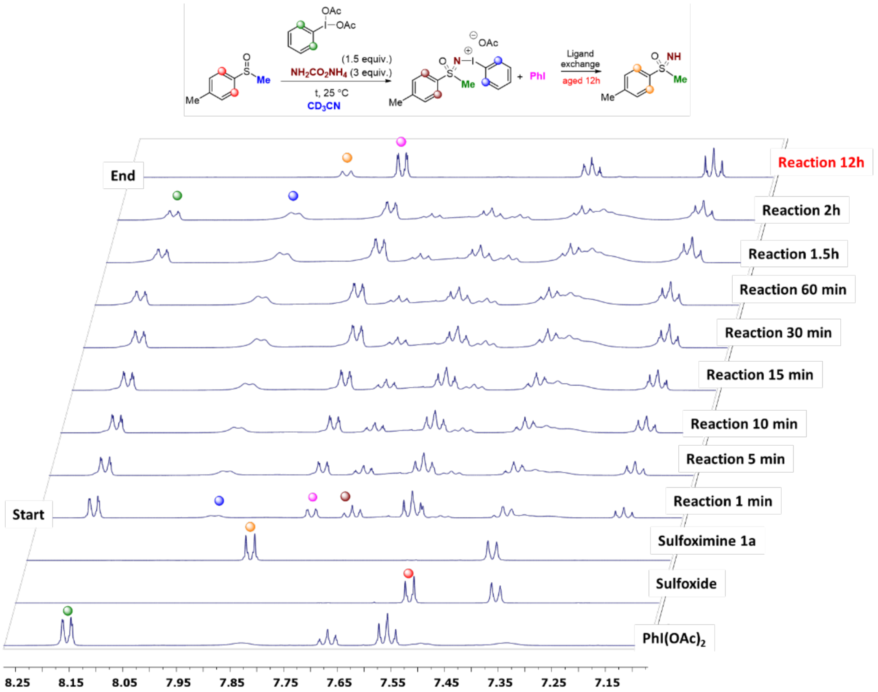

Supplementary Figure 9. Progress of the reaction of NH transfer monitored over 12 hours by ^{1}H NMR (500 MHz, CD_3CN, 25 °C). Reference spectrum reported at the bottom (aromatic region shown).

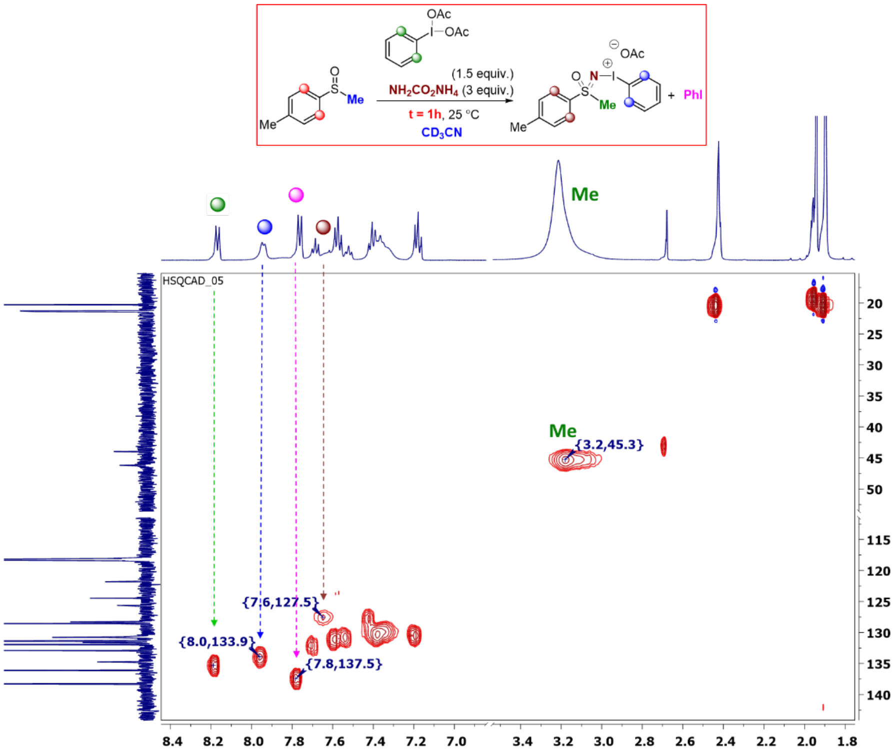

Supplementary Figure 10. HSQC-DEPT spectra (500 MHz in CD_3CN, 25 °C) of the NH transfer reaction observed after 20 min.

Comparison of HSQC DEPT spectra for the NH transfer reactions run in both CD_3OD and CD_3CN showed similarity in the chemical shifts of the involved species (Supplementary Fig. 11). In both solvents, a full conversion of the iodonium salt to the corresponding sulfoximine occurs in the NMR tube at long reaction time (Supplementary Fig. 12).

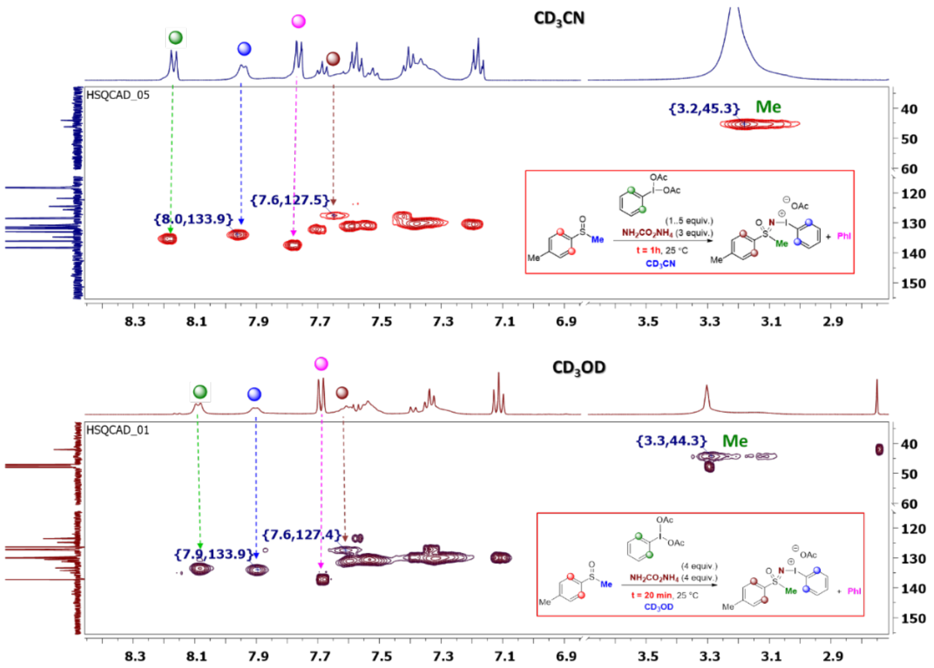

Supplementary Figure 11. Comparison of HSQC DEPT experiments (500 MHz) in CD_3OD and CD_3CN. Full conversion.

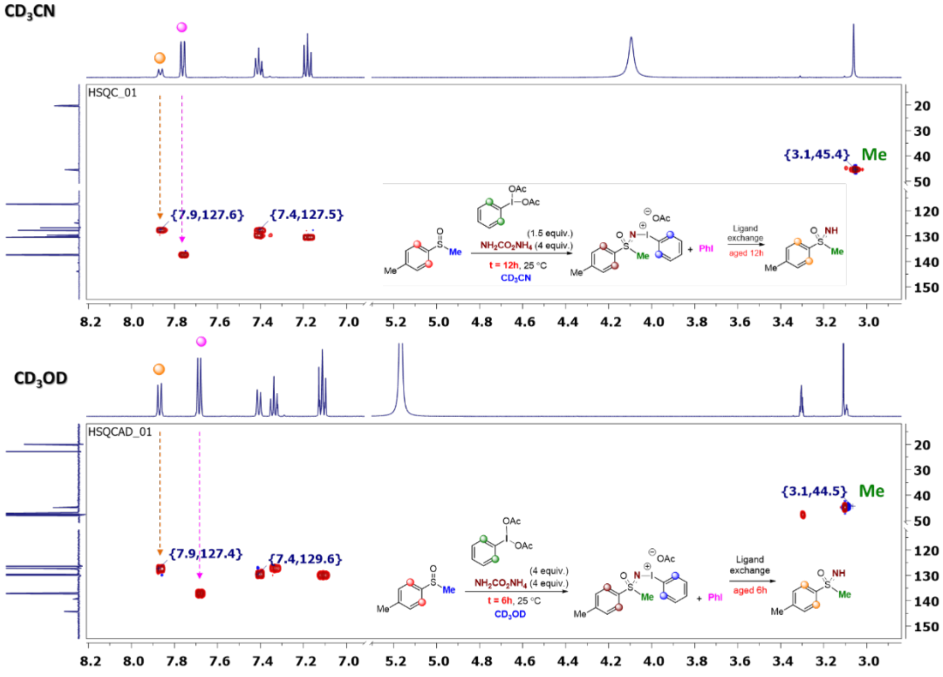

Supplementary Figure 12. Comparison of HSQC DEPT experiments (500 MHz) in CD_3OD and CD_3CN leaving the reaction mixture in the NMR tube for long time.

With the aim to get insights on the nature of the intermediates involved in the NH transfer, the progress of the reaction was monitored by HRMS using ESI source and a TOF detector. The experiment was run under flow conditions. The obtained spectra, reported in Supplementary Fig. 13, gave evidence on the presence of iodosylbenzene (PhI=O) and probably the iminoiodinane (PhI=NH) and iodonitrene (PhI=N$^+$) after mixing of PhI(OAc)$_2$ and ammonium carbamate. However, after adding the sulfoxide, the only detectable species was the hypervalent iodonium salt just as seen by NMR.

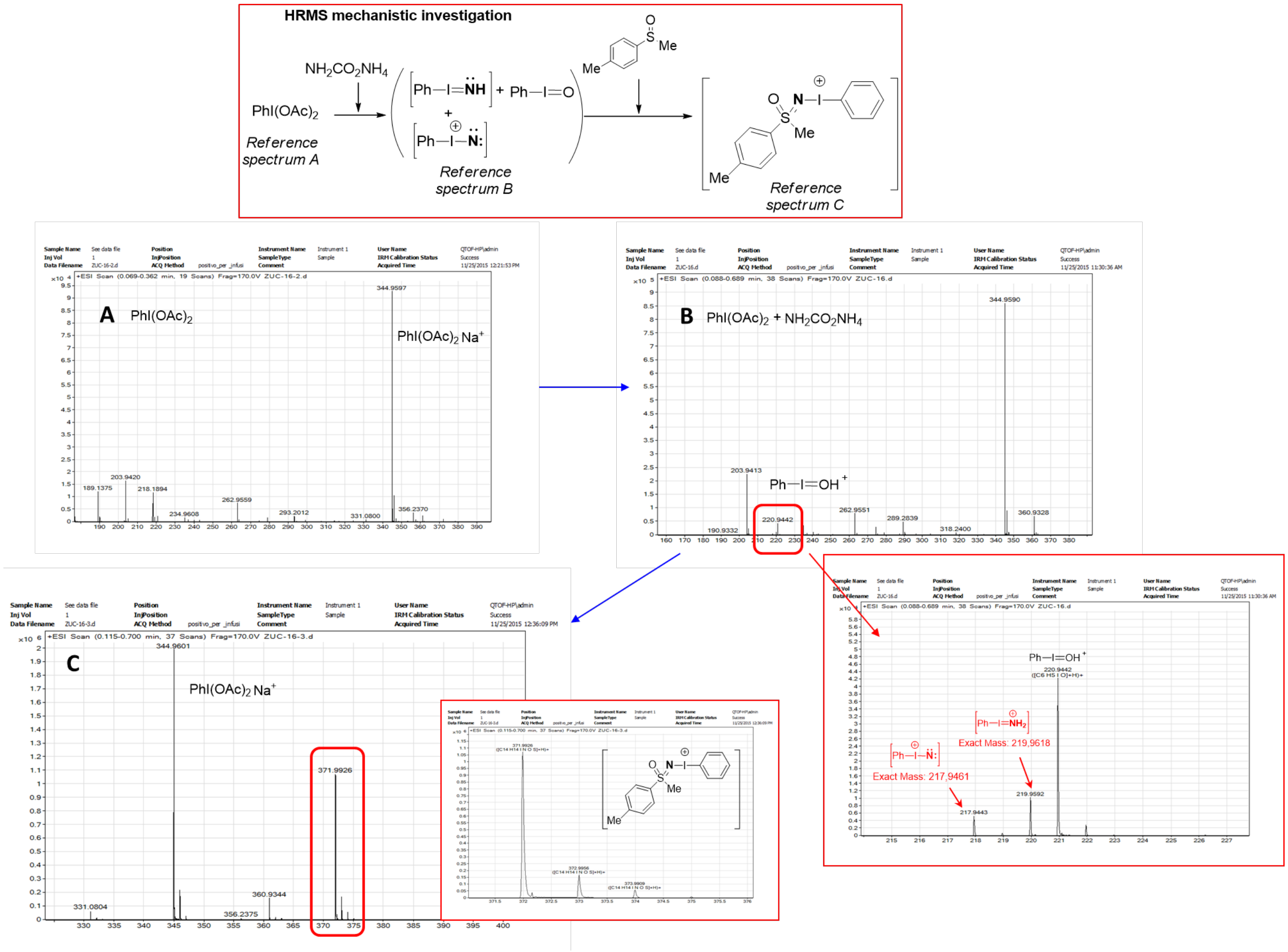

Supplementary Figure 13. HRMS experiment conducted under flow conditions.

Experiment with ^{15}N labeled source of ammonia.

In order to probe the presence of the iodonitrene ($PhI{=}N^+$), the HRMS flow experiment was run with ^{15}N-labeled ammonia generated by using readily available ^{15}N ammonium acetate. Supplementary Figure 13b reports the observed MS signals with isotopic shift. Supplementary figure 13a is included for comparison.

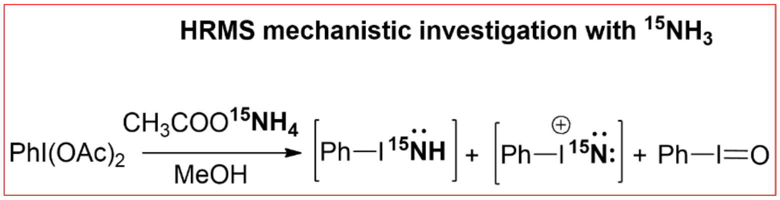

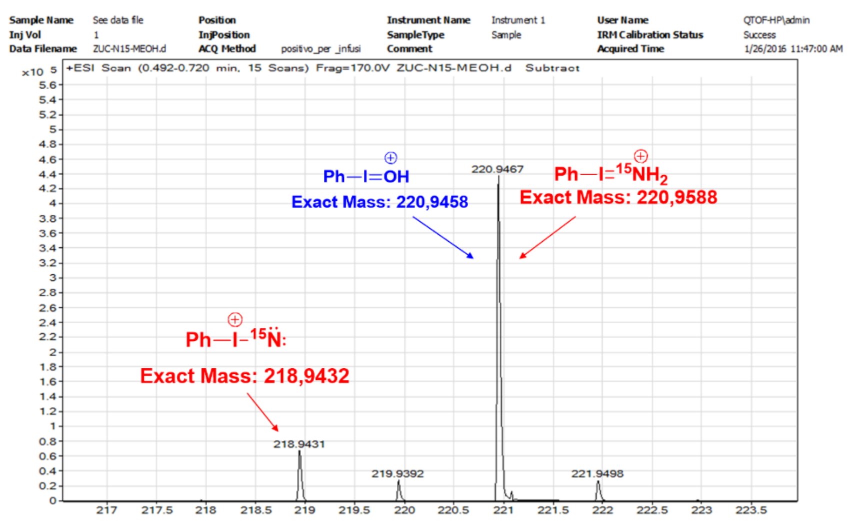

Supplementary Figure 13a. HRMS spectrum obtained using ammonium acetate as source of ^{15}N labeled ammonia. The signals assigned to the iodosylbenzene and iodonitrene can be recognized.

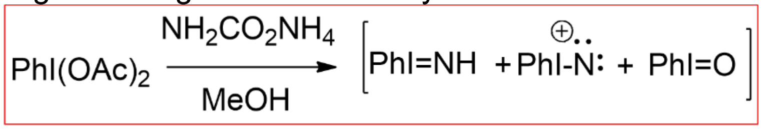

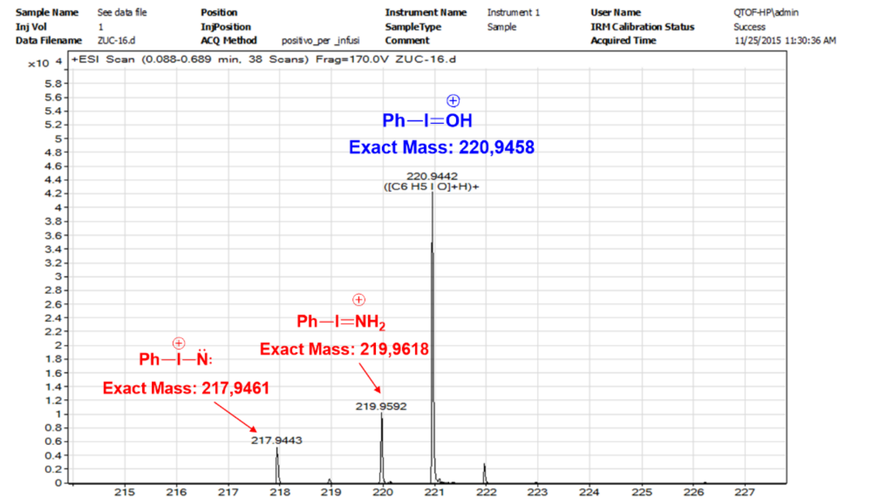

Supplementary Figure 13b. Reference HRMS spectrum obtained using ammonium carbamate as source of ammonia. The signals assigned to the iodosylbenzene, iminoiodinane and iodonitrene can be recognized.

The hypervalent iodonium salt was independently prepared by mixing the sulfoximine **1a** with $PhI(OAc)_2$ in CD_3CN and the spectra compared with that obtained in the NH transfer reaction in the same solvent (Supplementary Fig. 14-16). The reaction is not as fast as expected and it takes 2 hours to go to completion (Supplementary Fig. 15).

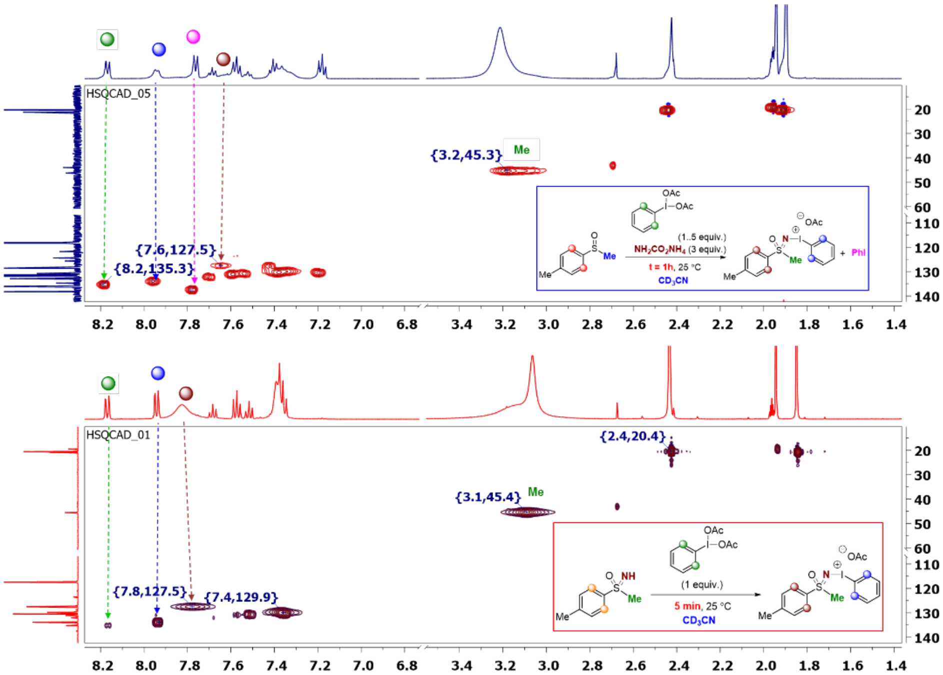

Supplementary Figure 14. Comparison of HSQC DEPT spectra (500 MHz, CD_3CN) confirming the structure of the hypervalent iodonium salt.

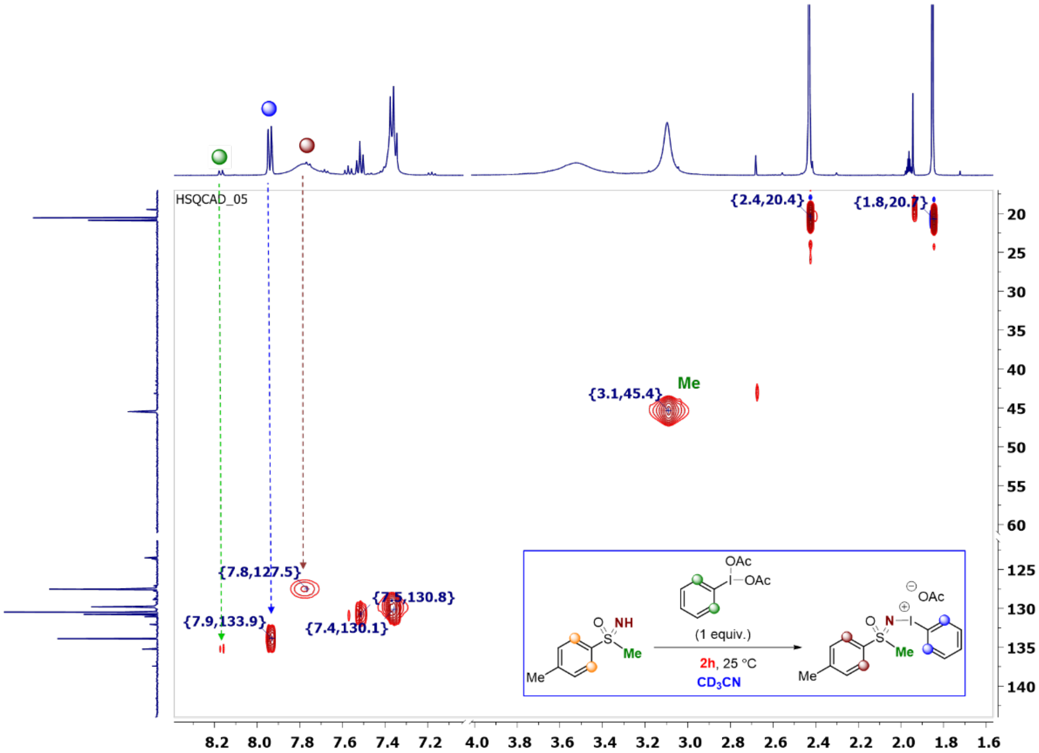

Supplementary Figure 15. HSQC DEPT spectra (500 MHz, CD_3CN) of the hypervalent iodonium salt generated by mixing sulfoximine **1a** with $PhI(OAc)_2$ in CD_3CN and monitored after 2 hours.

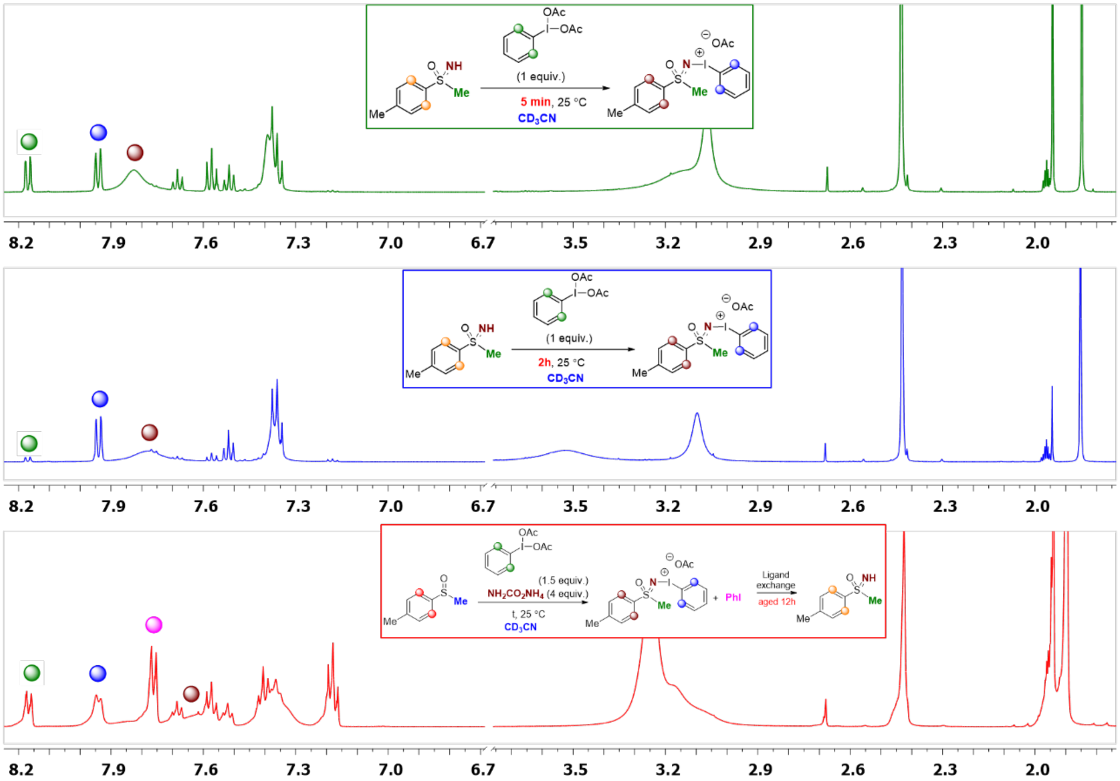

Supplementary Figure 16. Comparison of ^{1}H NMR spectra (500 MHz, CD_3CN) confirming the structure of the hypervalent iodonium salt.

The same experiment reported above has been conducted in $CDCl_3$ obtaining similar conclusions (Supplementary Fig. 17 and 19). These NMR data has been useful to confirm assignments in the experiments run in toluene, MeOH and acetonitrile.

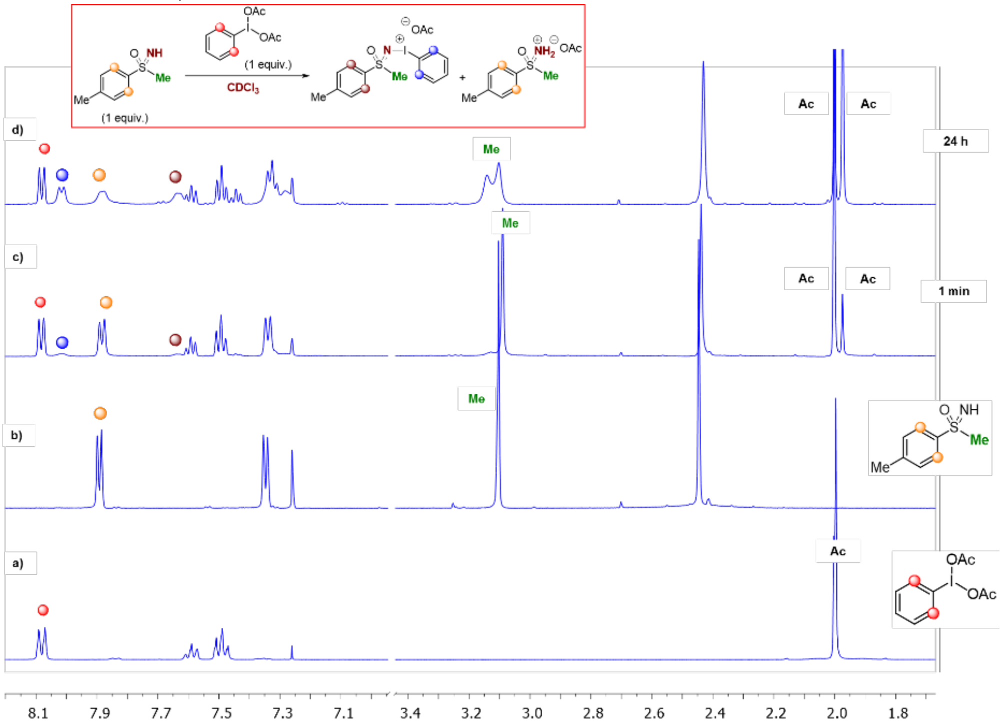

Supplementary Figure 17. Preparation of the hypervalent iodonium salt in CDCl3. Comparison of ^{1}H NMR spectra (500 MHz, CDCl3)

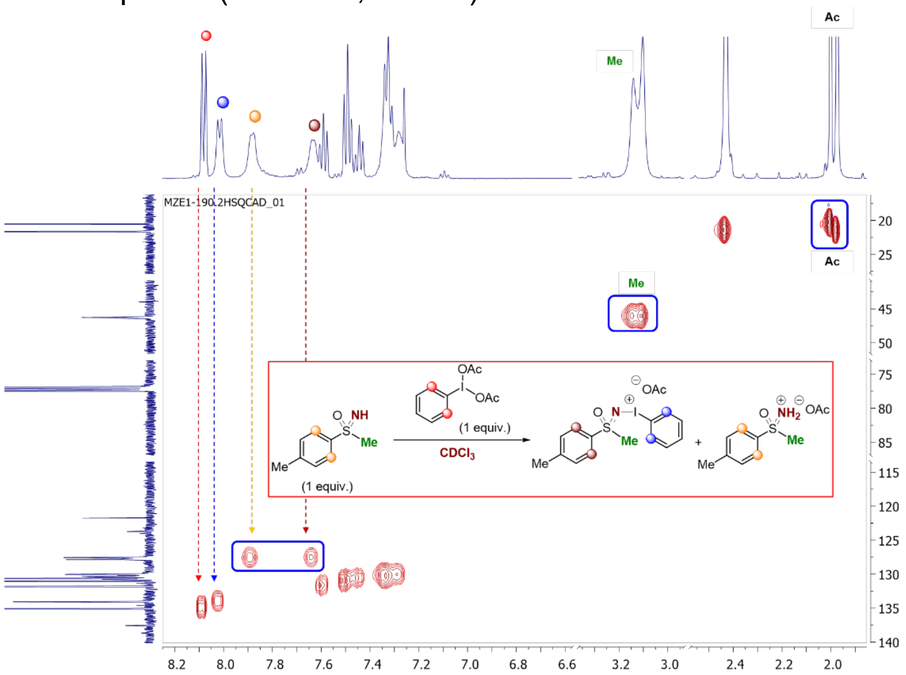

Supplementary Figure 18. Preparation of the hypervalent iodonium salt in CDCl3. HSQC DEPT experiment (500 MHz, CDCl3).

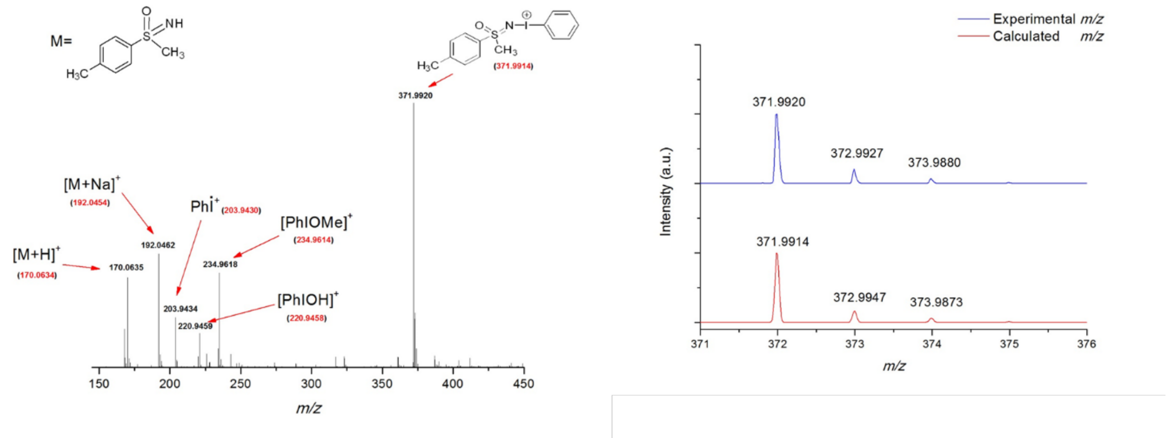

Supplementary Figure 19. HRMS analysis of the reaction mixture obtained in $CDCl_3$.

The role of ammonia as nitrogen source was demonstrated by the experiments reported in Supplementary Fig. 20.

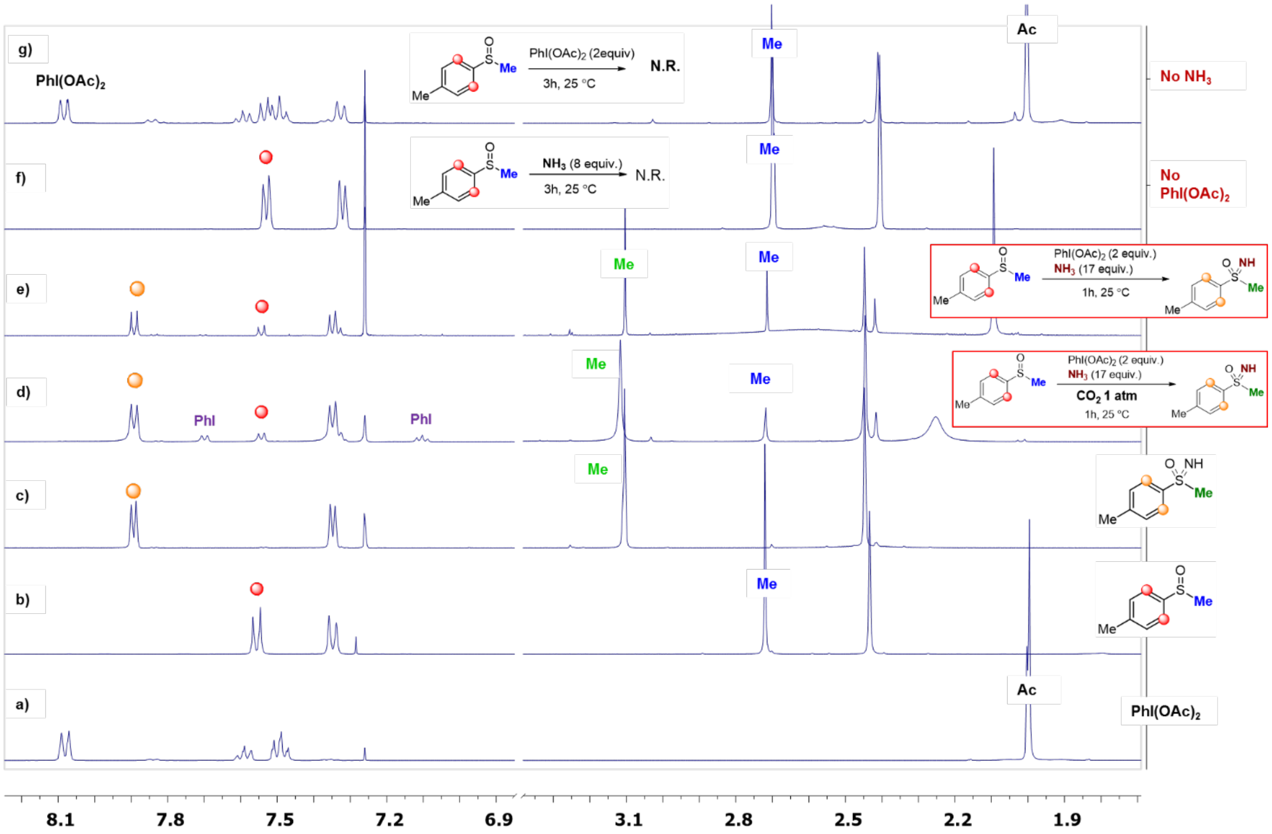

Supplementary Figure 20. a) Reference spectrum for $PhI(OAc)_2$; b) reference spectrum for tolyl methylsulfoxide; c) reference spectrum for sulfoximine **1a**; d) Crude of the reaction run in the presence of ammonia and $PhI(OAc)_2$ under CO_2 atmosphere; e) Crude of the reaction run in the presence of ammonia and $PhI(OAc)_2$; f) Crude of the reaction run in the presence of ammonia without $PhI(OAc)_2$; g) Crude of the reaction run in the presence of $PhI(OAc)_2$ without ammonia.

^{1}H and ^{13}C NMR spectra of selected compounds

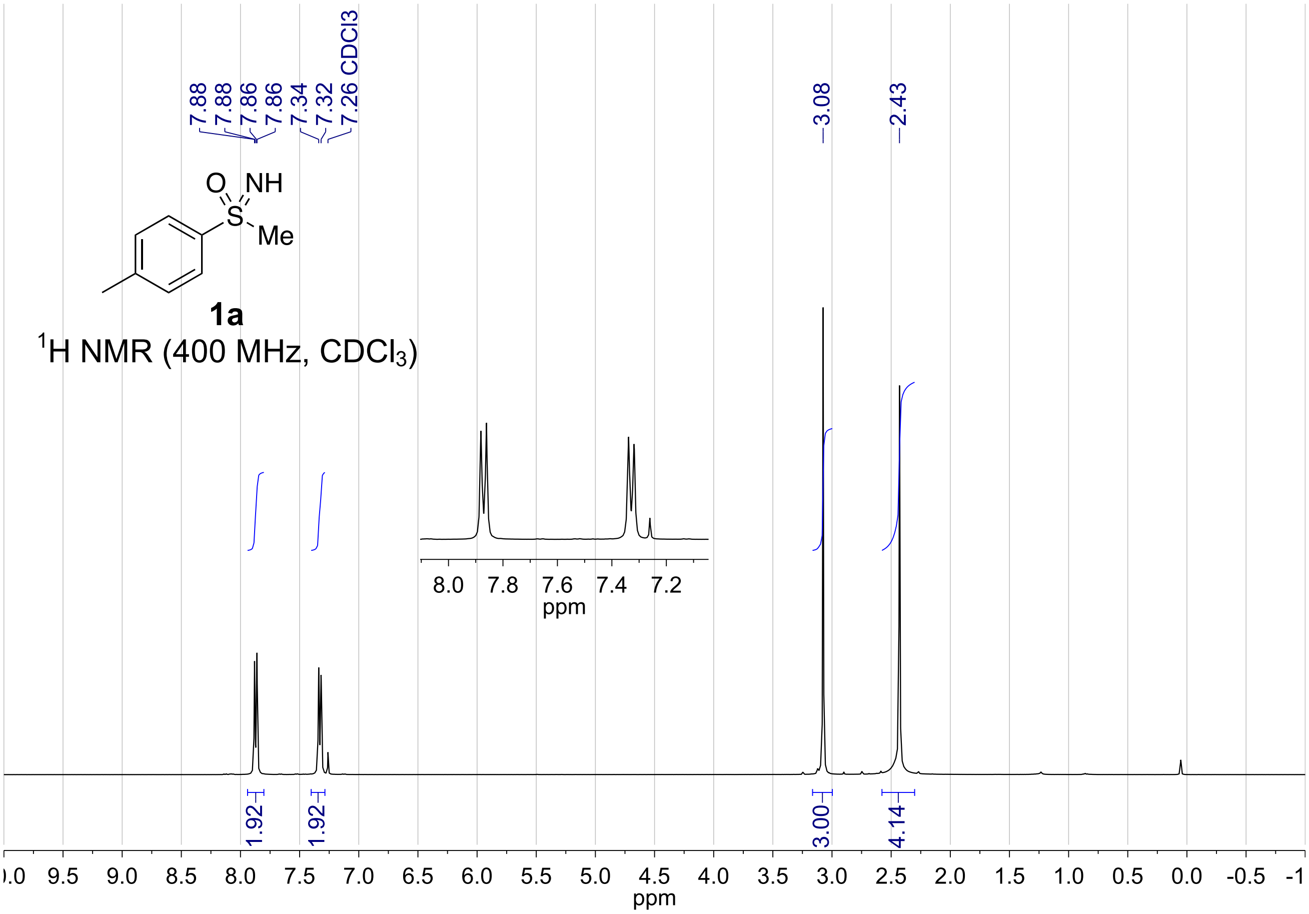
7.88
7.88
7.86
7.86
7.34
7.32
7.26 CDCl3
3.08
2.43
O
NH
S
Me
1a
1H NMR (400 MHz, CDCl3)
1.92
1.92
3.00
4.14
ppm

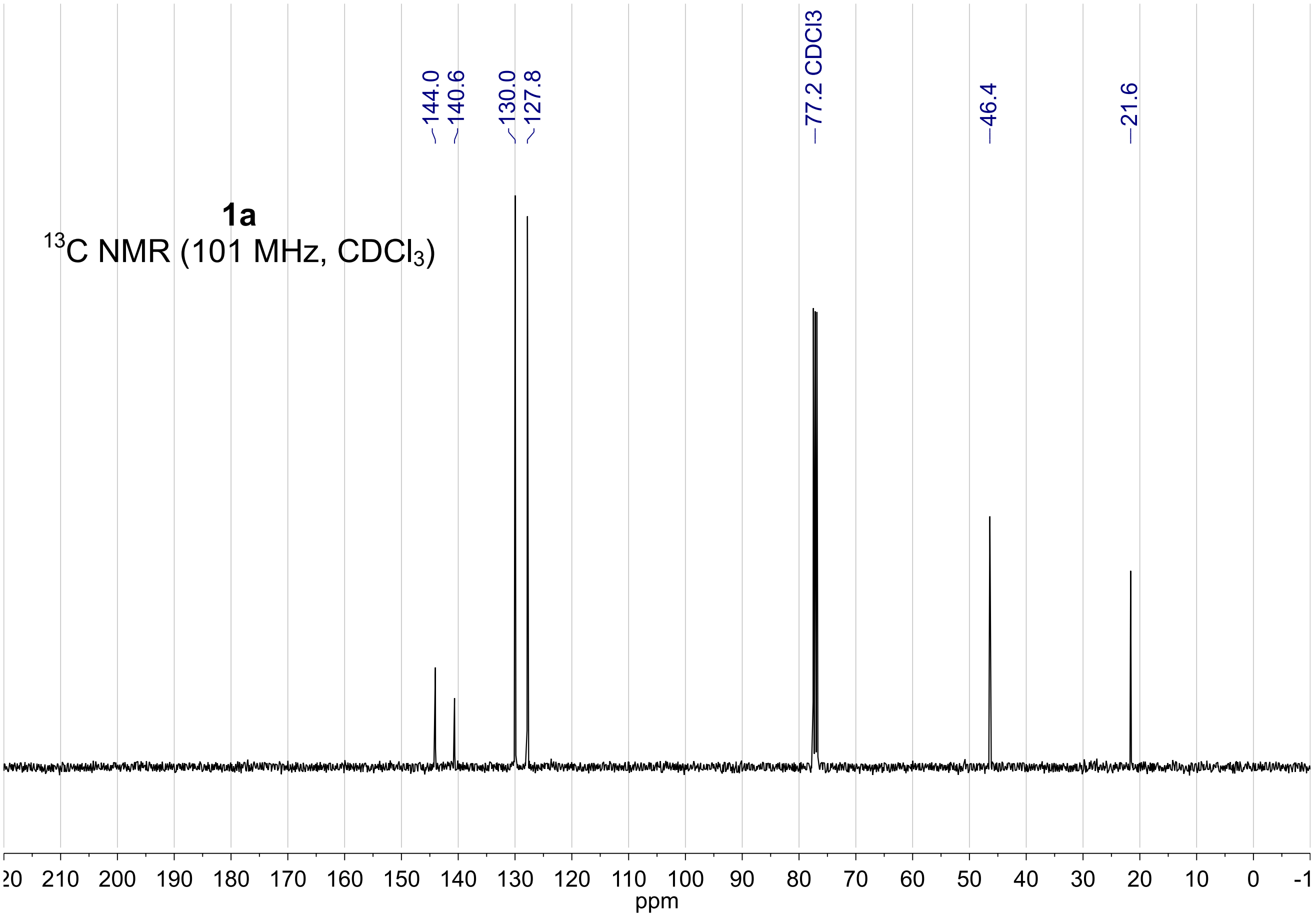
144.0
140.6
130.0
127.8
77.2 CDCl3
46.4
21.6
1a
13C NMR (101 MHz, CDCl3)
ppm

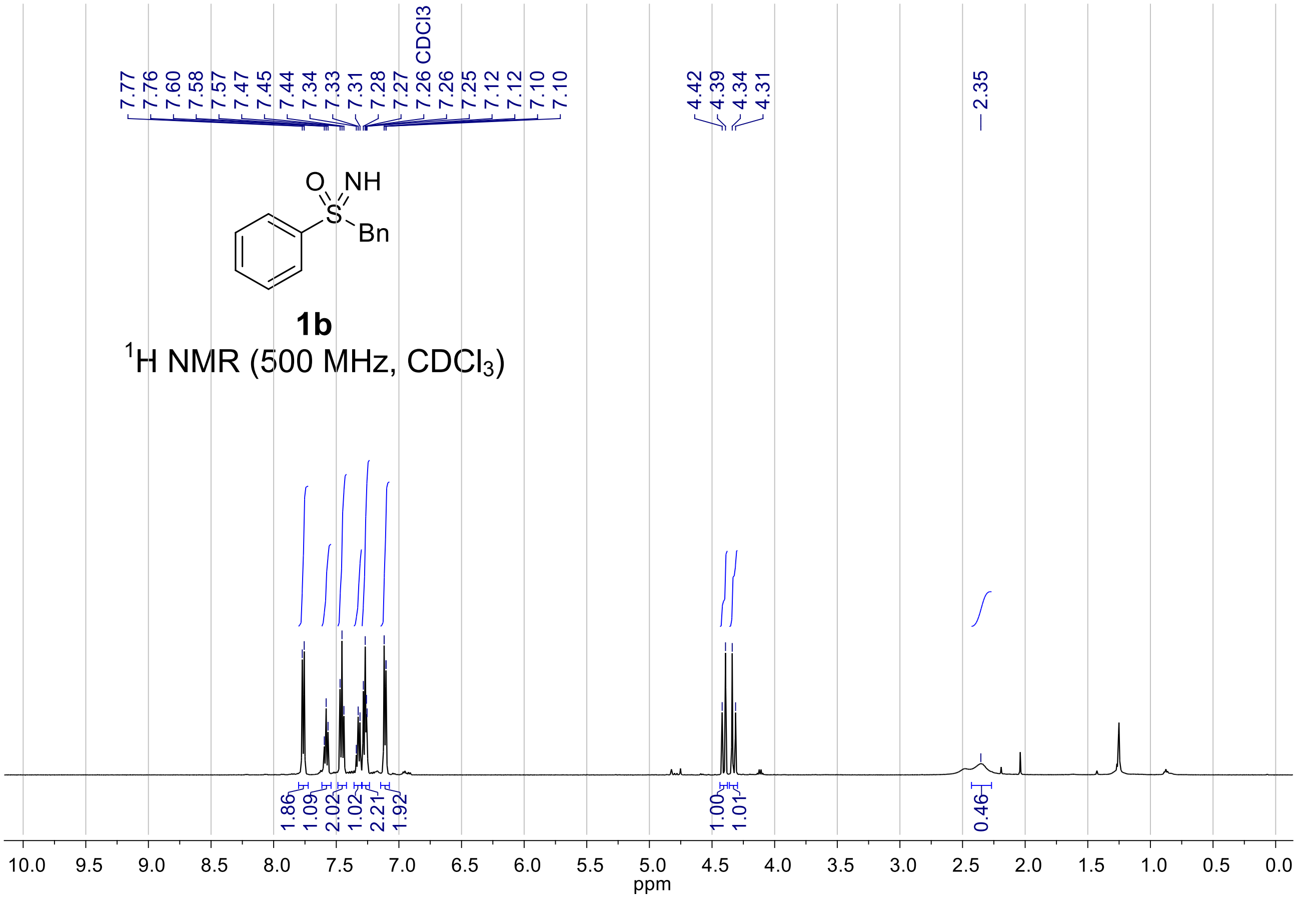
7.77 7.76 7.60 7.58 7.57 7.47 7.45 7.44 7.34 7.33 7.31 7.28 7.27 7.26 CDCl3 7.26 7.25 7.12 7.12 7.10 7.10
4.42 4.39 4.34 4.31
2.35
O NH
S
Bn
1b
^{1}H NMR (500 MHz, CDCl$_3$)
1.86 1.09 2.02 1.02 2.21 1.92
1.00 1.01
0.46
10.0 9.5 9.0 8.5 8.0 7.5 7.0 6.5 6.0 5.5 5.0 4.5 4.0 3.5 3.0 2.5 2.0 1.5 1.0 0.5 0.0
ppm

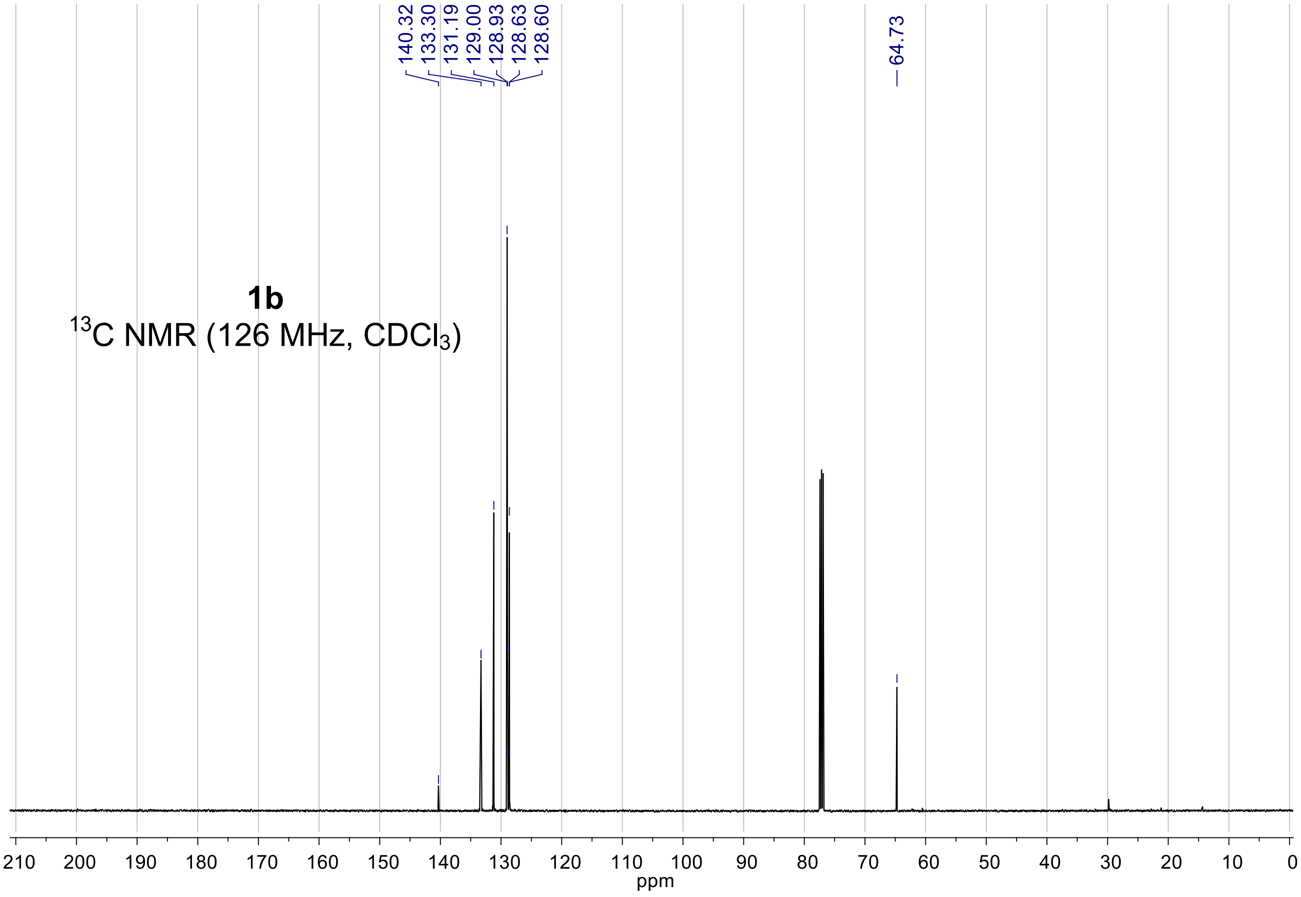
140.32 133.30 131.19 129.00 128.93 128.63 128.60
64.73
1b
^{13}C NMR (126 MHz, CDCl$_3$)
210 200 190 180 170 160 150 140 130 120 110 100 90 80 70 60 50 40 30 20 10 0
ppm

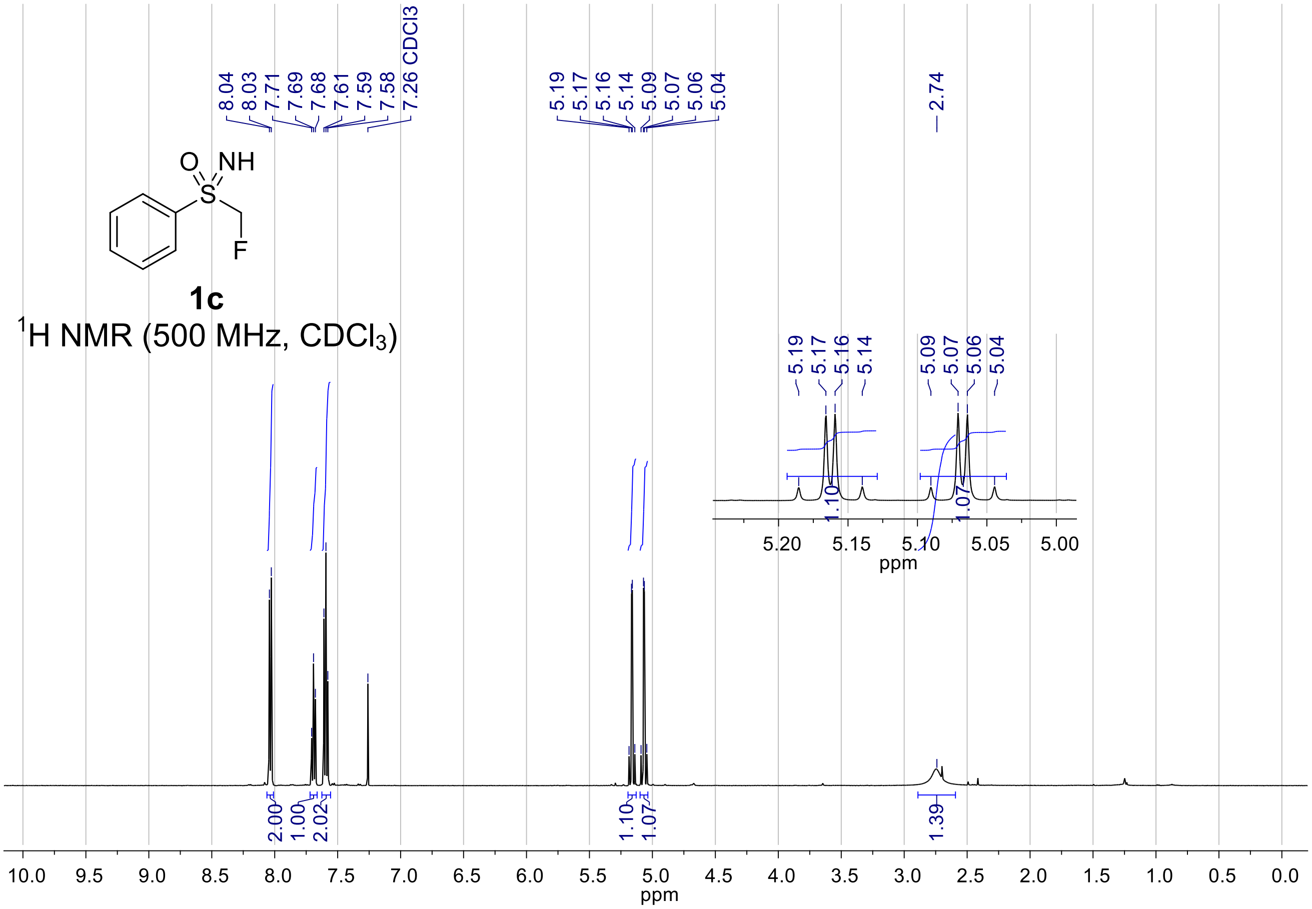

1c

^{1}H NMR (500 MHz, $CDCl_3$)

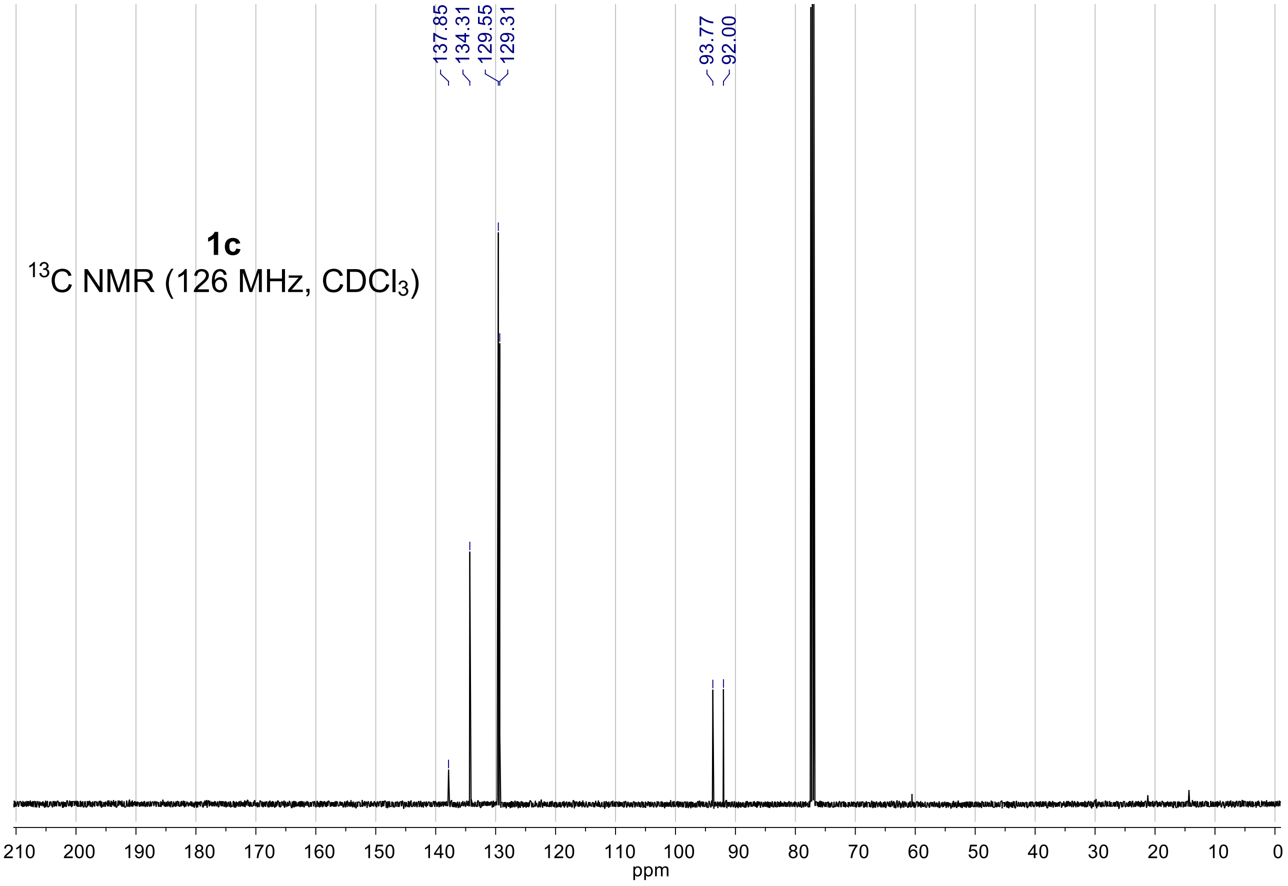

1c

^{13}C NMR (126 MHz, $CDCl_3$)

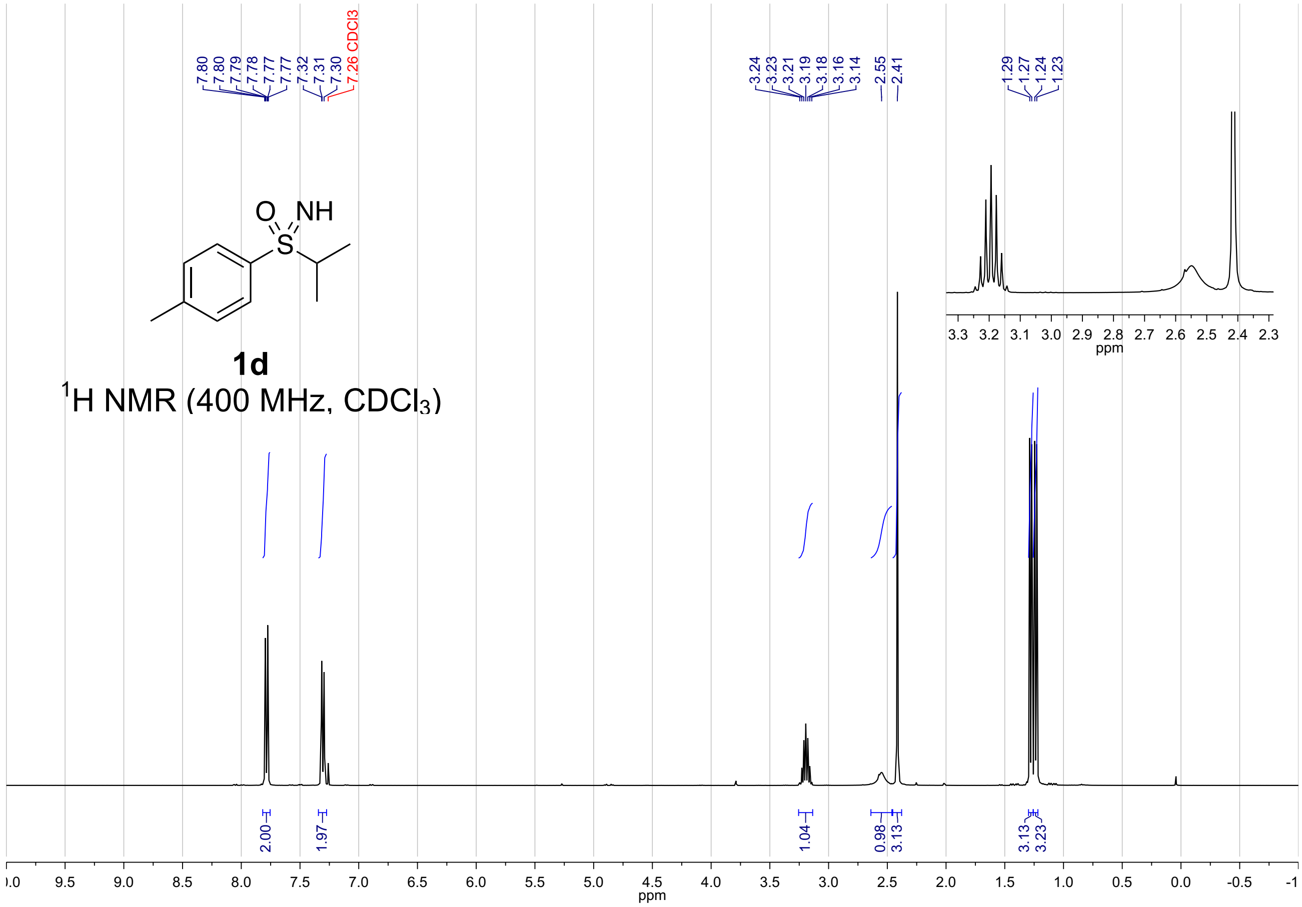
7.80
7.80
7.79
7.78
7.77
7.77
7.32
7.31
7.30
7.26 CDCl3
3.24
3.23
3.21
3.19
3.18
3.16
3.14
2.55
2.41
1.29
1.27
1.24
1.23
O NH
S
1d
1H NMR (400 MHz, CDCl3)
2.00
1.97
1.04
0.98
3.13
3.13
3.23
ppm

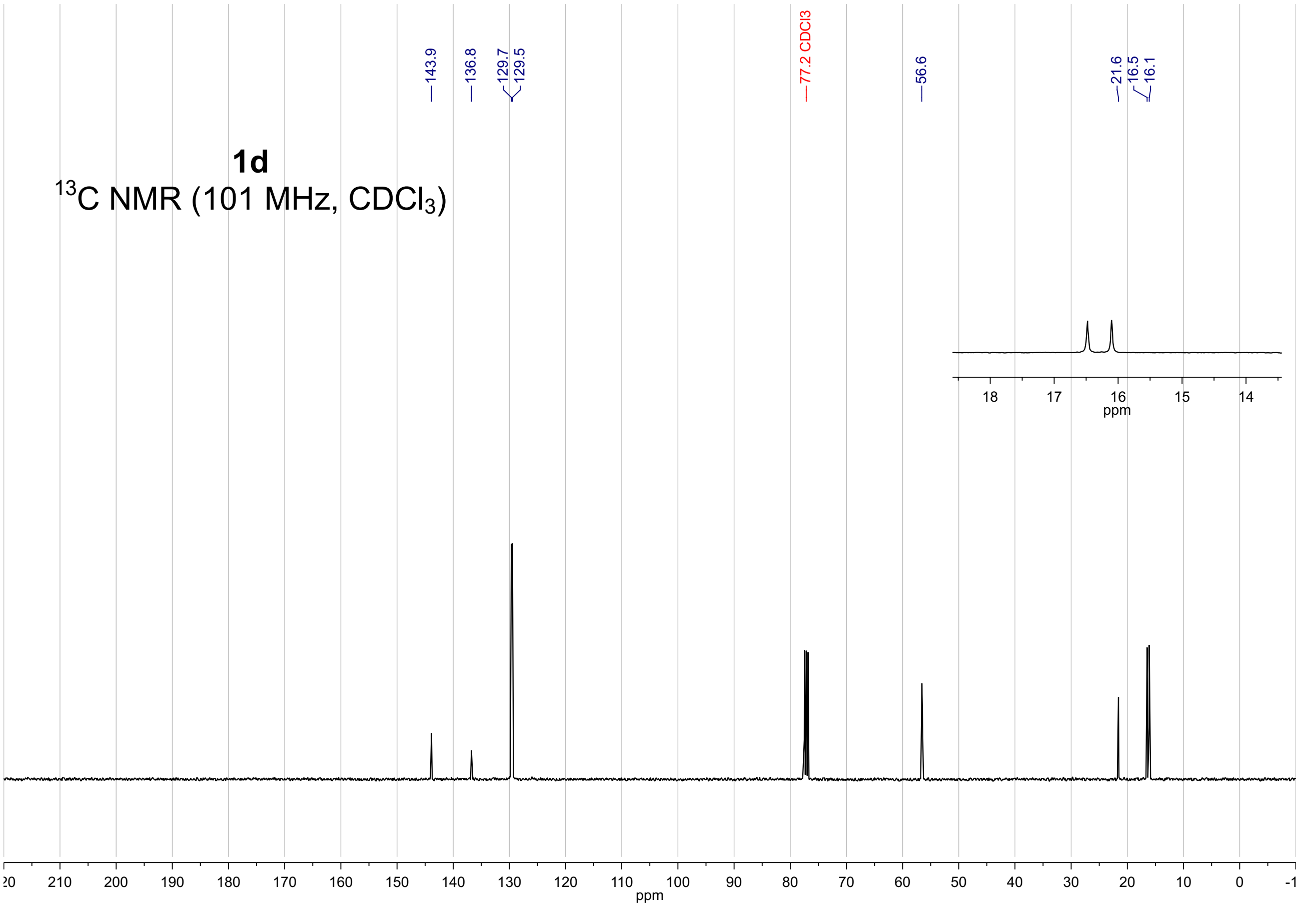
143.9
136.8
129.7
129.5
77.2 CDCl3
56.6
21.6
16.5
16.1
1d
13C NMR (101 MHz, CDCl3)
ppm

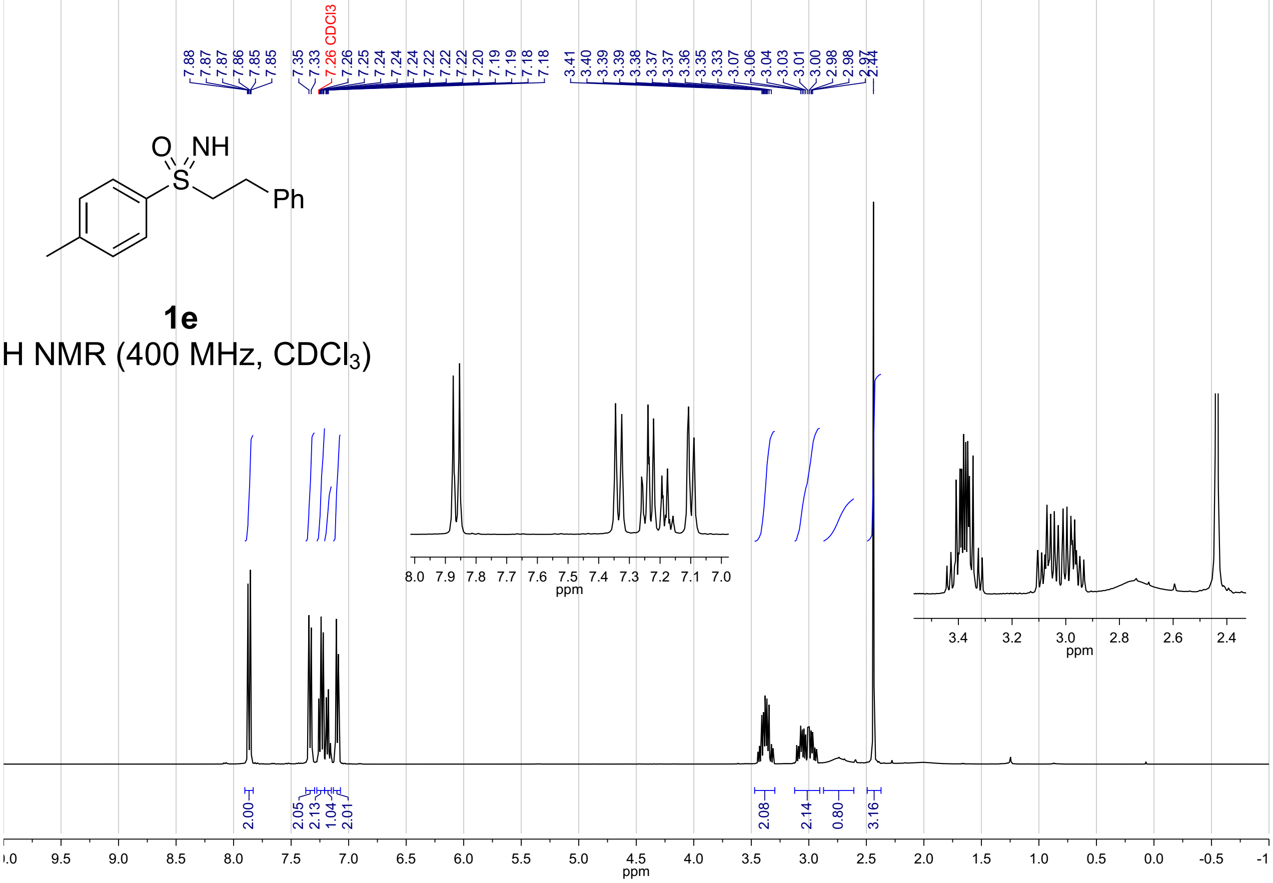

1e
^{1}H NMR (400 MHz, $CDCl_3$)

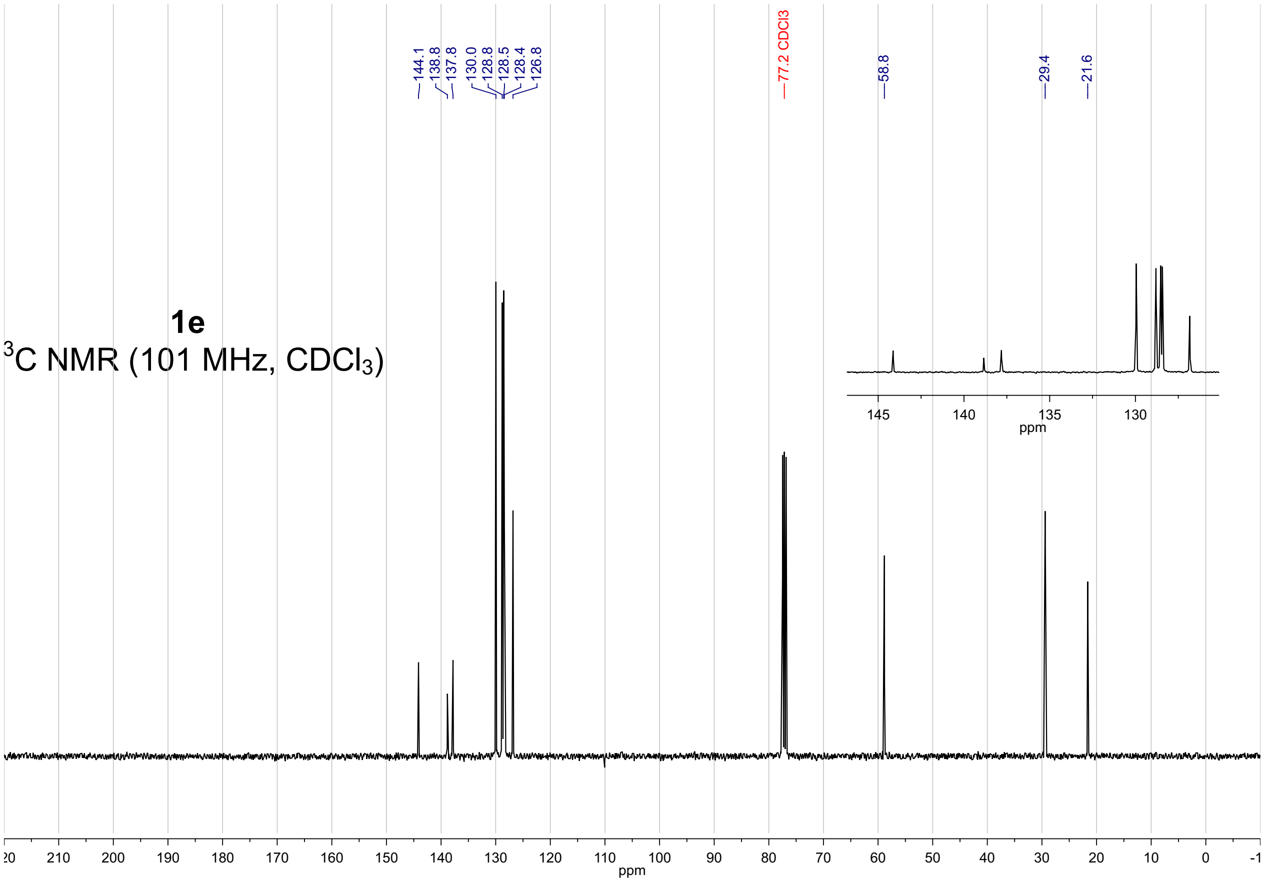

1e
^{13}C NMR (101 MHz, $CDCl_3$)

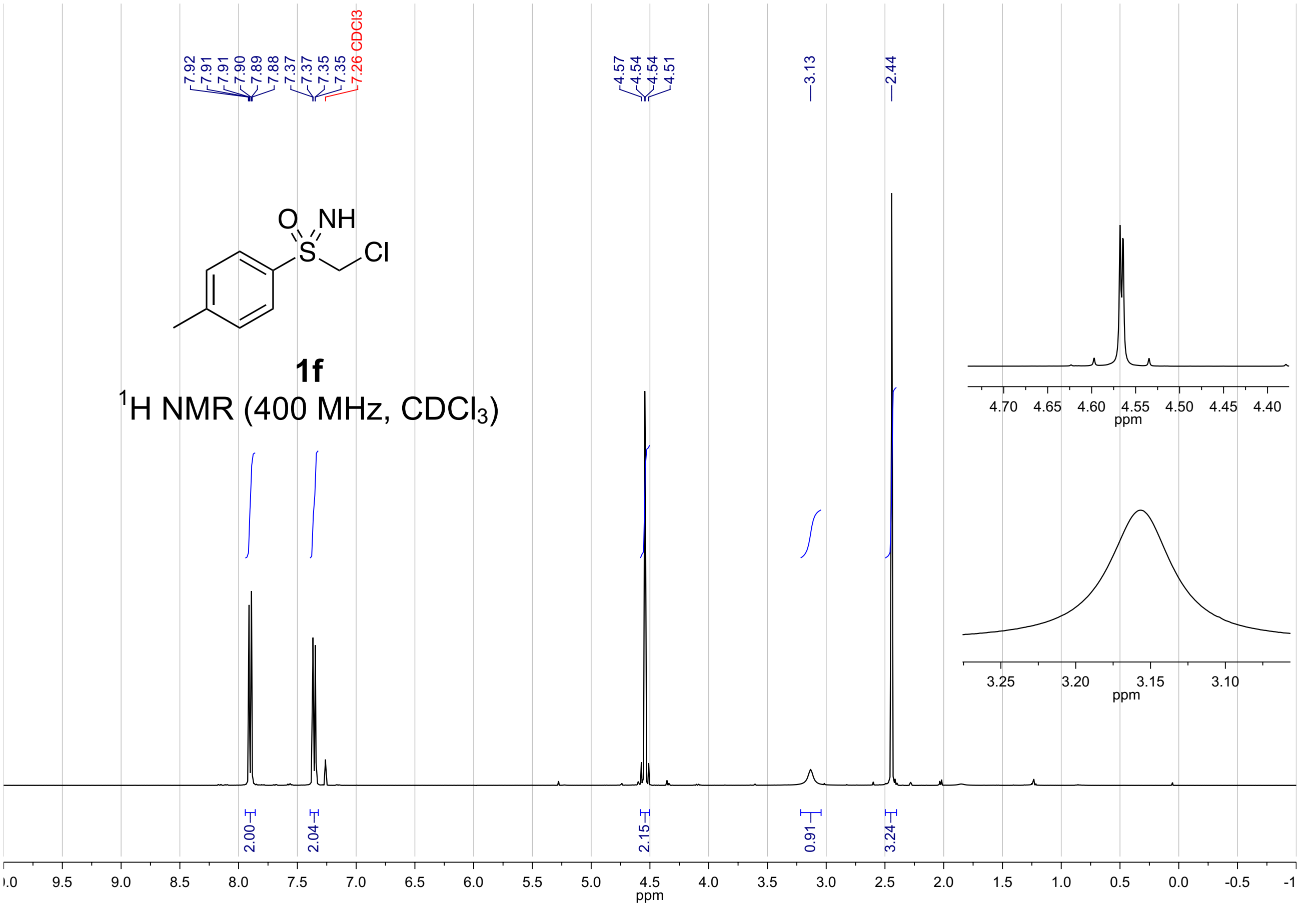
7.92 7.91 7.91 7.90 7.89 7.88 7.37 7.37 7.35 7.35 7.26 CDCl3
4.57 4.54 4.54 4.51
3.13
2.44
1f
1H NMR (400 MHz, CDCl3)
2.00
2.04
2.15
0.91
3.24
ppm

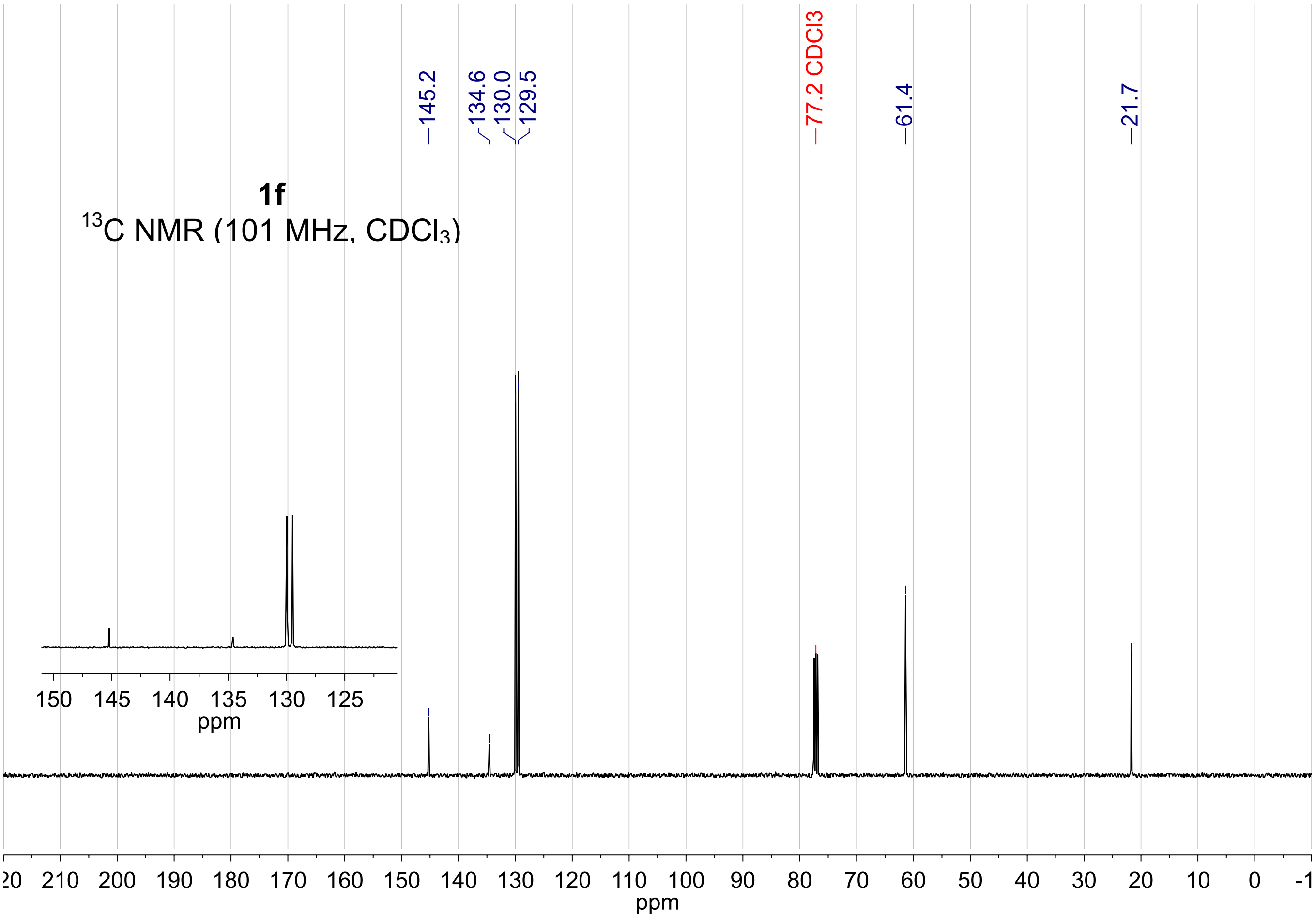
145.2
134.6
130.0
129.5
77.2 CDCl3
61.4
21.7
1f
13C NMR (101 MHz, CDCl3)
ppm

1g

^{1}H NMR (400 MHz, $CDCl_3$)

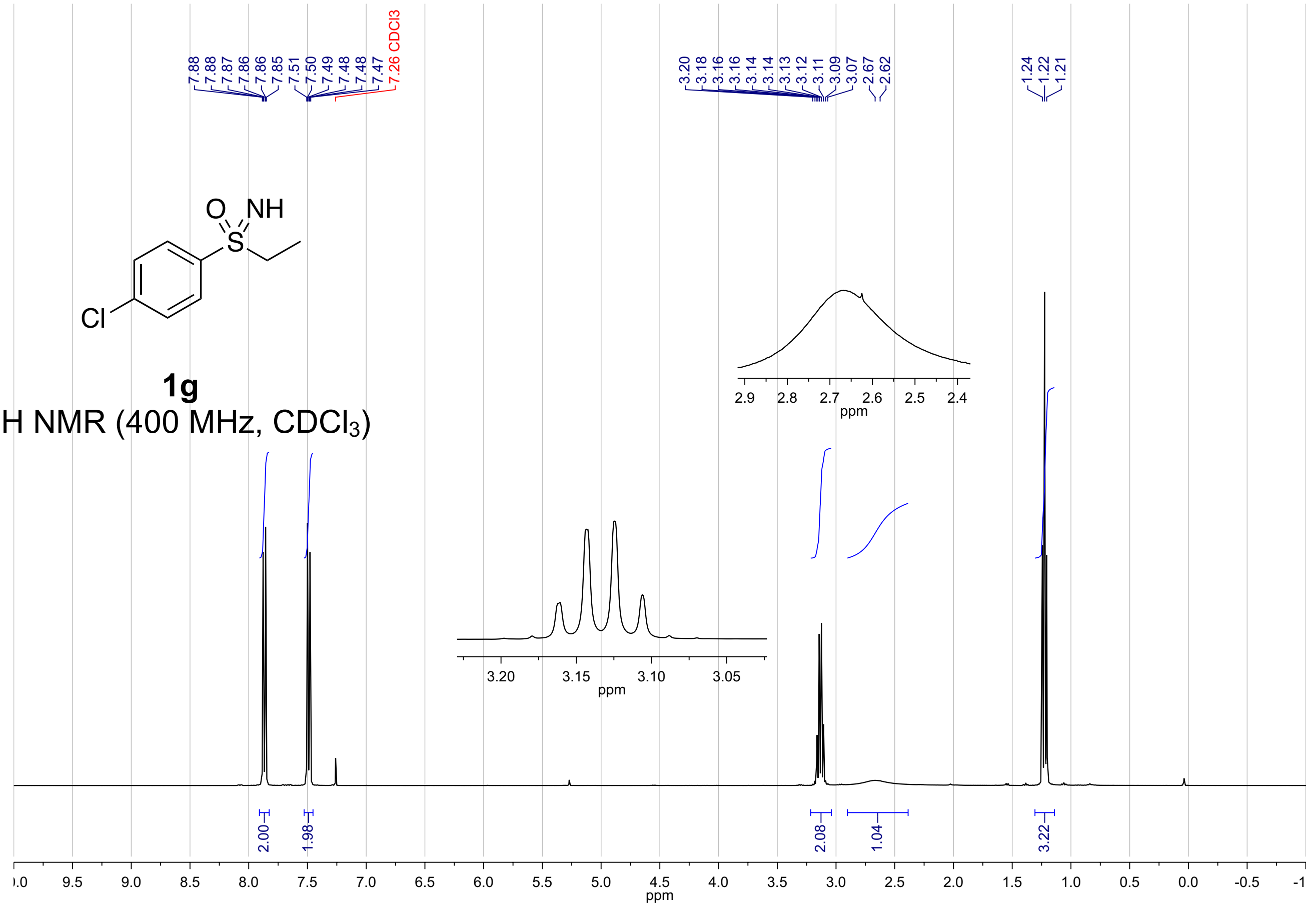

1g

^{13}C NMR (101 MHz, $CDCl_3$)

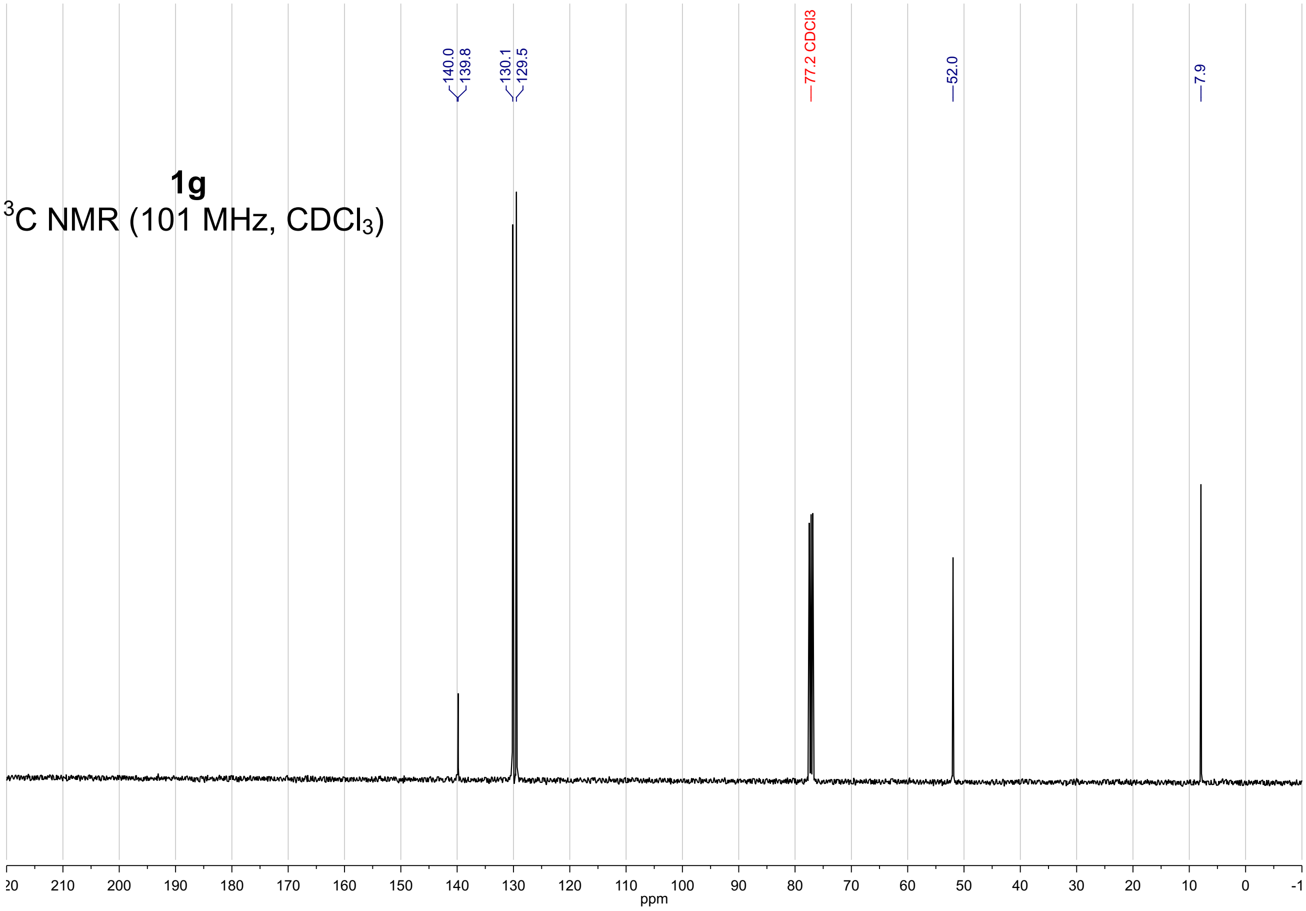

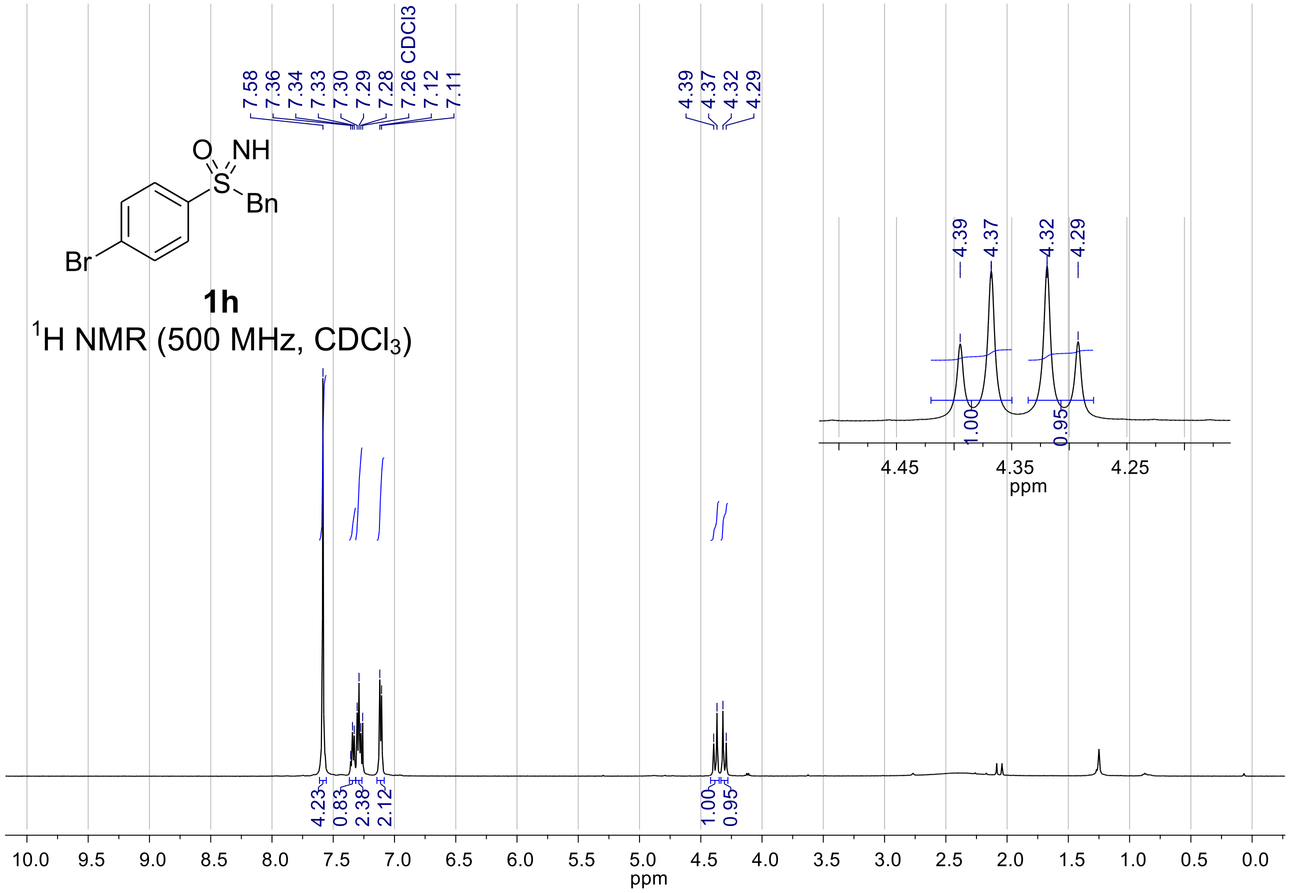
7.58
7.36
7.34
7.33
7.30
7.29
7.28
7.26 CDCl3
7.12
7.11
4.39
4.37
4.32
4.29
NH
O
S
Bn
Br
1h
^{1}H NMR (500 MHz, CDCl$_3$)
4.23
0.83
2.38
2.12
1.00
0.95
ppm

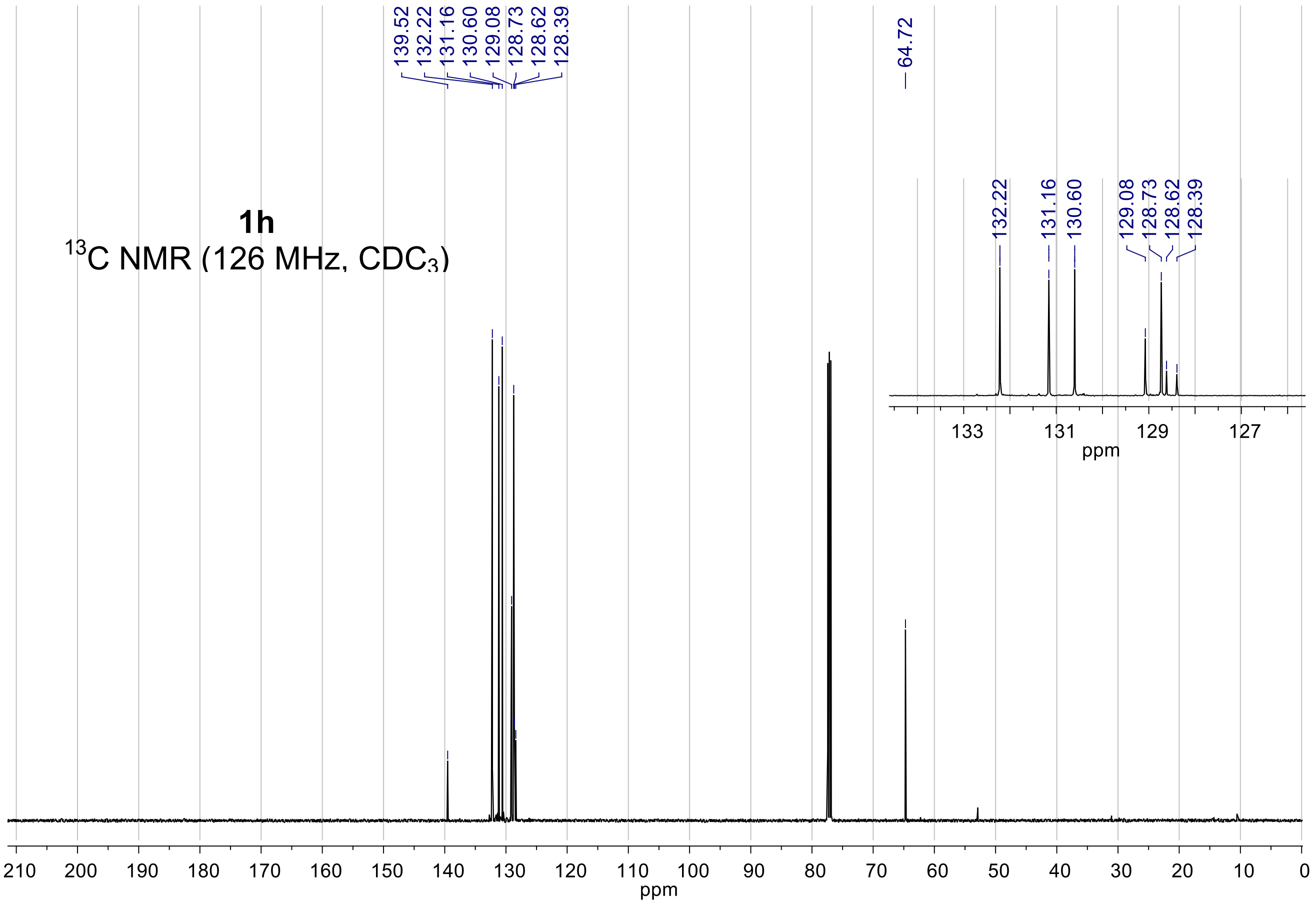
139.52
132.22
131.16
130.60
129.08
128.73
128.62
128.39
64.72
1h
^{13}C NMR (126 MHz, CDC$_3$)
ppm

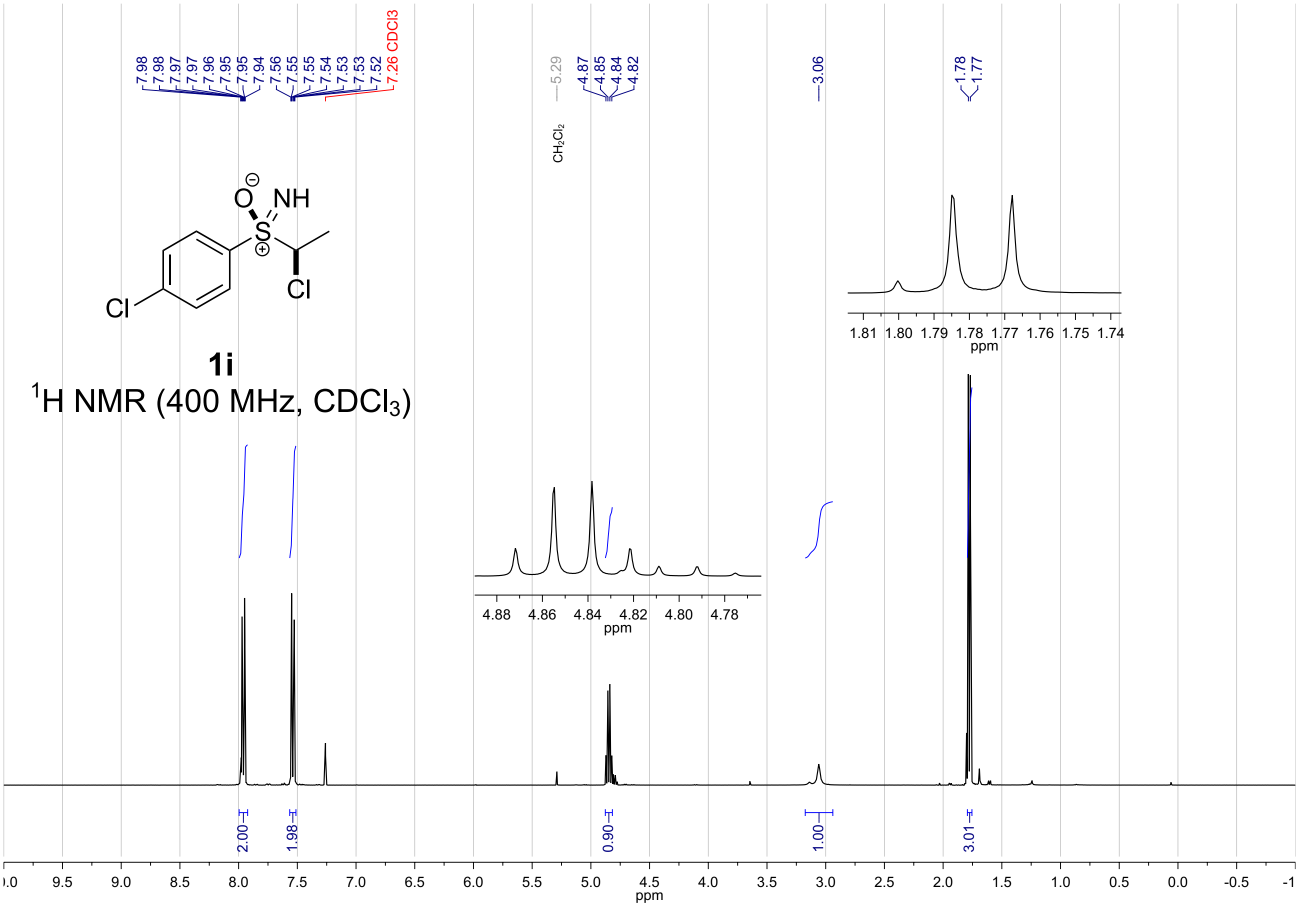
7.98
7.98
7.97
7.97
7.96
7.95
7.95
7.94
7.56
7.55
7.55
7.54
7.53
7.53
7.52
7.26 CDCl3
5.29
CH_2Cl_2
4.87
4.85
4.84
4.82
3.06
1.78
1.77
O
NH
S
Cl
Cl
1i
^1H NMR (400 MHz, $CDCl_3$)
2.00
1.98
0.90
1.00
3.01
ppm

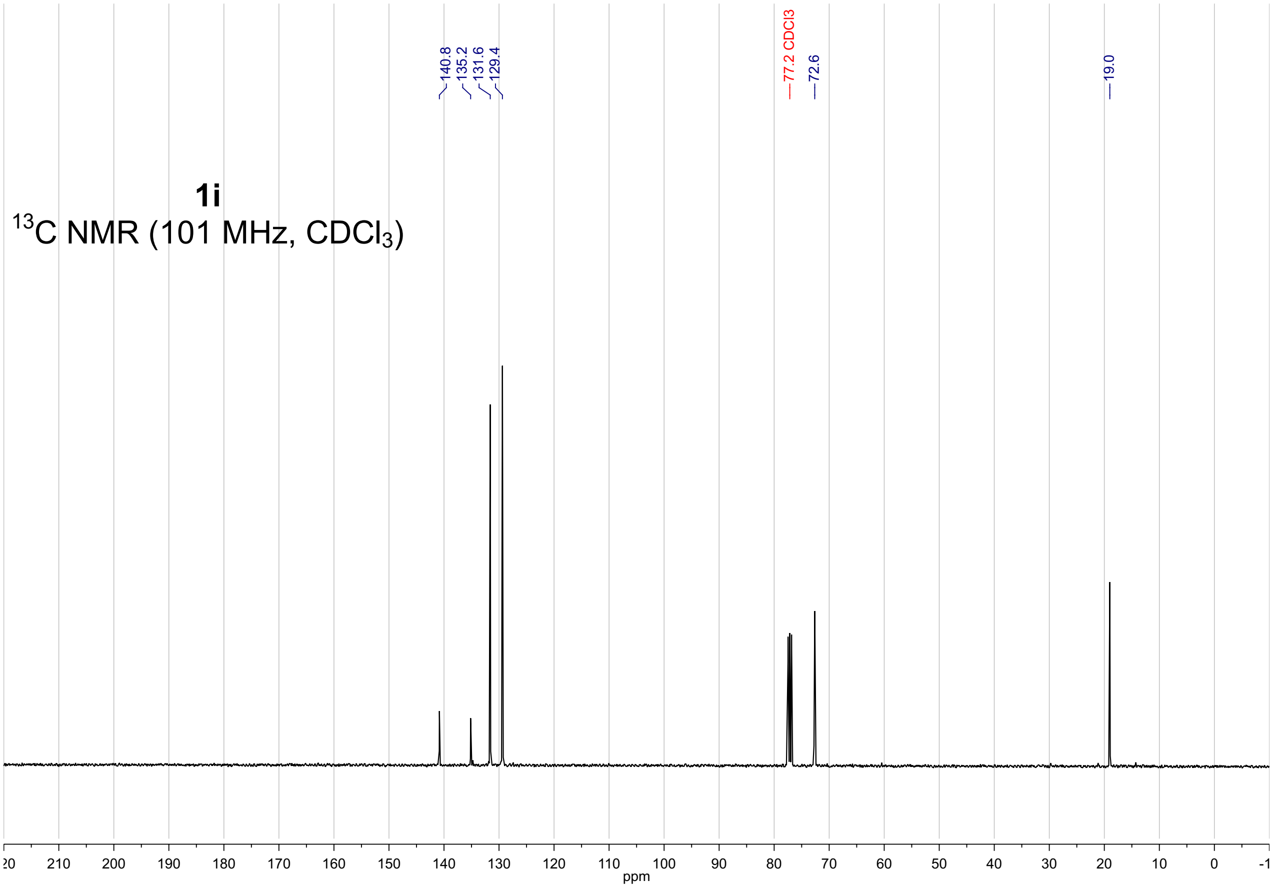
140.8
135.2
131.6
129.4
77.2 CDCl3
72.6
19.0
1i
^{13}C NMR (101 MHz, $CDCl_3$)
ppm

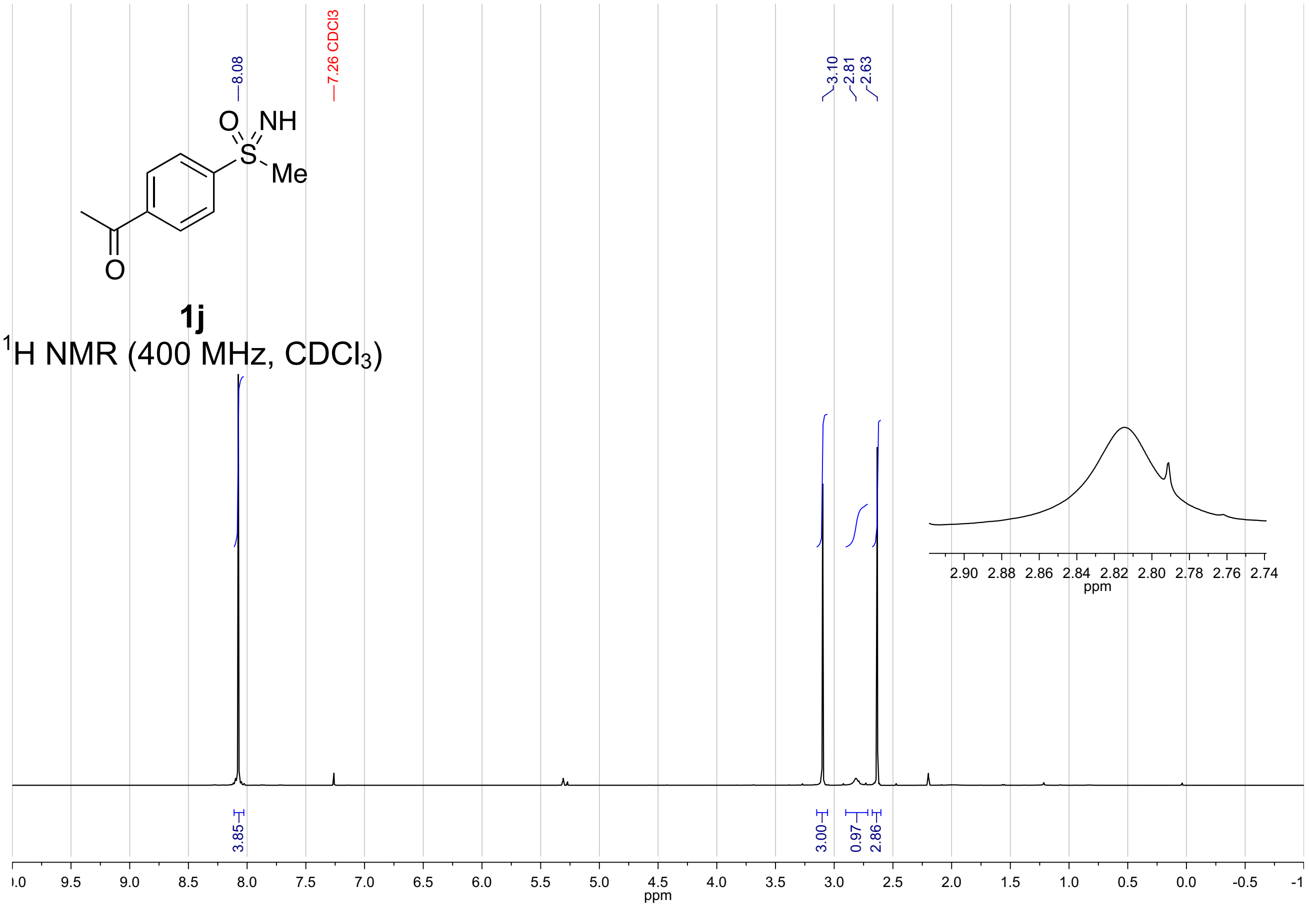
8.08
7.26 CDCl3
3.10
2.81
2.63
O NH
S
Me
O
1j
^{1}H NMR (400 MHz, CDCl$_3$)
3.85
3.00
0.97
2.86
ppm

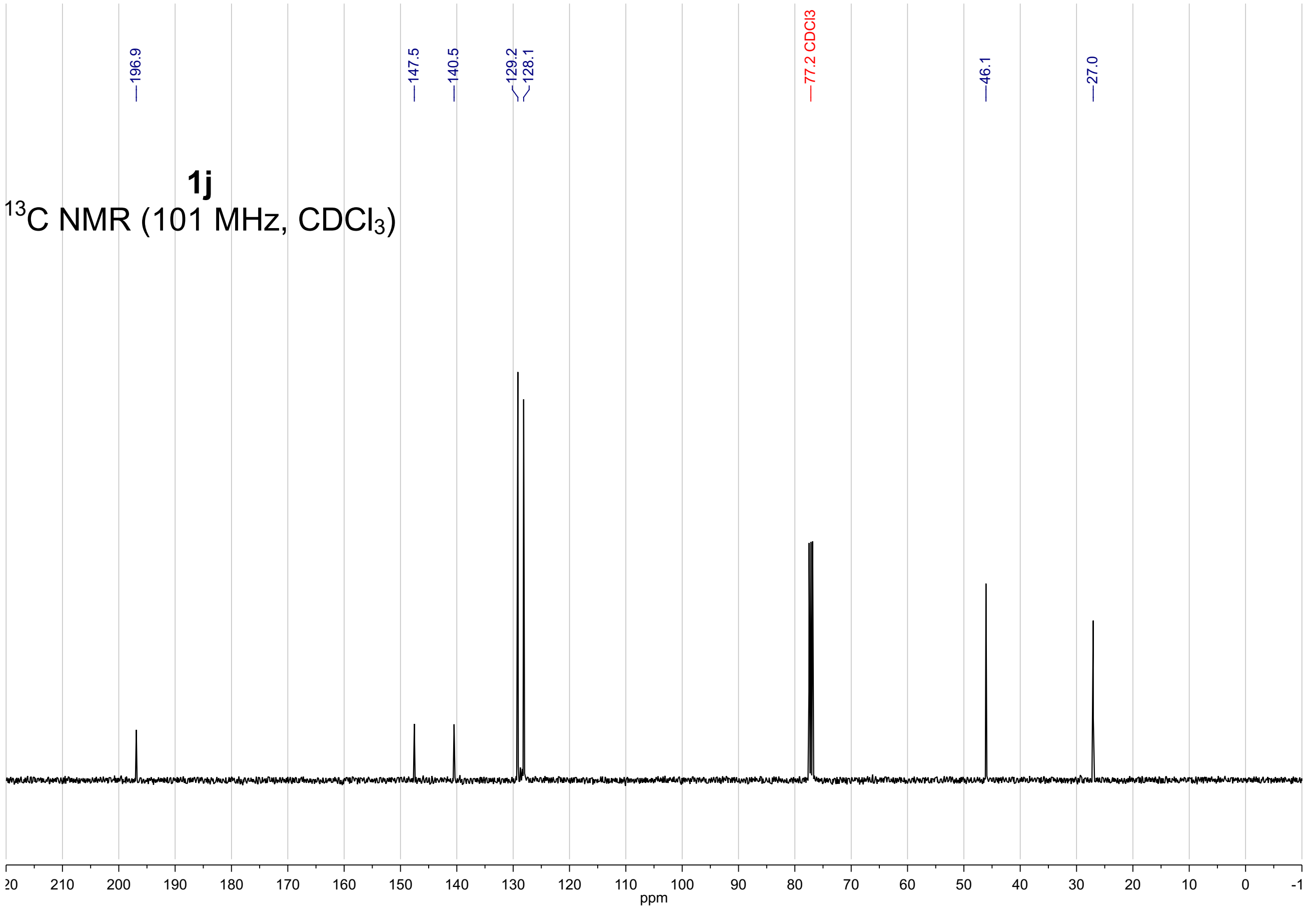
196.9
147.5
140.5
129.2
128.1
77.2 CDCl3
46.1
27.0
1j
^{13}C NMR (101 MHz, CDCl$_3$)
ppm

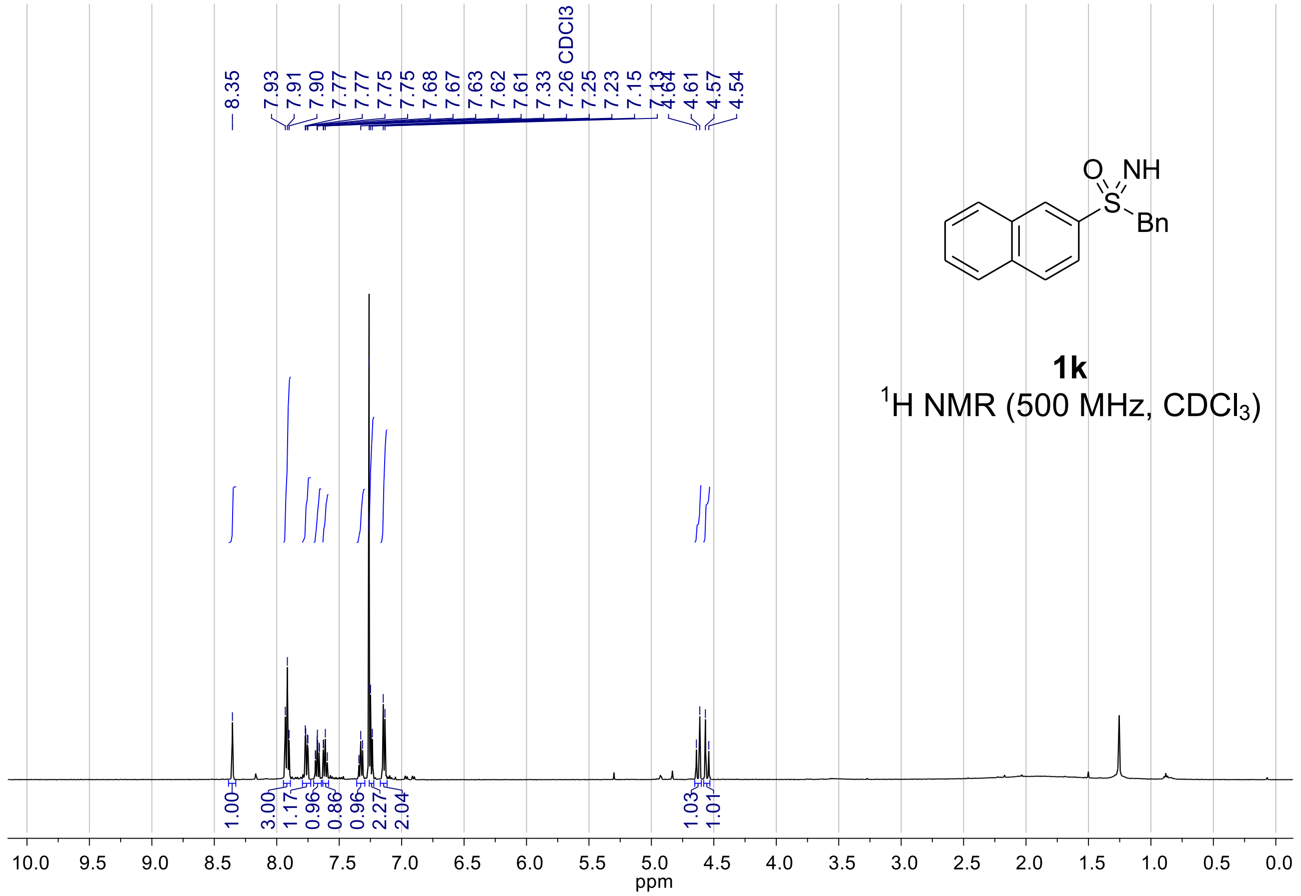

1k

^{1}H NMR (500 MHz, $CDCl_3$)

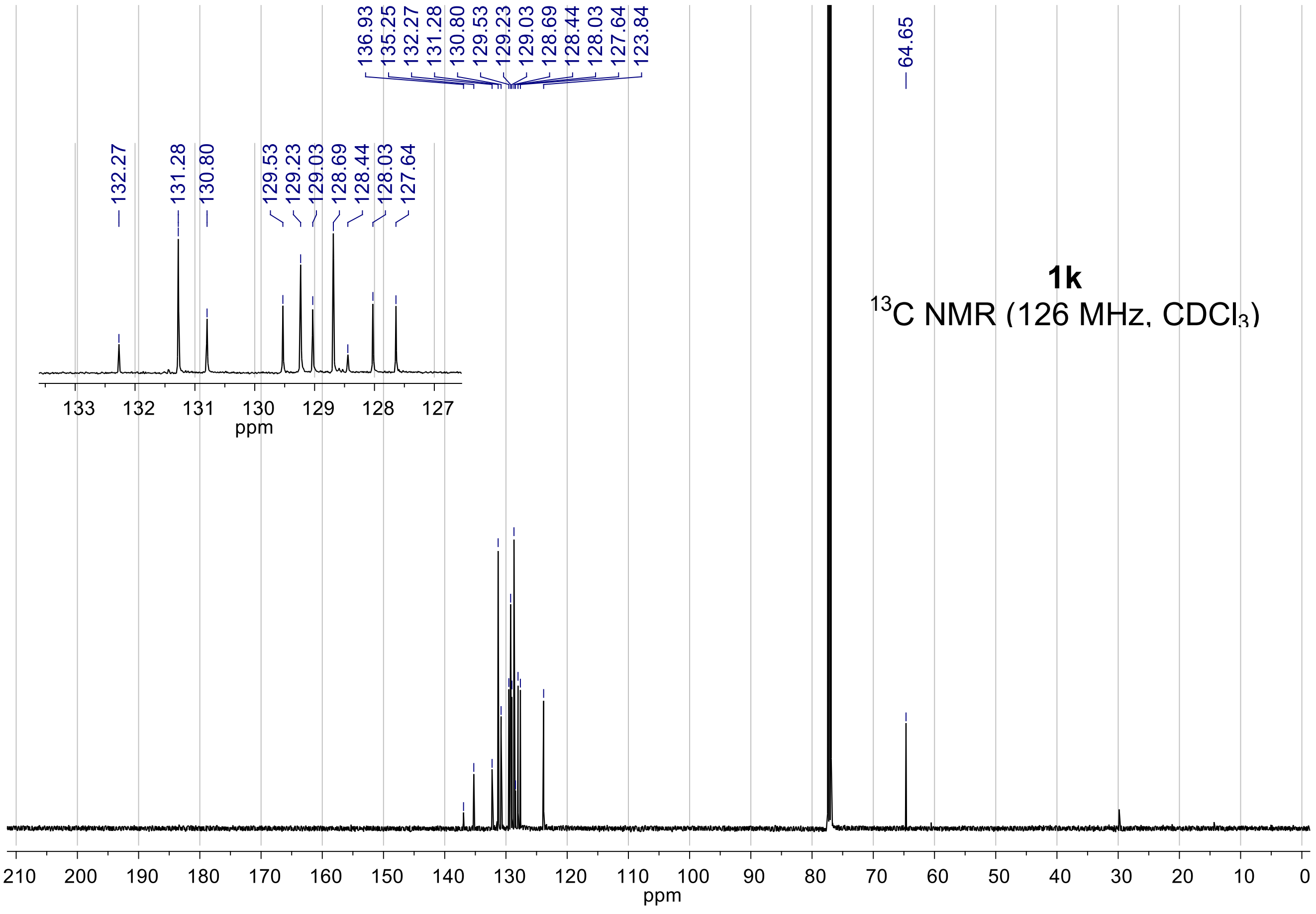

1k

^{13}C NMR (126 MHz, $CDCl_3$)

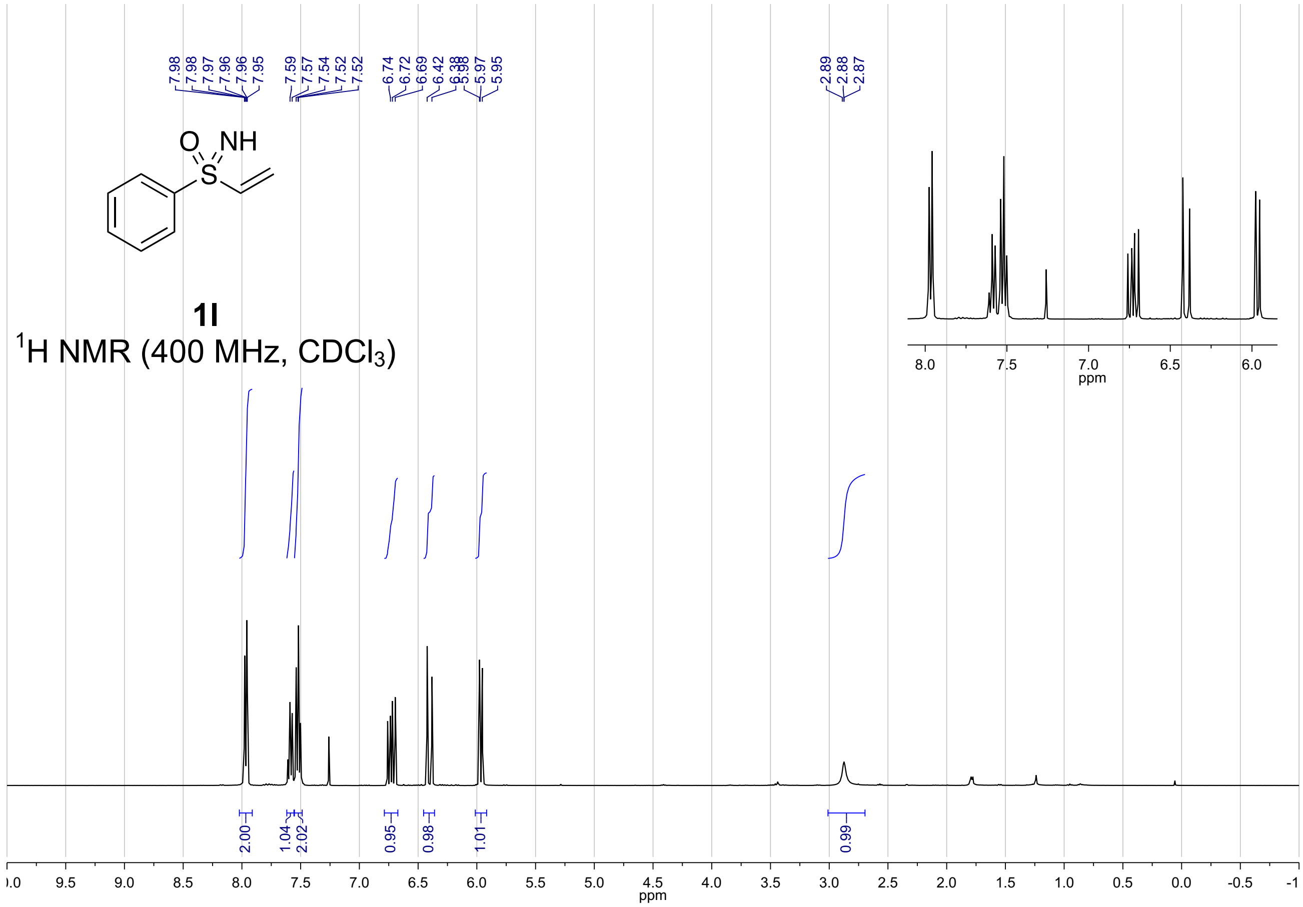
O NH
S
1l
^{1}H NMR (400 MHz, $CDCl_3$)
ppm

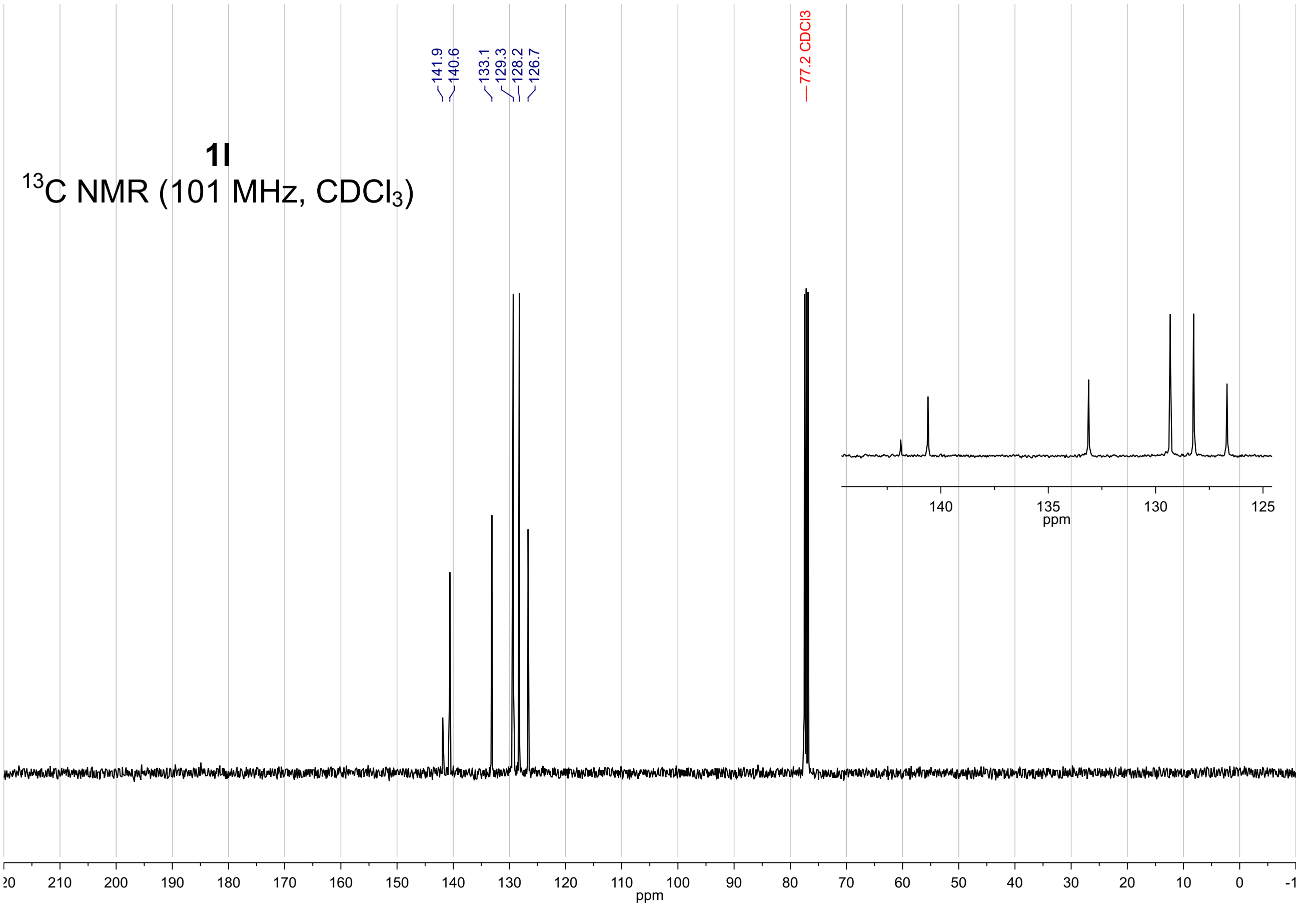
1l
^{13}C NMR (101 MHz, $CDCl_3$)
77.2 CDCl3
ppm

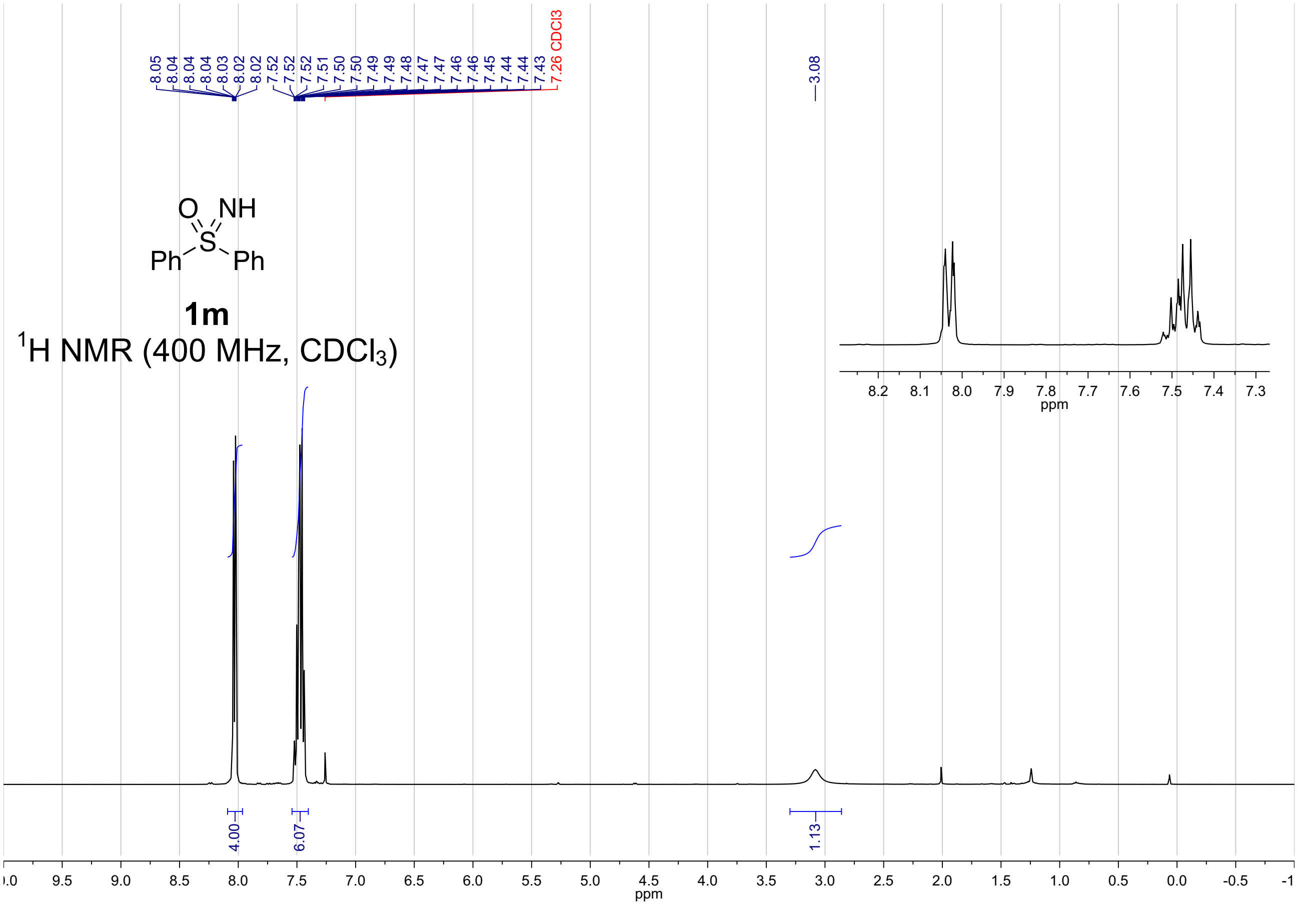
8.05
8.04
8.04
8.04
8.03
8.02
8.02
7.52
7.52
7.52
7.51
7.50
7.50
7.49
7.49
7.48
7.47
7.47
7.46
7.45
7.44
7.44
7.43
7.26 CDCl3
3.08
O NH
S
Ph Ph
1m
1H NMR (400 MHz, CDCl3)
4.00
6.07
1.13
ppm

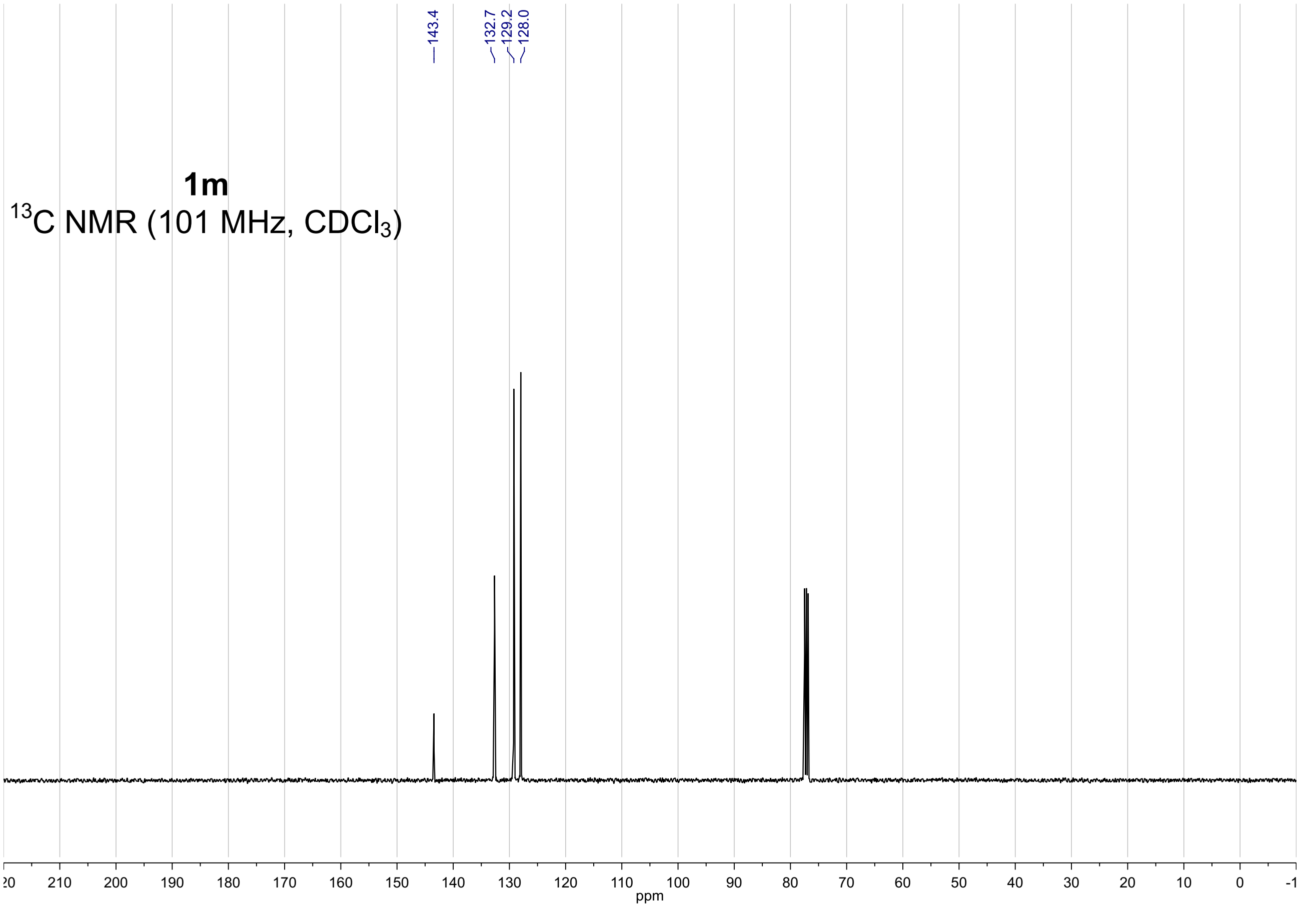
143.4
132.7
129.2
128.0
1m
13C NMR (101 MHz, CDCl3)
ppm

1n

^{1}H NMR (400 MHz, $CDCl_3$)

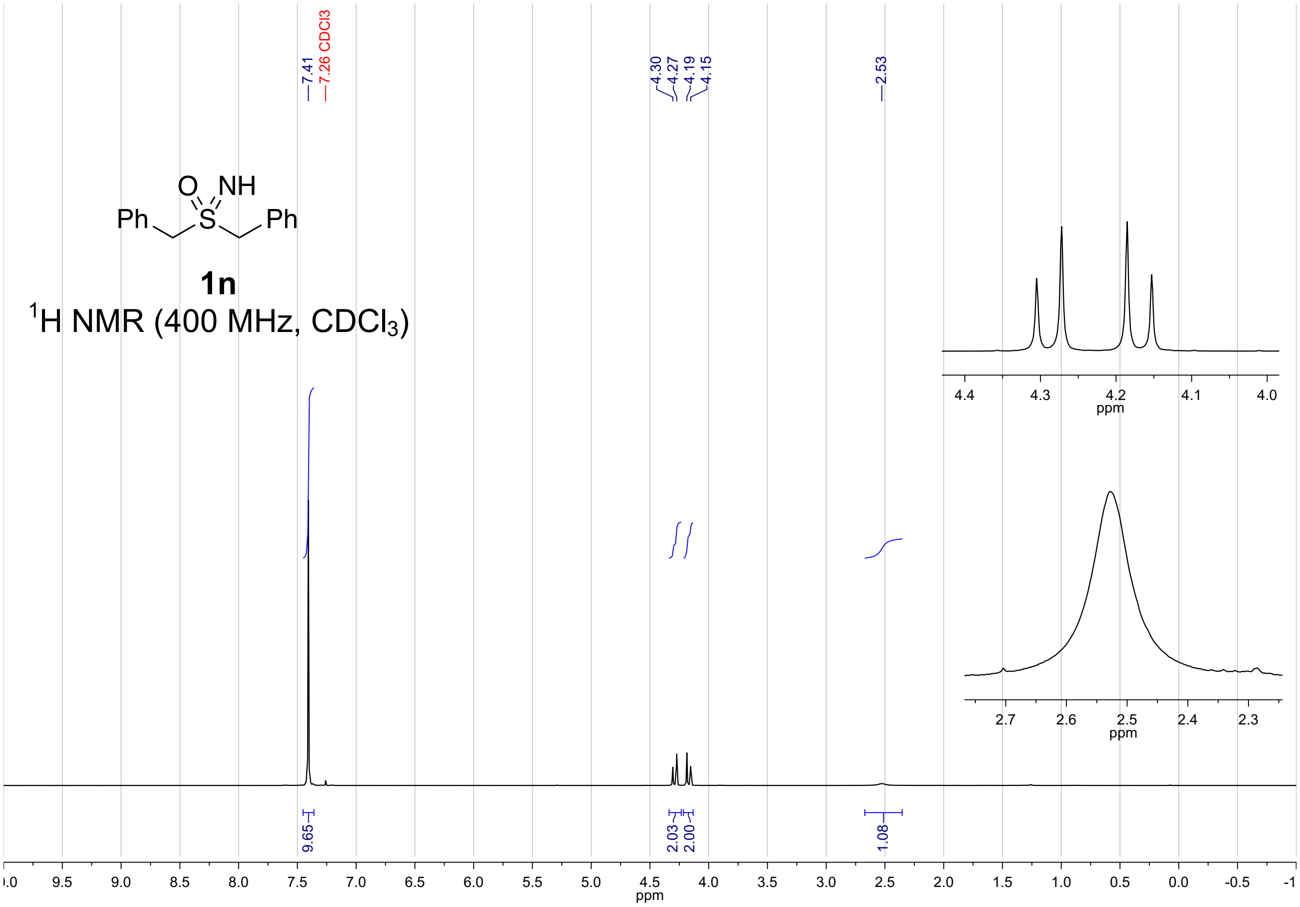

1n

^{13}C NMR (101 MHz, $CDCl_3$)

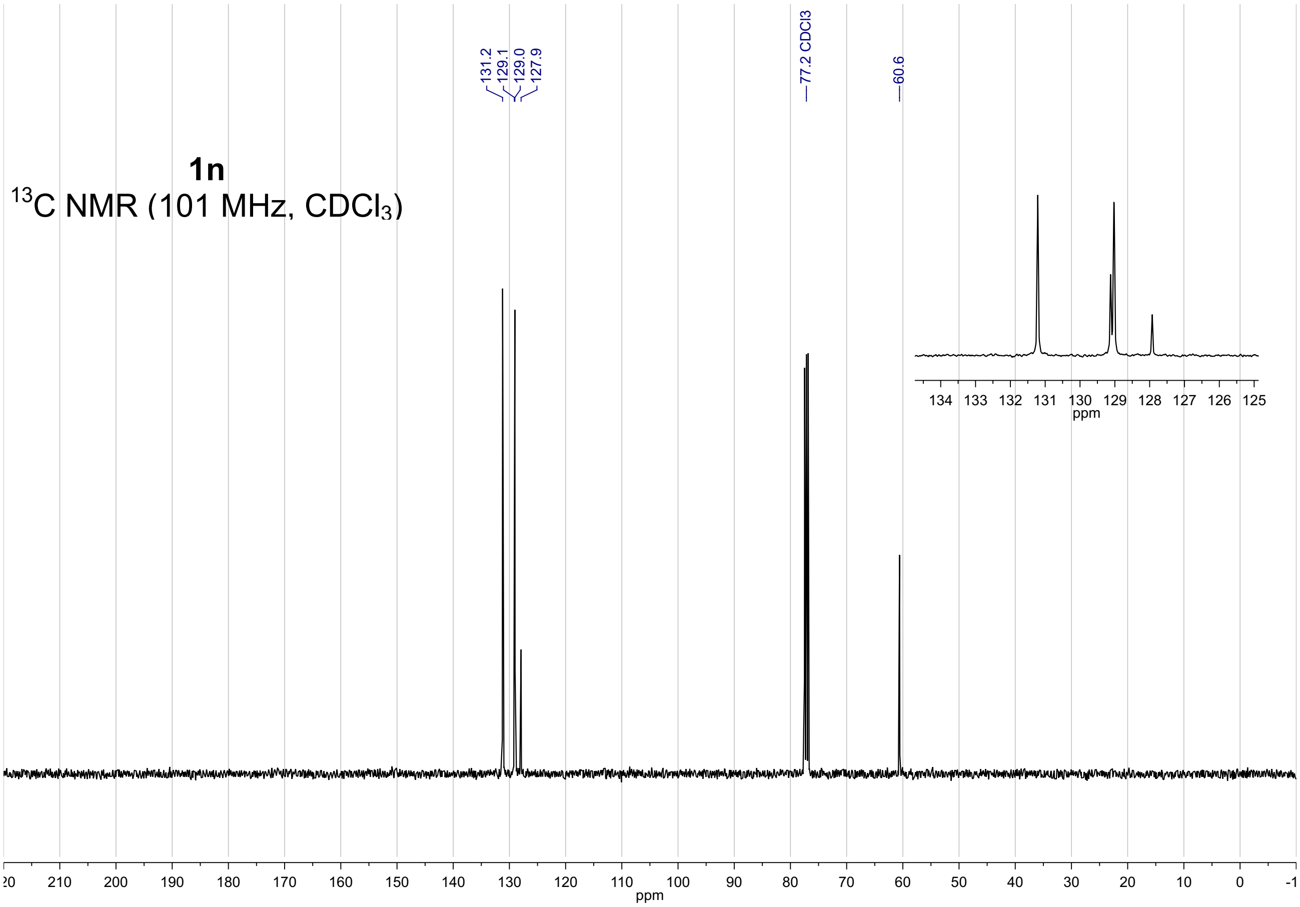

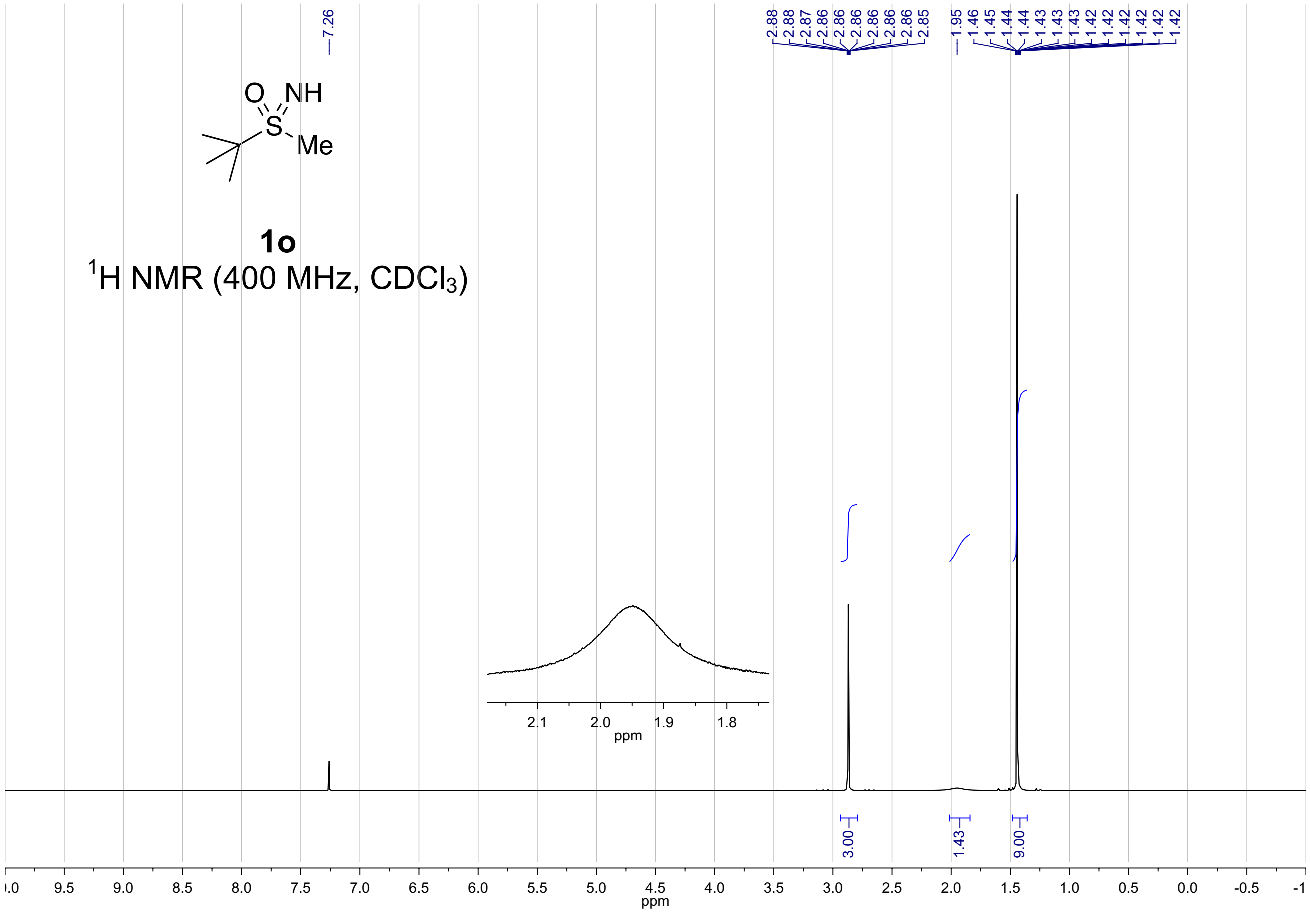

1o
^{1}H NMR (400 MHz, $CDCl_3$)

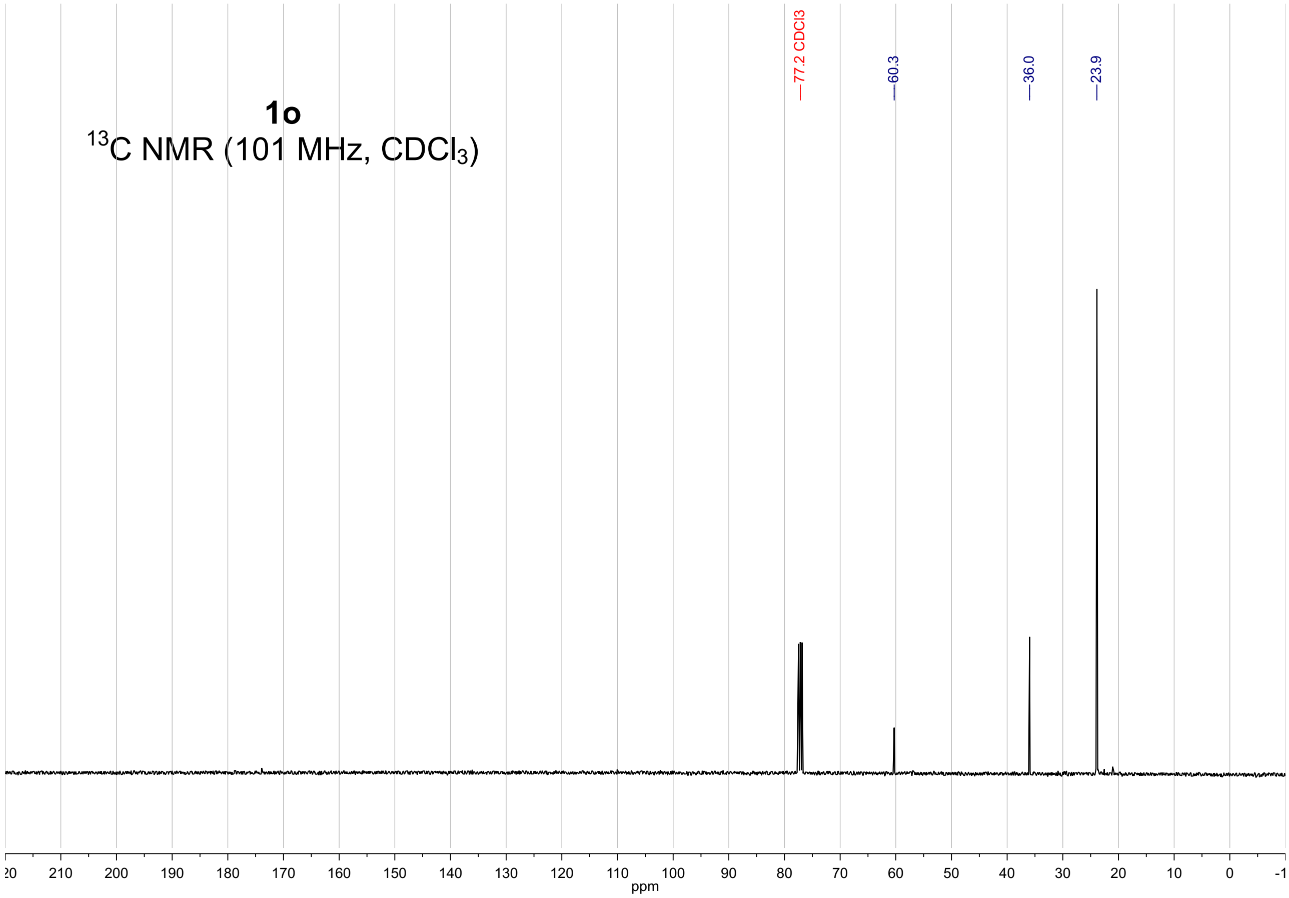

1o
^{13}C NMR (101 MHz, $CDCl_3$)

1p

^{1}H NMR (400 MHz, $CDCl_3$)

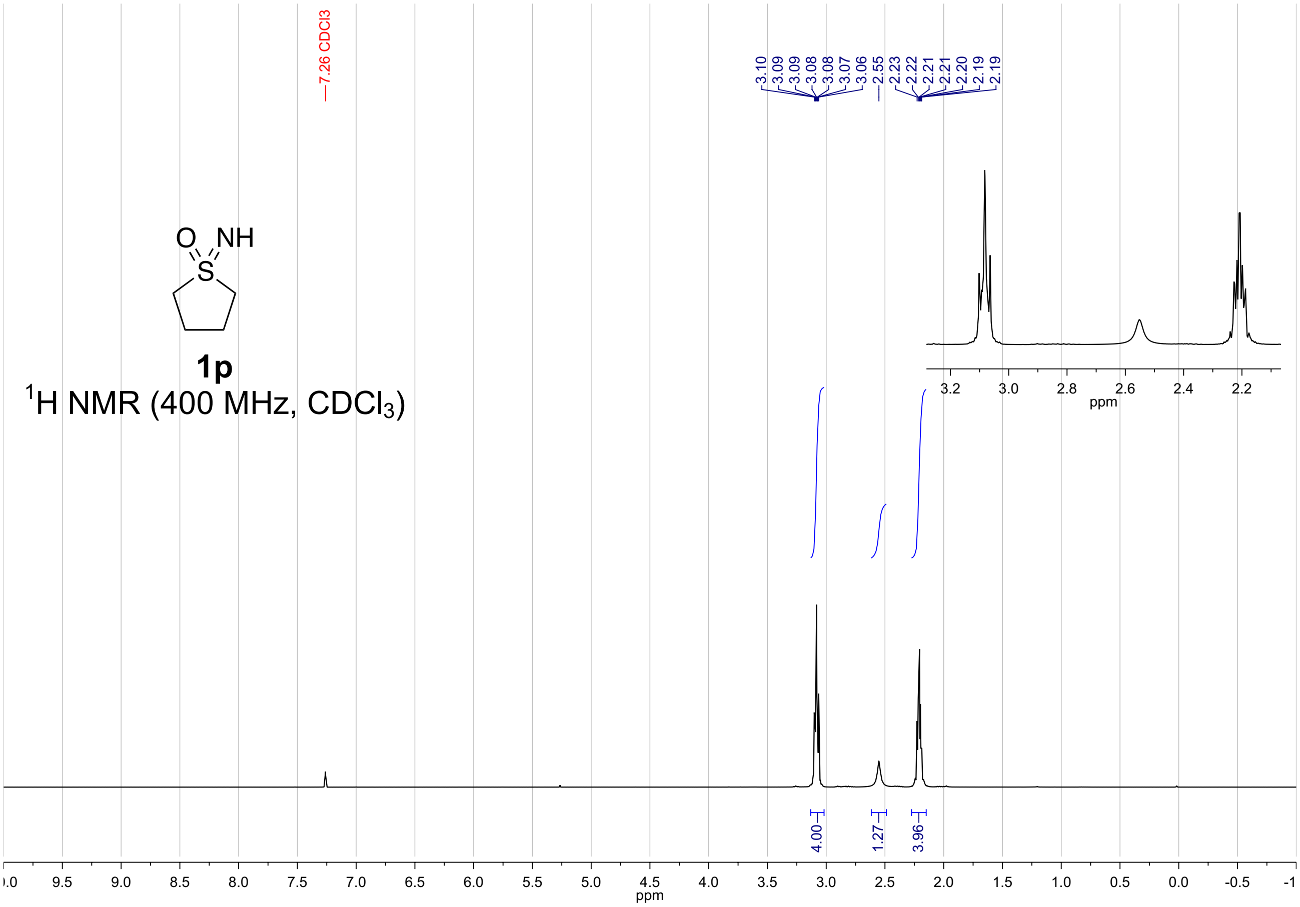

1p

^{13}C NMR (101 MHz, $CDCl_3$)

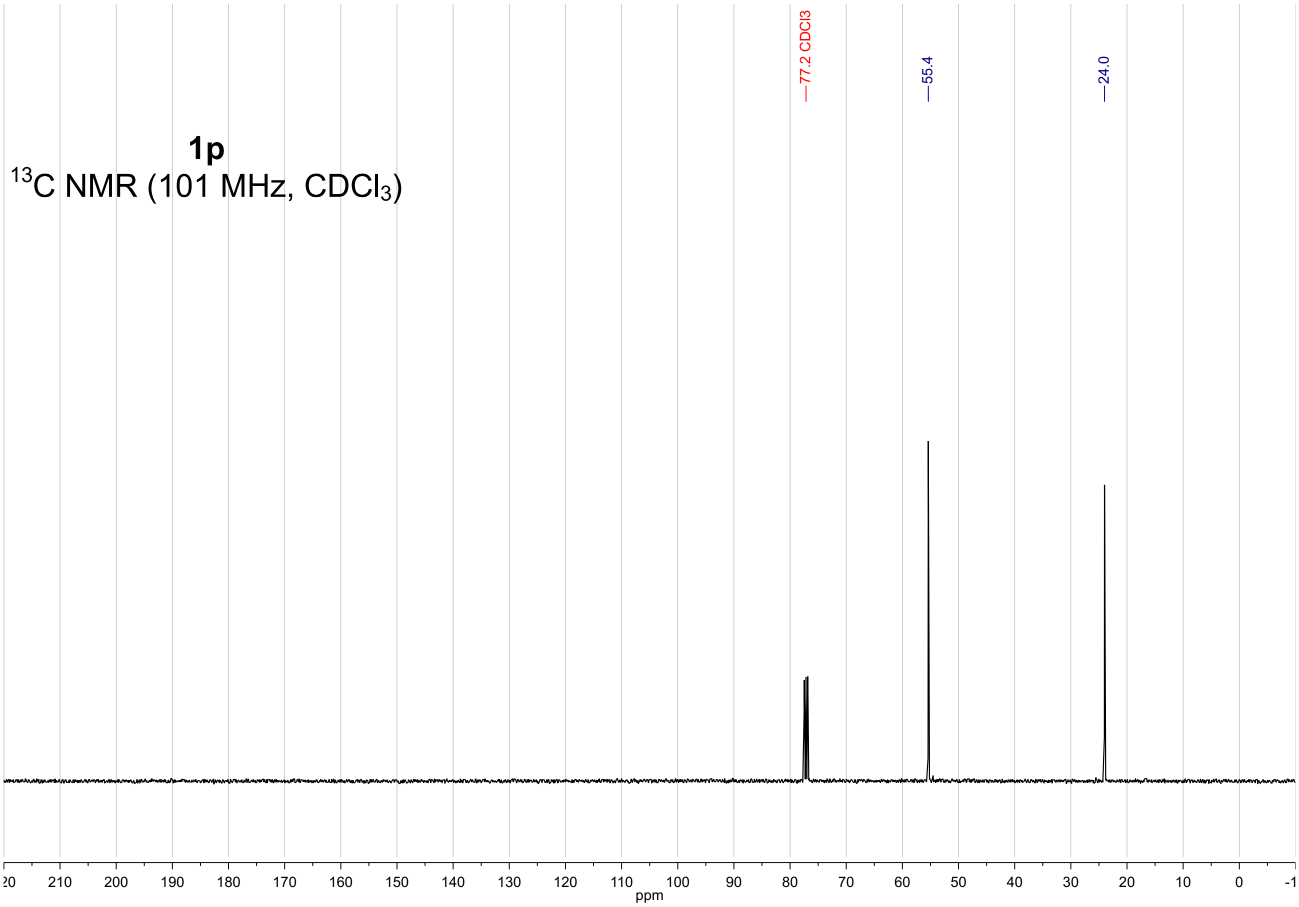

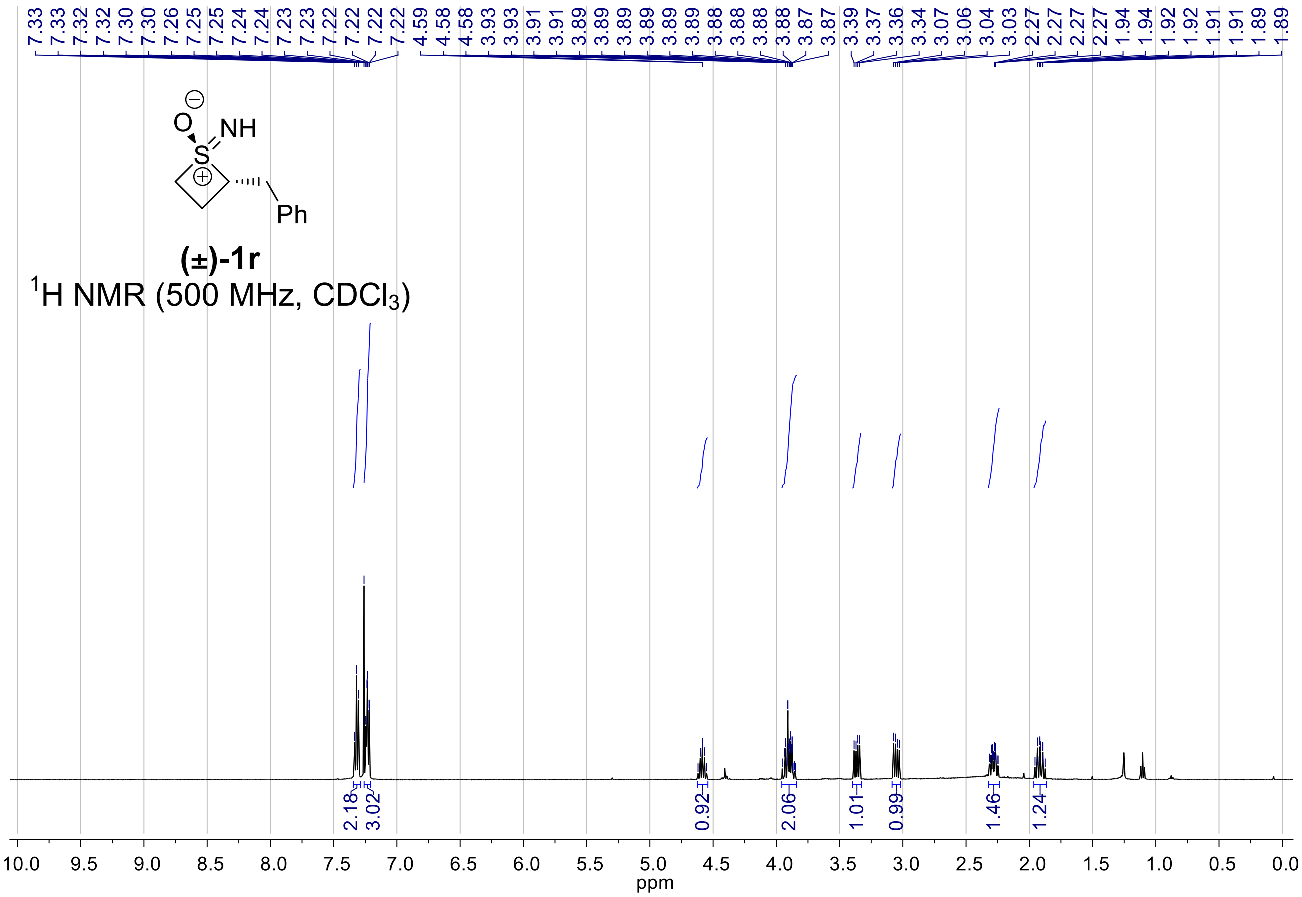
7.33 7.33 7.32 7.32 7.30 7.30 7.26 7.25 7.25 7.24 7.24 7.23 7.23 7.22 7.22 7.22 7.22 4.59 4.58 4.58 3.93 3.93 3.91 3.91 3.89 3.89 3.89 3.89 3.89 3.89 3.88 3.88 3.88 3.88 3.87 3.87 3.39 3.37 3.36 3.34 3.07 3.06 3.04 3.03 2.27 2.27 2.27 2.27 1.94 1.94 1.92 1.92 1.91 1.91 1.89 1.89
O
NH
S
Ph
(±)-1r
^{1}H NMR (500 MHz, $CDCl_3$)
2.18
3.02
0.92
2.06
1.01
0.99
1.46
1.24
10.0 9.5 9.0 8.5 8.0 7.5 7.0 6.5 6.0 5.5 5.0 4.5 4.0 3.5 3.0 2.5 2.0 1.5 1.0 0.5 0.0
ppm

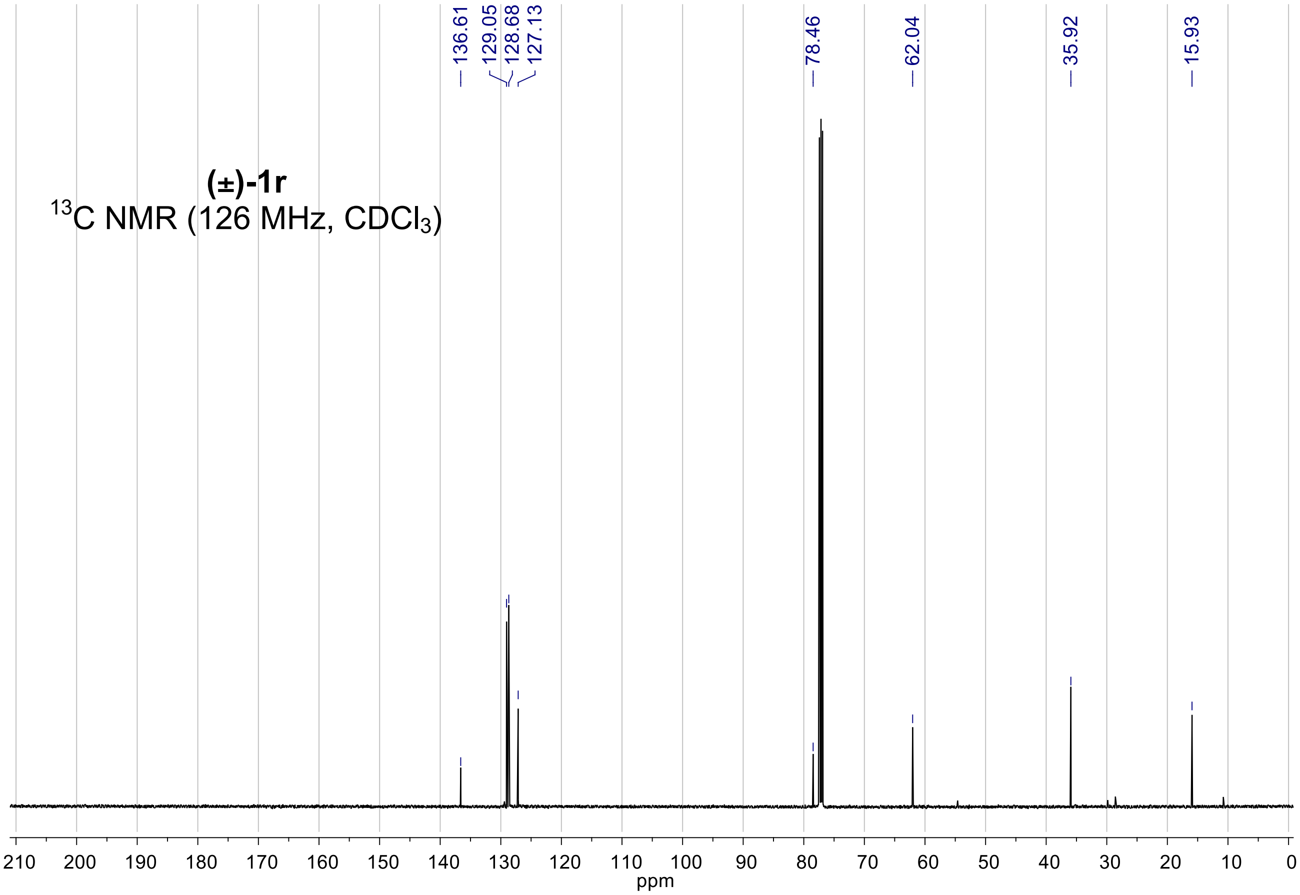
136.61 129.05 128.68 127.13 78.46 62.04 35.92 15.93
(±)-1r
^{13}C NMR (126 MHz, $CDCl_3$)
210 200 190 180 170 160 150 140 130 120 110 100 90 80 70 60 50 40 30 20 10 0
ppm

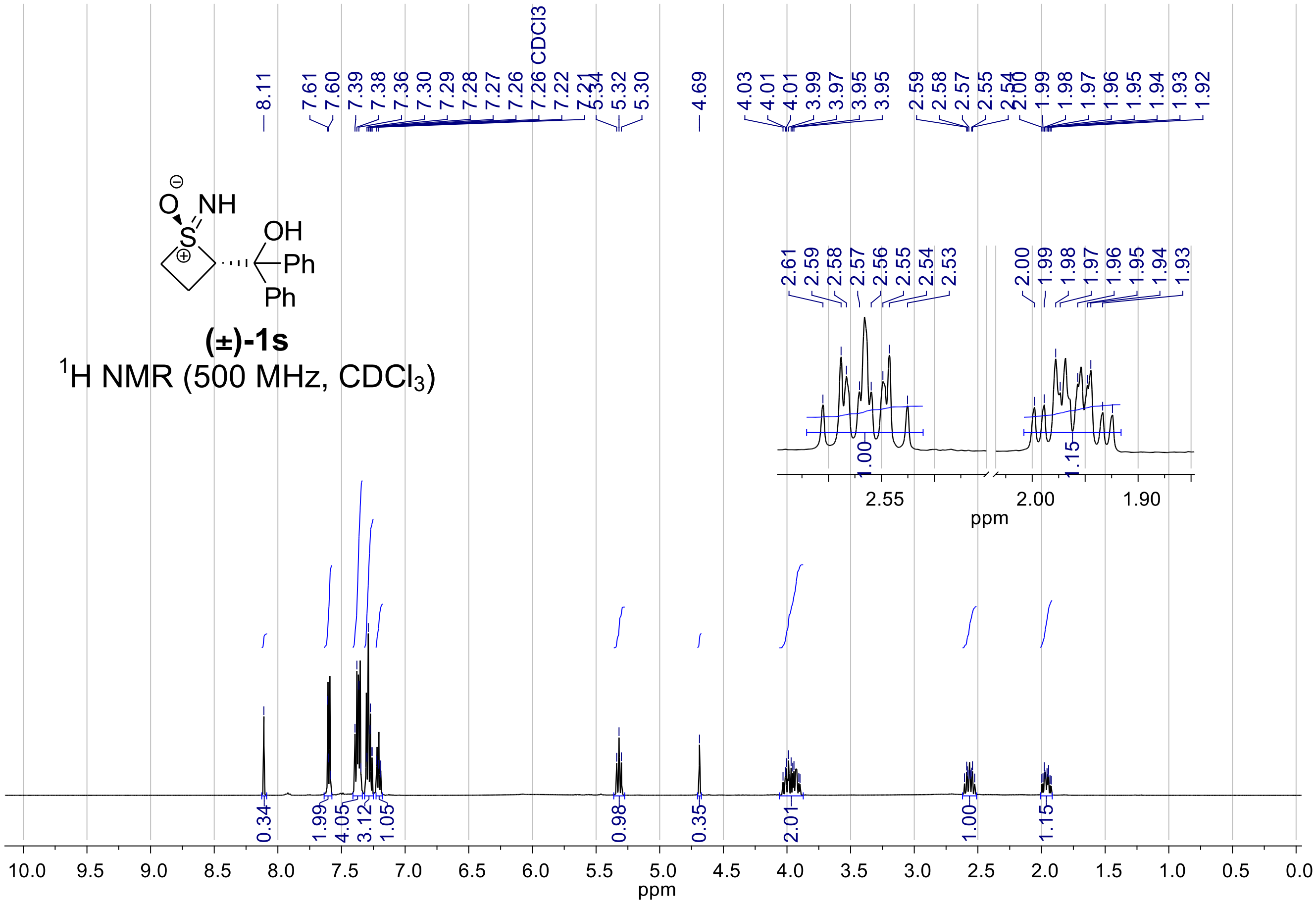
O
NH
S
OH
Ph
Ph
(±)-1s
^{1}H NMR (500 MHz, CDCl$_3$)
8.11
7.61 7.60 7.39 7.38 7.36 7.30 7.29 7.28 7.27 7.26 7.26 CDCl3 7.22 7.21
5.34 5.32 5.30
4.69
4.03 4.01 4.01 3.99 3.97 3.95 3.95
2.59 2.58 2.57 2.55 2.54
2.00 1.99 1.98 1.97 1.96 1.95 1.94 1.93 1.92
2.61 2.59 2.58 2.57 2.56 2.55 2.54 2.53
2.00 1.99 1.98 1.97 1.96 1.95 1.94 1.93
1.00
1.15
2.55
2.00
1.90
ppm
0.34
1.99
4.05
3.12
1.05
0.98
0.35
2.01
1.00
1.15
10.0 9.5 9.0 8.5 8.0 7.5 7.0 6.5 6.0 5.5 5.0 4.5 4.0 3.5 3.0 2.5 2.0 1.5 1.0 0.5 0.0
ppm

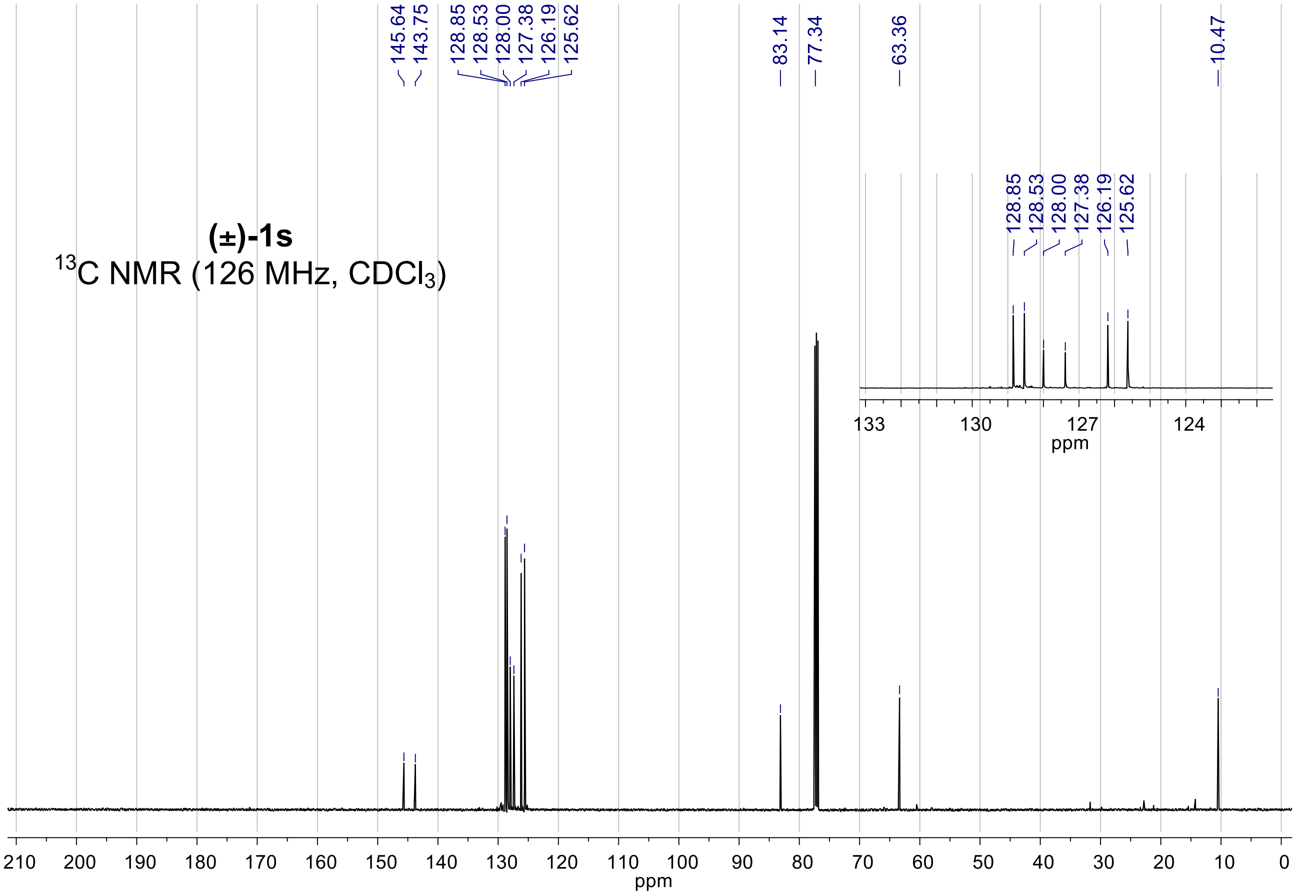
145.64
143.75
128.85 128.53 128.00 127.38 126.19 125.62
83.14
77.34
63.36
10.47
(±)-1s
^{13}C NMR (126 MHz, CDCl$_3$)
128.85
128.53
128.00
127.38
126.19
125.62
133
130
127
124
ppm
210 200 190 180 170 160 150 140 130 120 110 100 90 80 70 60 50 40 30 20 10 0
ppm

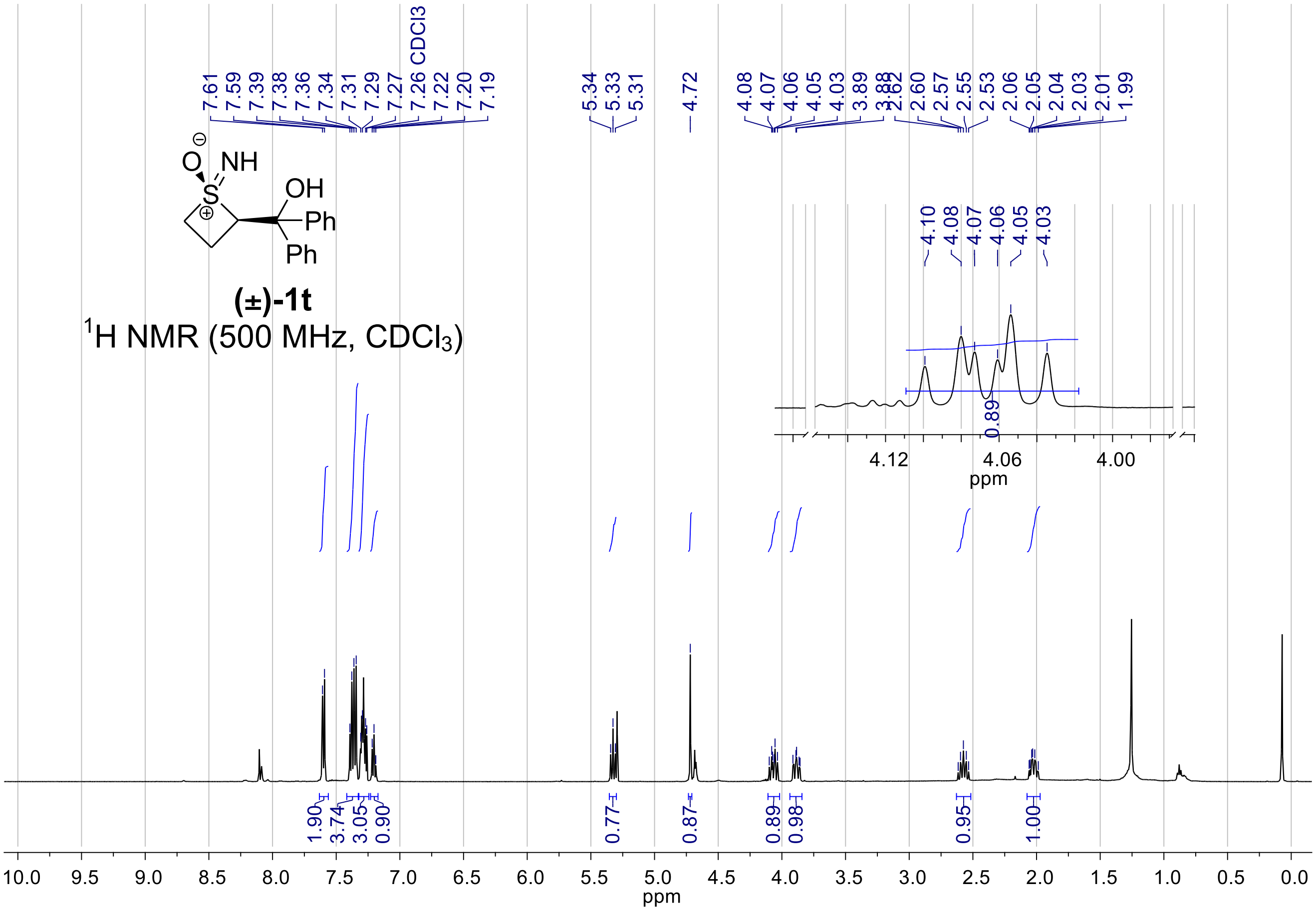

(±)-1t
^{1}H NMR (500 MHz, $CDCl_3$)

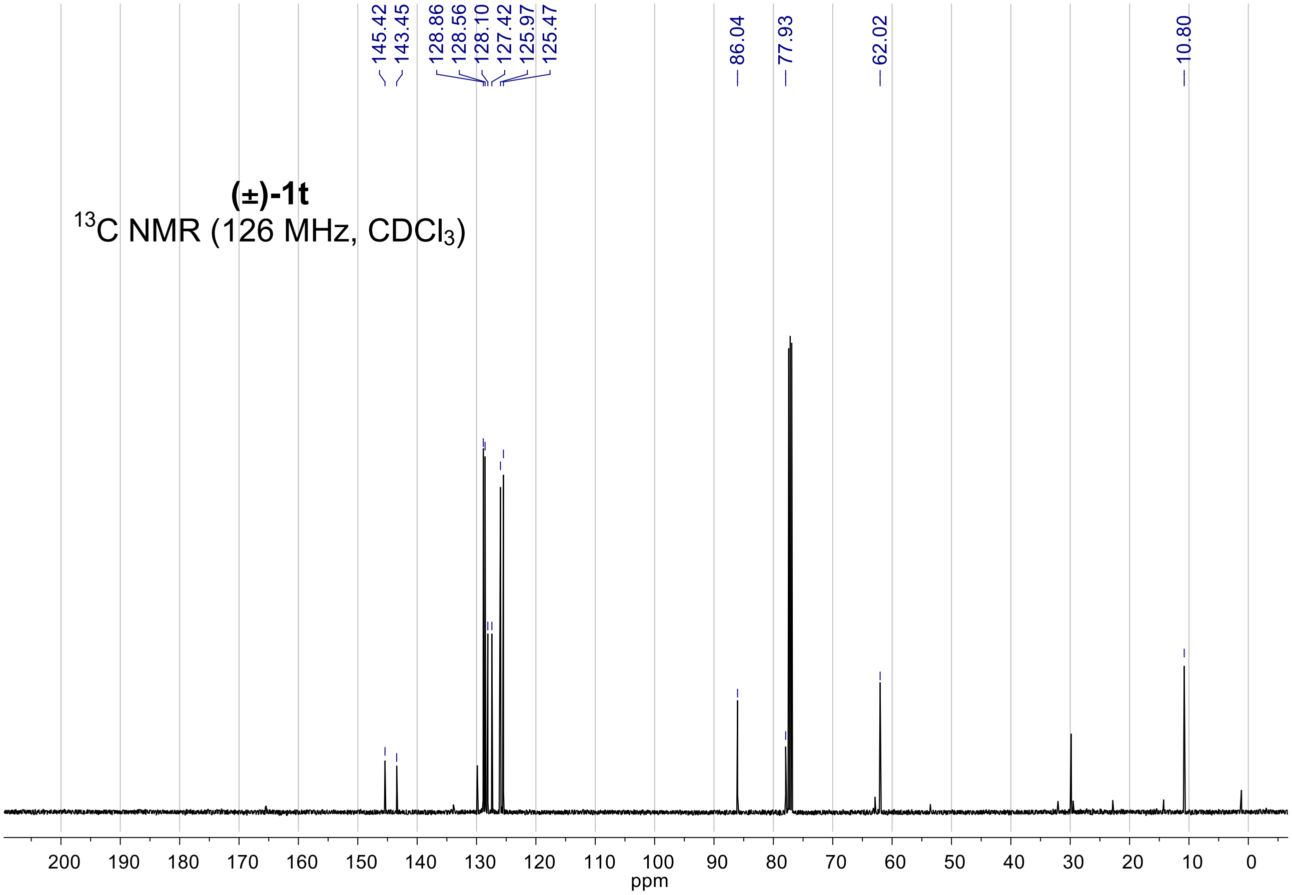

(±)-1t
^{13}C NMR (126 MHz, $CDCl_3$)

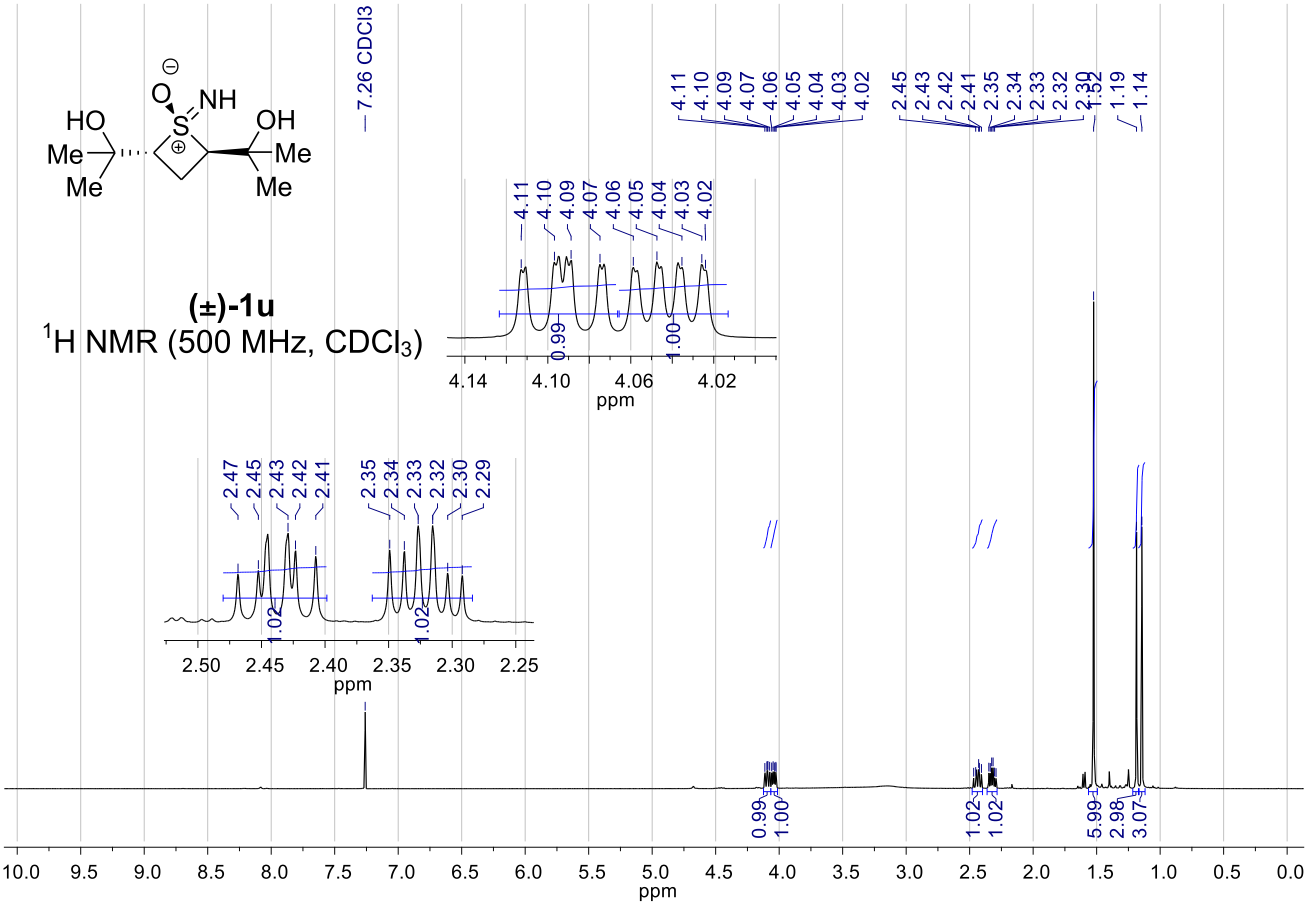
(±)-1u
^{1}H NMR (500 MHz, CDCl$_3$)
7.26 CDCl3
4.11 4.10 4.09 4.07 4.06 4.05 4.04 4.03 4.02
2.45 2.43 2.42 2.41 2.35 2.34 2.33 2.32 2.30 1.52 1.19 1.14
2.47 2.45 2.43 2.42 2.41 2.35 2.34 2.33 2.32 2.30 2.29
0.99 1.00 1.02 1.02 5.99 2.98 3.07
ppm

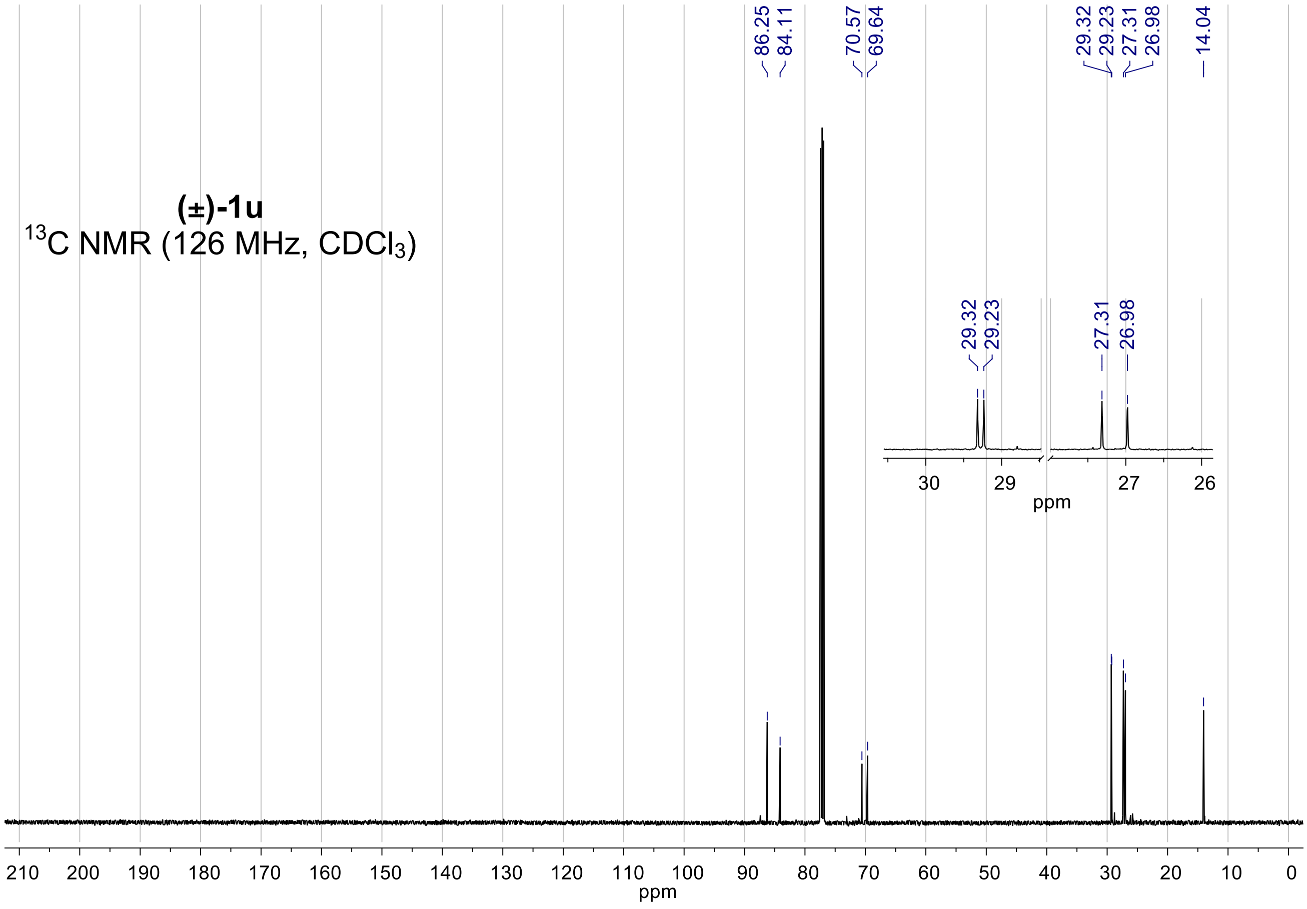
(±)-1u
^{13}C NMR (126 MHz, CDCl$_3$)
86.25 84.11 70.57 69.64 29.32 29.23 27.31 26.98 14.04
ppm

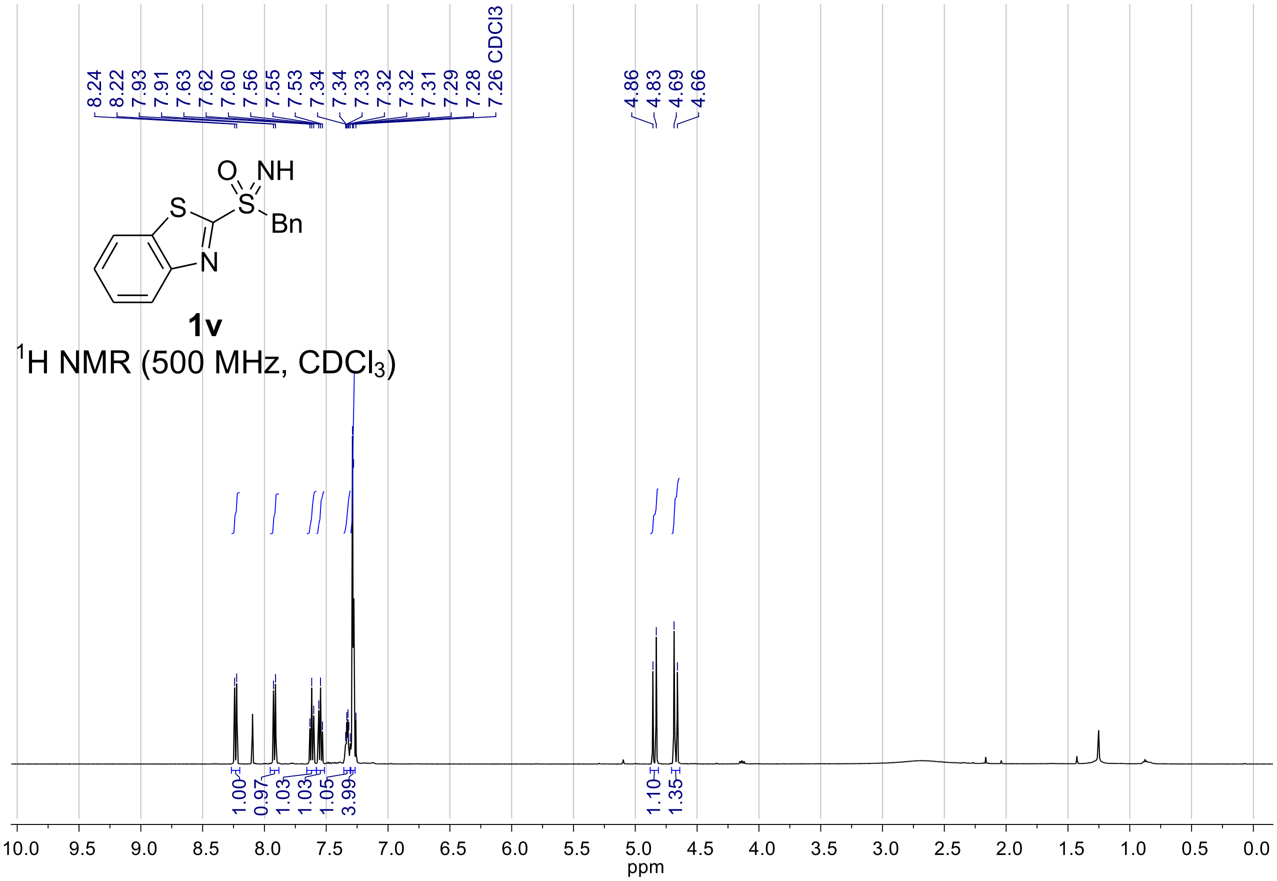
8.24
8.22
7.93
7.91
7.63
7.62
7.60
7.56
7.55
7.53
7.34
7.34
7.33
7.32
7.32
7.31
7.29
7.28
7.26 CDCl3
4.86
4.83
4.69
4.66
O
NH
S
S
Bn
N
1v
1H NMR (500 MHz, CDCl3)
1.00
0.97
1.03
1.03
1.05
3.99
1.10
1.35
10.0 9.5 9.0 8.5 8.0 7.5 7.0 6.5 6.0 5.5 5.0 4.5 4.0 3.5 3.0 2.5 2.0 1.5 1.0 0.5 0.0
ppm

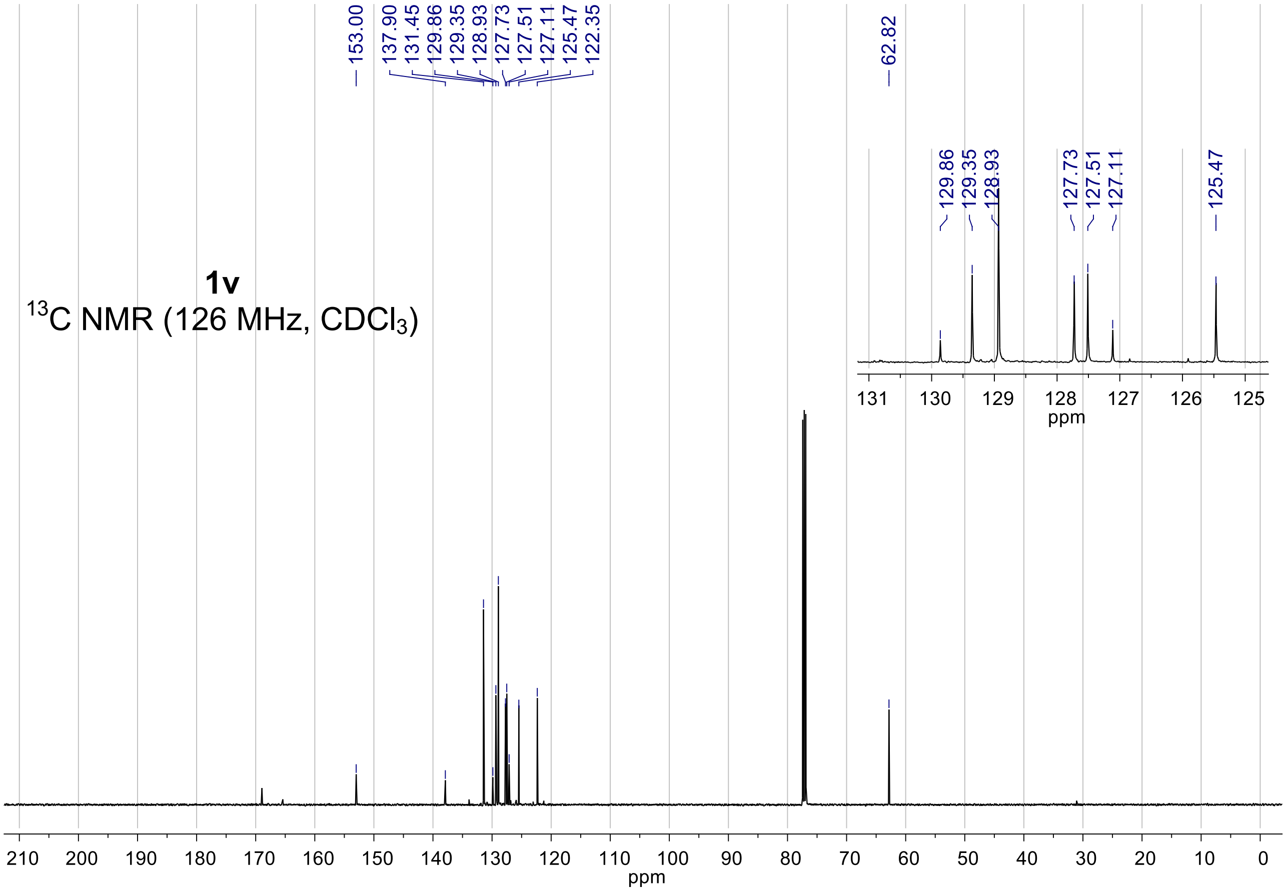
153.00
137.90
131.45
129.86
129.35
128.93
127.73
127.51
127.11
125.47
122.35
62.82
1v
13C NMR (126 MHz, CDCl3)
129.86
129.35
128.93
127.73
127.51
127.11
125.47
131 130 129 128 127 126 125
ppm
210 200 190 180 170 160 150 140 130 120 110 100 90 80 70 60 50 40 30 20 10 0
ppm

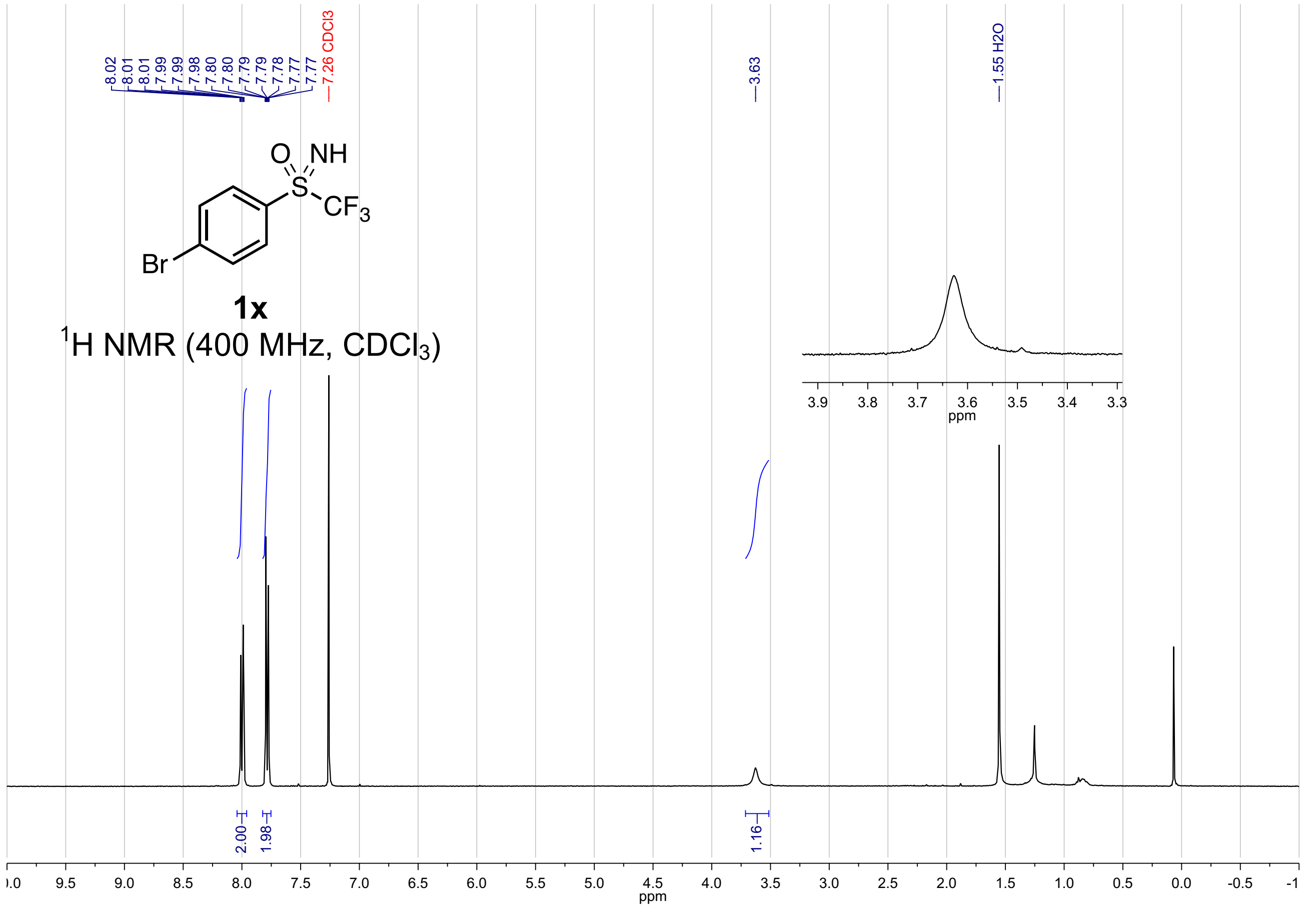
8.02
8.01
8.01
7.99
7.99
7.98
7.80
7.80
7.79
7.79
7.78
7.77
7.77
7.26 CDCl3
3.63
1.55 H2O
1x
^{1}H NMR (400 MHz, $CDCl_3$)
2.00
1.98
1.16
ppm

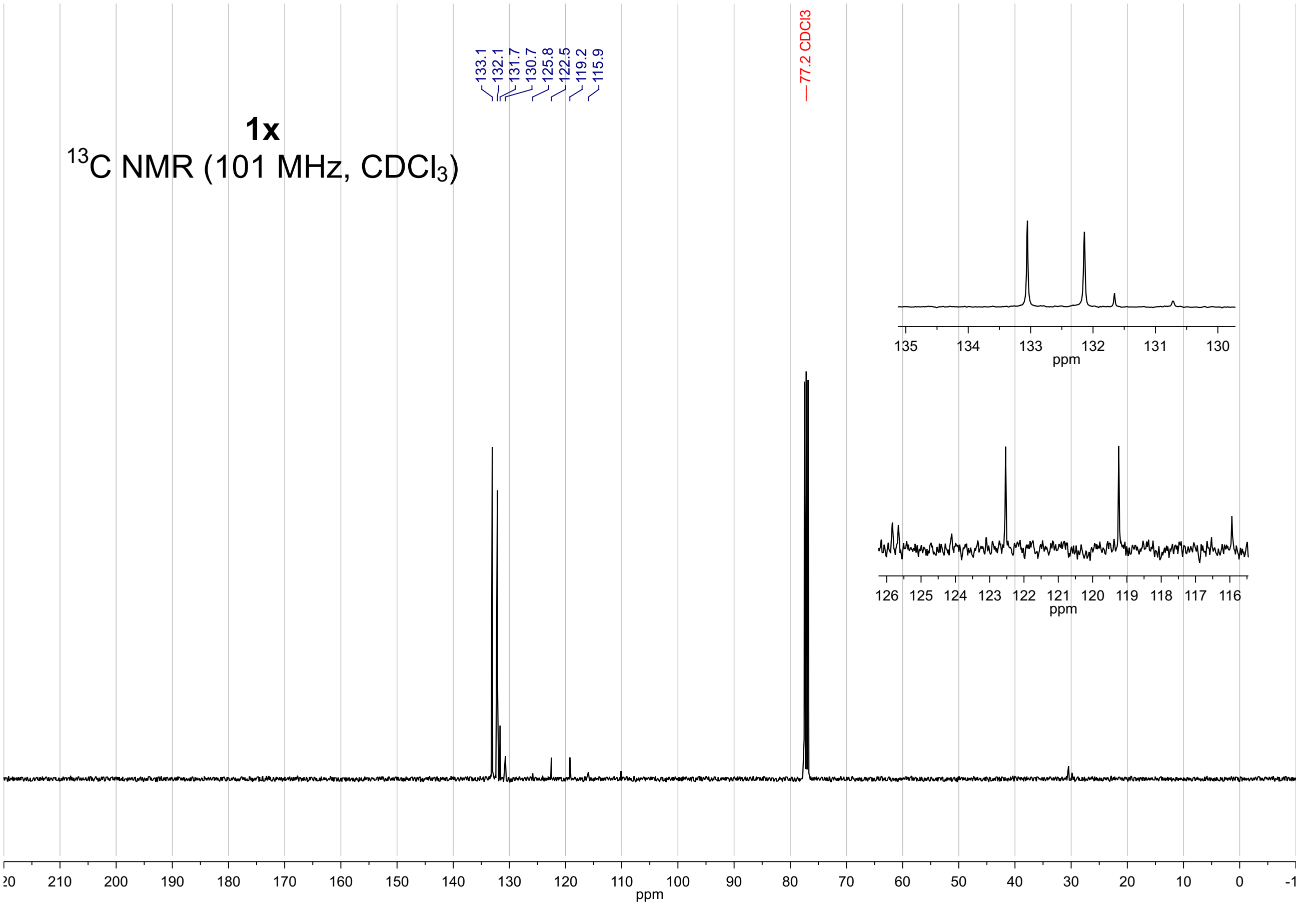
133.1
132.1
131.7
130.7
125.8
122.5
119.2
115.9
77.2 CDCl3
1x
^{13}C NMR (101 MHz, $CDCl_3$)
ppm

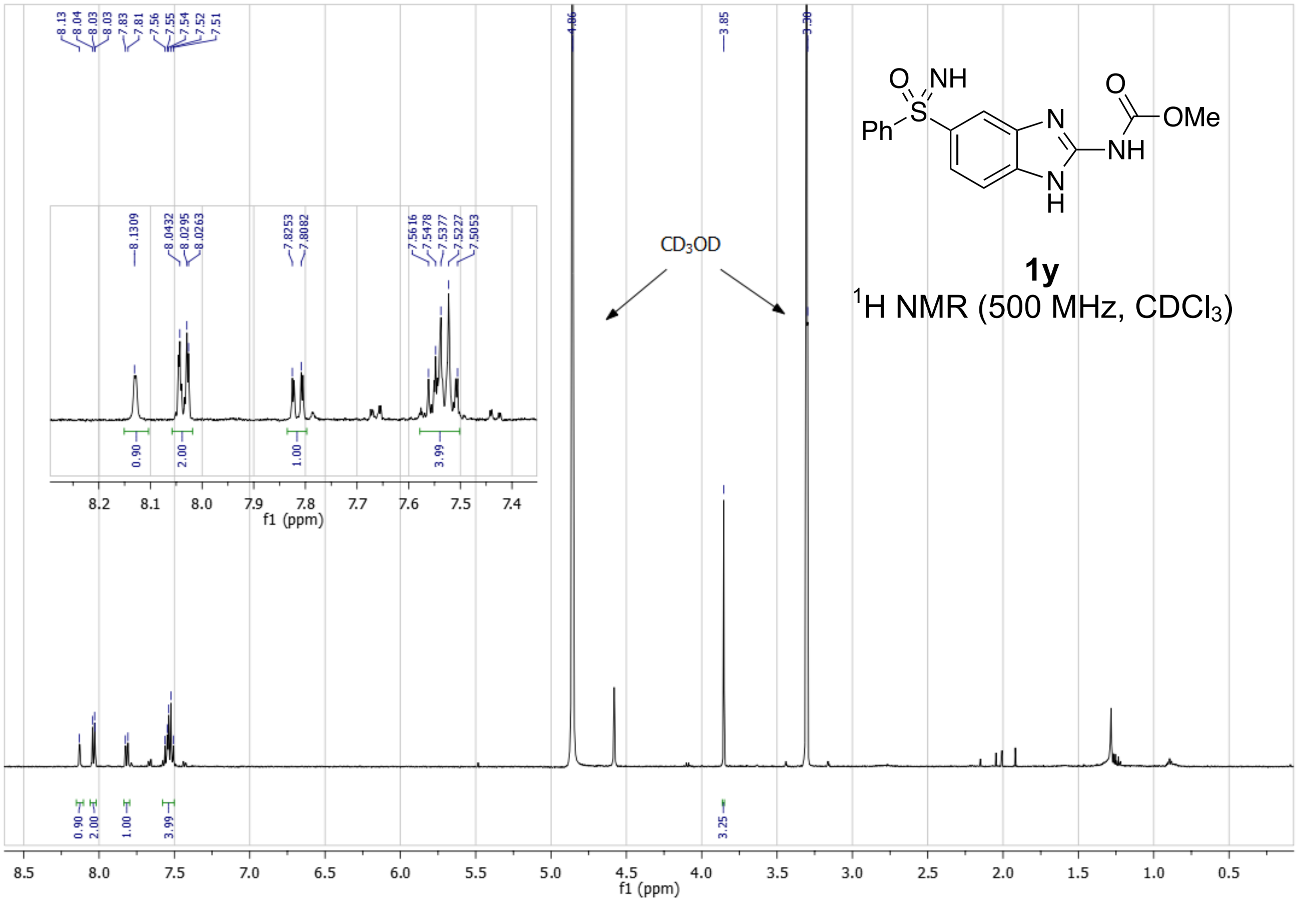

1y

^{1}H NMR (500 MHz, $CDCl_3$)

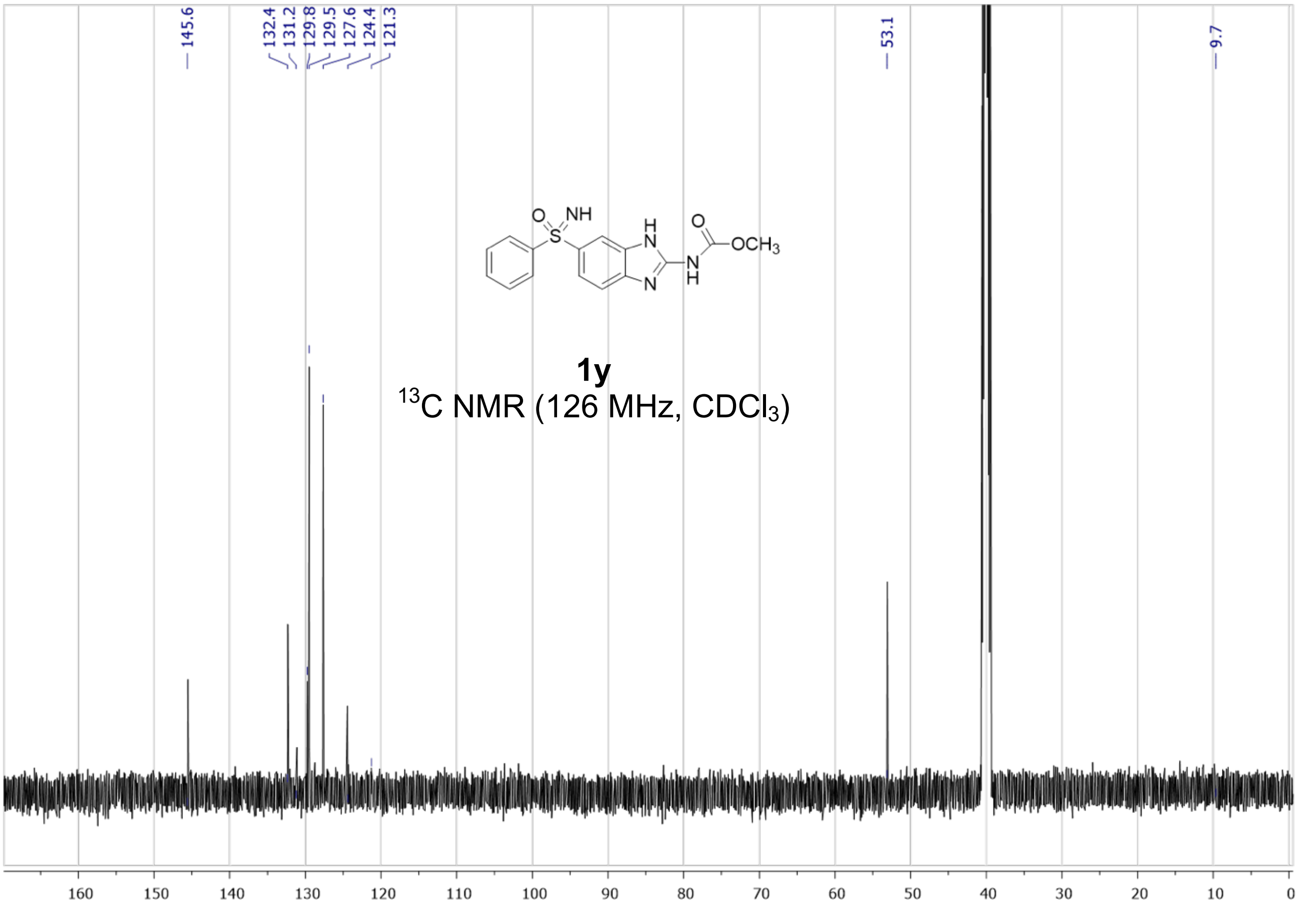

1y

^{13}C NMR (126 MHz, $CDCl_3$)

References

1. Okamura, H.; Bolm, C. *Org. Lett.* **2004**, *6*, 1305-1307.
2. Blakemore, P. R.; Burge, M. S.; Sephton, M. A. *Tetrahedron Lett.*, **2007**, *48*, 3999-4002.
3. Carroccia, L; Degennaro, L; Romanazzi, G.; Cuocci, C.; Pisano, L., Luisi, R. *Org. Biomol. Chem.* **2014**, *12*, 2180-2184.
4. Bharadwaj, S. K.; Shama, S. N.; Hussain, S. H.; Chaudhuri M. K. *Tetrahedron Lett* **2009**, 50, *27*, 3763.
5. Buglioni, L.; Bizet, V.; Bolm, C. *Adv. Synth. Catal.* **2014,** *356*, 2209-2213
6. Ali, M. H.; Stricklin, S. *Synth. Commun.* **2006,** *36*, 1779-1786.
7. Hanson, P.; Hendrickx, R. A. A. J.; Smith, J. R. L. *Org. Biomol. Chem.* **2008,** *6*, 745-761.
8. Capozzi, M. A. M; Centrone, C.; Fracchiolla, G.; Naso; F., Cardellicchio; C. *Eur. J. Org. Chem.* **2011**, 4327-4334.
9. Capozzi, M. A. M; Terraneo, G; Cavallo, G; Cardellicchio; C. *Tetrahedron* **2015**, *71*, 4810-4816.
